# Synthesis, physicochemical characterization and biological activity of novel pyrrole flavones

**DOI:** 10.1038/s41598-025-91772-9

**Published:** 2025-03-03

**Authors:** Stepan Sysak, Barbara Wicher, Malgorzata Kucinska, Paulina Kobylka, Dariusz T. Mlynarczyk, Roman Lesyk, Ewa Tykarska, Marek Murias, Tomasz Goslinski, Wojciech Szczolko

**Affiliations:** 1 Chair and Department of Chemical Technology of Drugs, Poznan University of Medical Sciences, Rokietnicka 3, 60-806 Poznań, Poland; 2 Doctoral School, Poznan University of Medical Sciences, Bukowska 70, 60-812 Poznań, Poland; 3 Chair and Department of Toxicology, Poznan University of Medical Sciences, Rokietnicka 3, 60-806 Poznań, Poland; 4 Department of Biotechnology and Cell Biology, Medical College, University of Information Technology and Management in Rzeszów, Sucharskiego 2, 35-225 Rzeszów, Poland; 5 Department of Pharmaceutical, Organic and Bioorganic Chemistry, Danylo Halytsky Lviv National Medical University, Pekarska 69, Lviv, 79010 Ukraine

**Keywords:** Anticancer activity, Bladder cancer cell line, Flavonoids, X-ray structure, Paal–Knorr synthesis, Pyrrole

## Abstract

Cancer remains one of the most significant health issues worldwide. By designing compounds with anticancer activity characterized by high selectivity towards cancer cells, medicinal chemistry focuses on the protection of healthy cells and tissues. In this study, we present the hybrid pharmacophore approach, which afforded a series of new pyrrole flavones. The synthetic strategy was based on the Paal-Knorr pyrrole synthesis, starting from aminoflavones through their condensation with 1,4-diketones and leading to 6- and 7-(pyrrol-1-yl) flavones. The isolated products underwent characterization using NMR and UV–VIS spectroscopy, mass spectrometry, TGA, DSC, and Microtox analyses. For all pyrrole flavones, single crystals were obtained and subjected to X-ray diffraction experiments. Their cytotoxic activity was assessed on two human bladder cancer cell lines (5637 and HT-1376) and one non-cancerous (MRC-5) cell line, showing the potential as anticancer agents. Flavone derivative with the 6-(2-methyl-5-phenylpyrrol-1-yl) moiety was active in the MTT assay towards 5637 and HT-1376 cancer cells after 24 h of incubation with IC_50_ values of 2.97 µM and 5.89 µM, respectively. Notably, flavone derivative with 7-(2-methyl-5-phenylpyrrol-1-yl) revealed cytotoxic activity towards 5637 and HT-1376 cells with IC_50_ values of 7.39 µM and 13.54 µM, respectively, without any effect on the viability of MRC-5 cells.

**Supplementary Information:**

The online version contains supplementary material available at 10.1038/s41598-025-91772-9.

## Introduction

Cancer is one of the most serious concerns when it comes to long-term quality of life. The current understanding of cancer development draws attention to several main aspects of its appearance, for example, deregulating cellular metabolism, evading growth suppressors, enabling replicative immortality, inducing or accessing vasculature, and activating invasion and metastasis^1,2^. Understanding the scale of the problem highlights the urgent need to counter this often fatal disease, the challenge being to gain more insights into the molecular basis of carcinogenesis and search for new therapeutic options.

When considering new treatment approaches, flavonoids constitute a particular class of active compounds of plant origin, which have an immense anti-cancer potential while showing incredible flexibility regarding chemical transformations. They are characterized by a common molecular structure, which includes two phenyl rings connected by a propane linker connected to one of them through an oxygen bridge, thus forming a flavan scaffold. As of today, the class has more than 8000 representatives, which, depending on the position of the substituents and the degree of saturation of the pyran ring, are divided into six main subclasses: flavones, flavanones, flavonols, flavanols, anthocyanidins, and isoflavones^3^. These compounds are widely known for their range of pharmacological activities on the human body, among which are: antioxidant, anti-ageing, anti-inflammatory, cytoprotective, and anti-cancer activity^4,5^. The bioactivities of flavonoids allow them to be considered in the alleviation of such conditions as cardiovascular diseases, Parkinson’s, Alzheimer’s diseases, ischemic stroke, malaria, diabetes, and others^5^. Their antitumor effect is manifested by, among others, activation of apoptotic proteins, production of reactive oxygen species, and inception of DNA damage^6^.

**Fig. 1:**
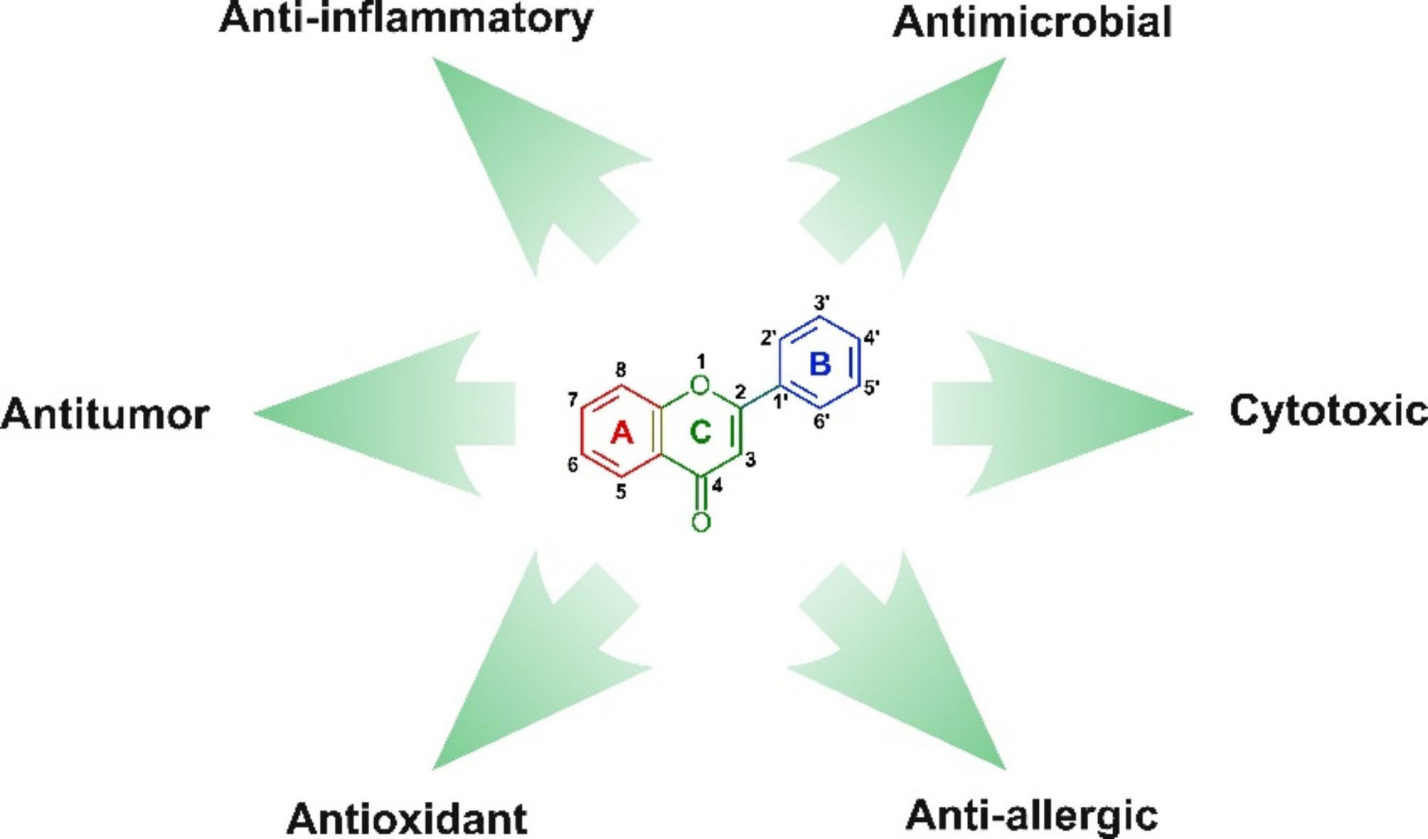
The flavone scaffold and pharmacological activities of flavone derivatives.

The flavone scaffold (Fig. 1) is based on a backbone of 2-phenylchromen-4-one (2-phenyl-1-benzopyran-4-one) and is widely present in plants^7^. Flavones act as pigments, UVB protectants, natural pesticides, and plant signaling molecules^8^. Numerous naturally occurring flavones are already recognized for their bioactive properties. These include apigenin (4′,5,7-trihydroxyflavone), luteolin (3′,4′,5,7-tetrahydroxyflavone), baicalein (5,6,7-trihydroxyflavone), nobiletin (3′,4′,5,6,7,8-hexamethoxyflavone), chrysin (5,7-dihydroxyflavone) and others^7^.

Peripherally modified flavones are the subject of vigorous study in medicinal chemistry, particularly due to the combination of the active flavone scaffold with other recognized pharmacophores. Different strategies have been proposed to improve the biological activity and physicochemical properties of flavones, including the following modifications: methylation^9,10^, alkylation^11^, and exchange of ketone to thioketone^12^. 6-Bromo-^13^, 6-hydroxy-^14^, 6,3′-dinitro-^15,16^, and 6-nitro-3′-bromoflavones^17^ showed distinct GABA_A_-modulating properties, whereas polyhydroxy- (baicalein, luteolin, apigenin) and polymethoxyflavones (nobiletin, tangeretin) revealed activity in the management of Parkinson’s disease^18^. Biflavones, like amentoflavone, ginkgetin, and isoginkgetin also presented activity in neurodegenerative diseases^19^. Another flavone, 4′,5,6-trihydroxylflavone-7-*O*-glucuronoside (scutellarin), showed significant inhibitory activity against human hepatocellular carcinoma^20^.

In the literature, several hybrids of flavones with different pharmacophores were presented, including 1,2,3-trizoles, amino acids, piperazines, and estradiol^21^. Separately, it is worth outlining the connections of flavones with heterocyclic moieties since such hybrids revealed various beneficial effects. For example, 6-imidazolidinonyl flavones showed anticancer and anti-inflammatory properties during in vitro and in vivo studies^22^, whereas 3′- and 4′-piperazinyl ones presented antioxidant potential^23^. Various *N*-heterocyclic derivatives were also investigated for anti-Alzheimer activity, which made it possible to note particularly active piperidine-, piperazine-, and morpholine-containing representatives^24^. Notably, active modulators of calcium channels were found among conjugates of 3-methoxyflavones and 1,4-dihydropyridines^25^.

Aminoflavones are considered derivatives of flavonoids with a C2–C3 double bond, an oxo group at the C4 position, and one or more amino groups. These compounds do not occur in nature but can be obtained by means of chemical synthesis^22^. Among the biological properties of aminoflavones, it is worth highlighting inhibition of α-glucosidase^26^, improvement of lipopolysaccharide-induced oxidative stress^27^, cytotoxicity towards murine leukemia L1210 cell line^28^, and antiproliferative effect on human breast cancer cell lines MCF-7 and T-47D^29,30^, while sparing healthy cells^31^.

**Fig. 2:**
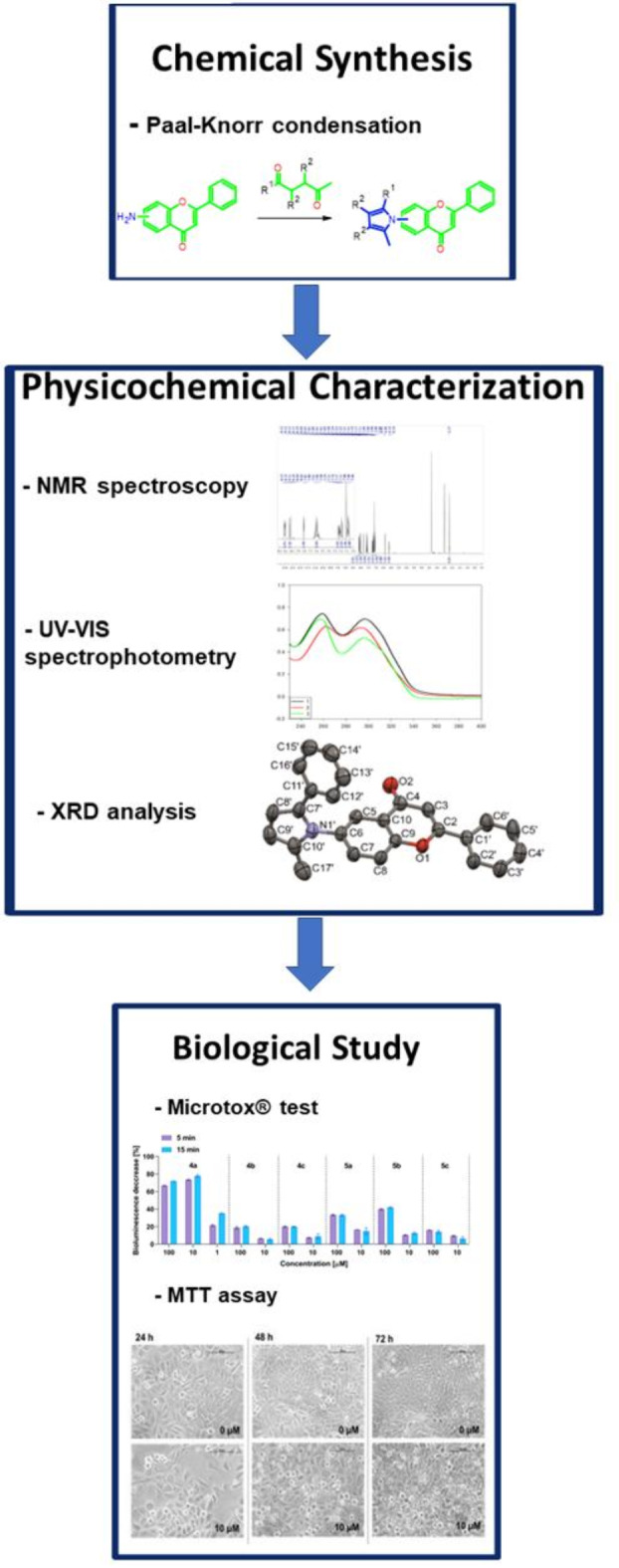
The general workflow applied in the current study.

In the scope of the present work, the main focus was the synthesis and physicochemical as well as biological analysis of pyrrole flavones. Since the key objective of the current project was to obtain flavone compounds with pyrrole substituents, various synthetic routes towards the desired molecules were studied. Among the available methods, one could consider the Hantzsch pyrrole synthesis due to the versatility of possible substrates^32^. Still, a limitation may be the need to fine-tune reaction parameters due to the potential risk of side product occurrence. Another prospective reaction, the Buchwald-Hartwig C-N cross-coupling guarantees good selectivity under mild conditions, but it requires expensive catalysts and a high level of expertise in their handling^33^. Ultimately, the Paal-Knorr condensation seemed optimal for synthesising an initial series of simple pyrrole flavones due to its straightforwardness, versatility and the availability of the corresponding diketones^34^. While simple pyrrole itself holds minimal significance in natural contexts, its derivatives serve as pivotal biorelevant compounds. Notably, the pyrrole ring features prominently in naturally occurring chemical compounds like porphyrins and synthetic pharmaceuticals such as ketorolac, sunitinib, atorvastatin, and ondansetron^35^. The literature data indicate that pyrrole-based compounds have proved effective against tyrosine kinases, microtubule polymerization, histone deacetylase, cytochrome P450, and bcl-2 proteins^36^. Although combinations of flavone with pyrrole moieties have been described previously^37–41^, in this study, we present the nature of such linkages from a medicinal chemistry perspective, indicating a straightforward one-step method for their preparation, as well as detailed physicochemical and biological characterization (Fig. 2).

## Results and discussion

### Chemical synthesis and characteristics

**Scheme 1:**
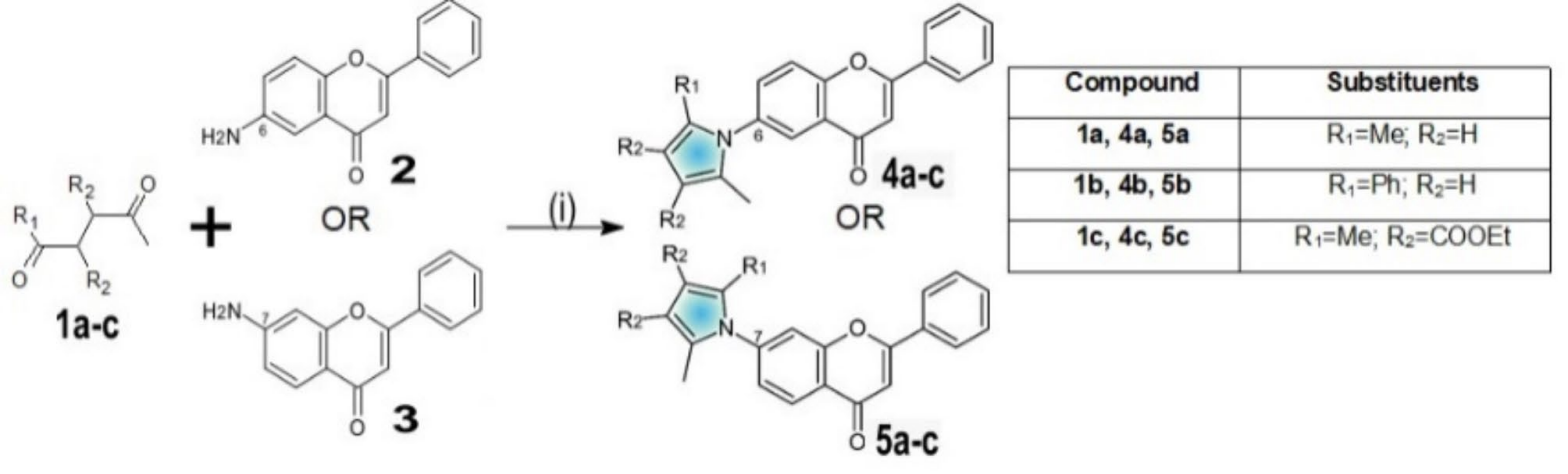
Reagents and conditions: (i) trifluoroacetic acid, methanol, reflux, 24h.

The aim of the synthetic part of the study was focused on obtaining novel pyrrole flavones of potential biological activity. To achieve the designated goal, the Paal-Knorr pyrrole condensation was applied for the modification of aminoflavones^34,42^. The substrate, either 6- or 7-aminoflavone, was treated with one of the selected 1,4-diketones—2,5-hexanedione, 1-phenyl-1,4-pentanedione, or diethyl 2,3-diacetylsuccinate in methanol as medium (Scheme 1). The reaction mixture was complemented by the catalytic amount of trifluoroacetic acid and refluxed overnight in the inert argon atmosphere. The desired products, in the form of pure pale-beige to pale-yellow crystals, were isolated by means of chromatographic methods.

A variety of analytical techniques, including ^1^H and ^13^C nuclear magnetic resonance (NMR) spectroscopy, electrospray ionization mass spectrometry (ESI–MS), and UV–VIS spectrophotometry (Figure S5), were utilized to confirm the structure of the obtained compounds **4a-c** and **5a-c** (please see Supplementary Information).

### Single crystal X-ray diffraction (SXRD), thermogravimetry (TGA) and differential scanning calorimetry (DSC)

Solids gained after synthesis were subjected to recrystallization experiments, which allowed single crystals suitable for X-ray diffraction for all compounds to be received. Crystals belong to the monoclinic crystal system to *P*2_1_/c (**4b**, **4c**, **5a**, **5c**) or *P*2_1_/n (**4a** and **5b**) space groups but with different unit cell parameters indicating various crystal packing (Table S1 and S2). The asymmetric unit of **4c** consists of two symmetry-independent diester molecules, A and B (Fig. S1).

Considering the relative orientation of ring moieties, the conformations of all compounds, except for **5c**, are comparable. The flavone part is nearly flat, and the pyrrole ring is significantly twisted against this unit (Table S3). In **5c**, the dihedral angle between benzopyran-4-one and phenyl rings is 39.73°. This twisting is uncommon for the compounds described here and over one hundred flavone derivatives from crystal structures deposited in CSD^43^, (Tables S3 and S4).

The most robust intermolecular contacts that might be formed in crystal structures are C-H···O hydrogen bonds, which are categorized as weak^44^. Crystal structure analysis for compounds incapable of forming strong hydrogen bonds might be enhanced by calculating Hirshfeld surfaces^45^ on which short contacts are assessed and quantified. The red areas on these surfaces plotted with d_norm_ properties represent contacts shorter than the sum of van der Waals radii of the atoms involved in the interaction.

**Fig. 3:**
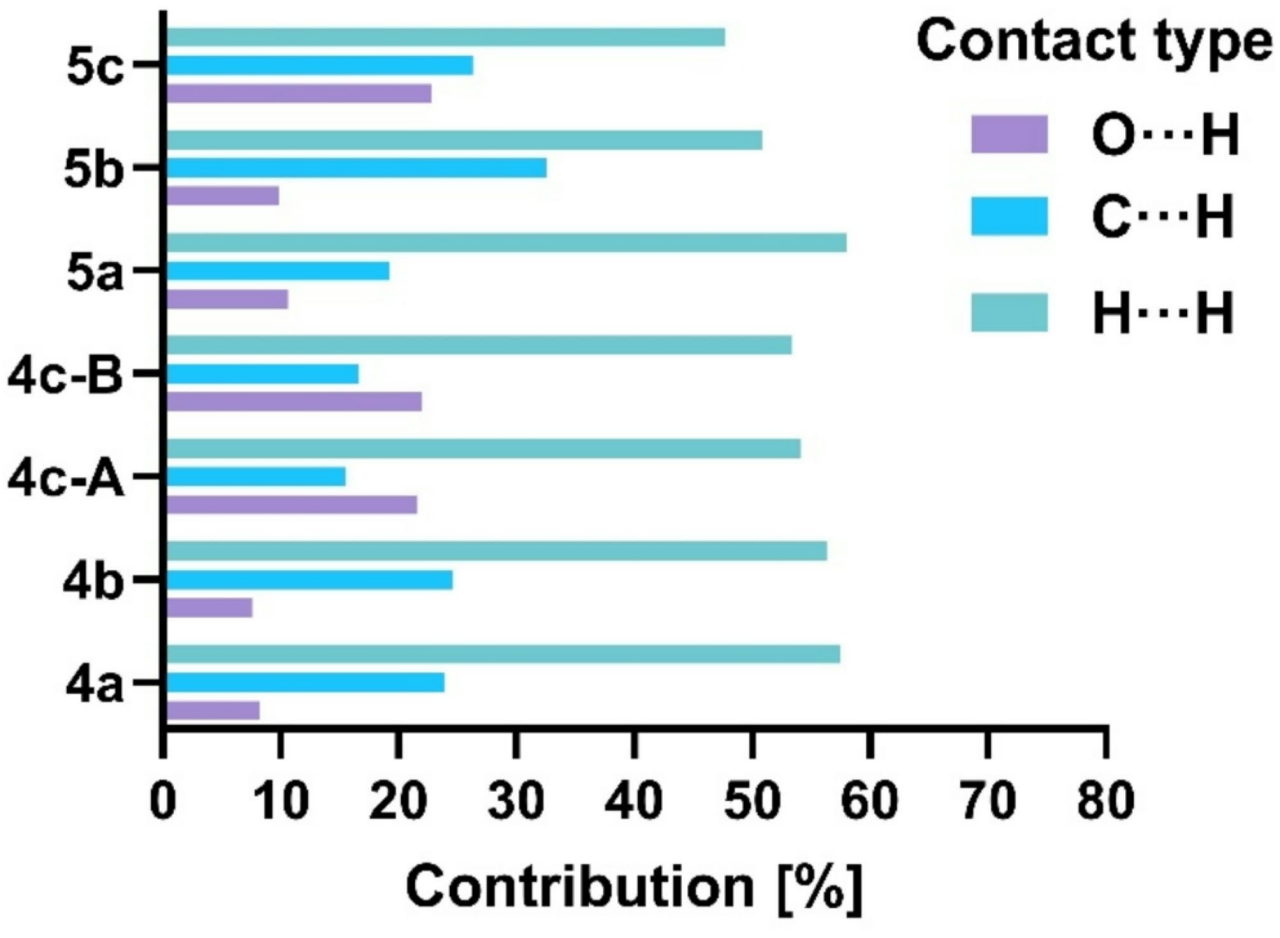
Percentage contribution of short contacts to the Hirshfeld surface area.

Calculations performed for the described compounds revealed the highest percentage contribution on the Hirshfled surface of H···H contacts in all crystals. Next, for the dimethyl and methyl-phenyl derivatives, the count of the C···H contacts prevail over O···H. A similar percentage contribution of these contacts is observed in **5c**, while for both symmetry-independent molecules in **4c**, O···H prevail over C···H (Fig. 3 and Table S5). This fluctuation is directly connected with the structural motifs formed, which differ in each crystal structure.

**Fig. 4:**
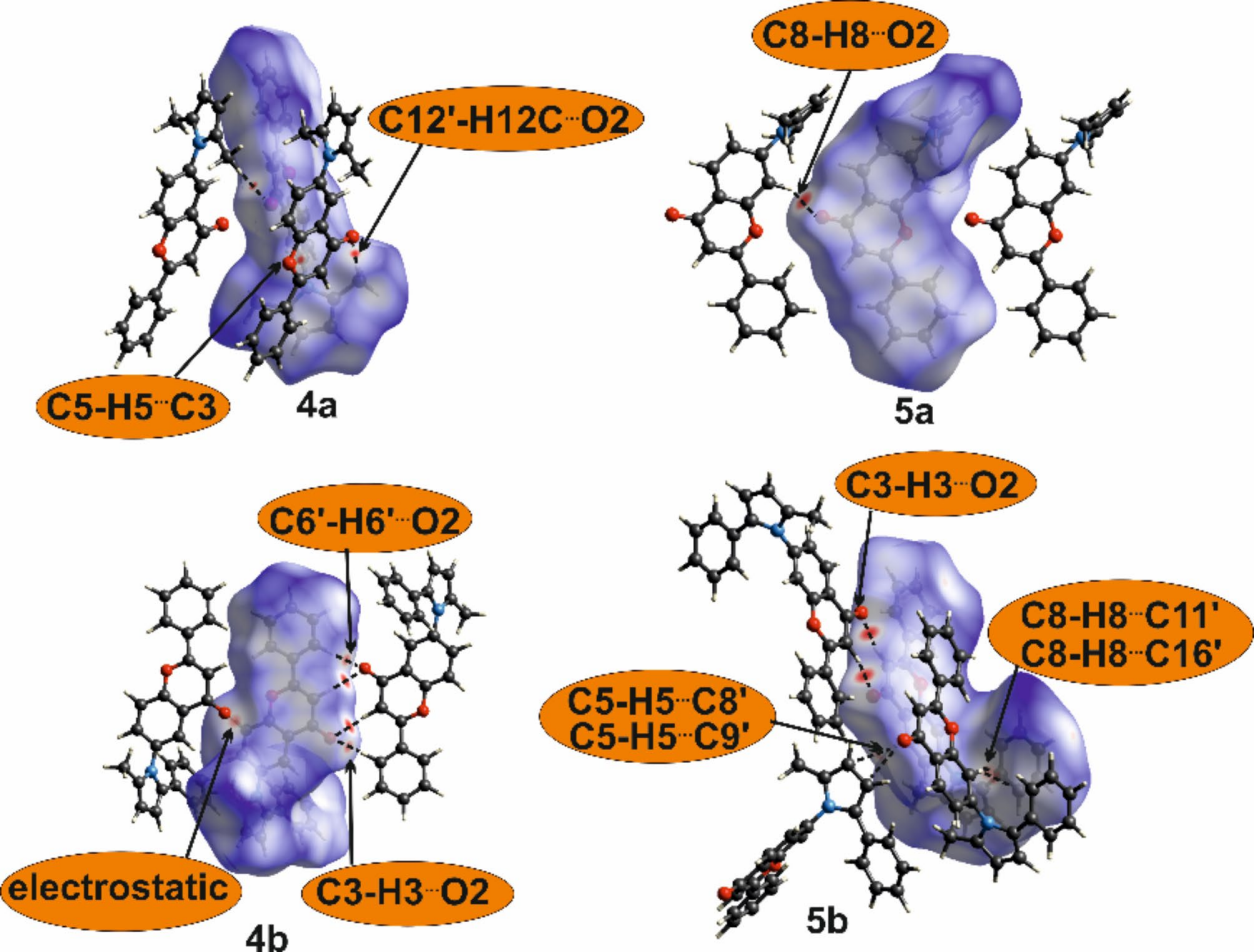
Molecules connected with short intermolecular contacts. For one molecule, Hirshfeld surfaces mapped with d_norm_ are shown. Symmetry codes are given in Table S6.

In **4a**, [010] branched chains are formed through short contact between the carbonyl (O2) and methyl (C12) groups (Fig. 4, Table S6). Adjacent chains are connected with C–H···π contacts between flavone phenyl and pyrrole rings (C7–H7···C10″ in Table S6), but on the Hirshfeld surfaces, this contact surface is not assigned in red.

**Fig. 5:**
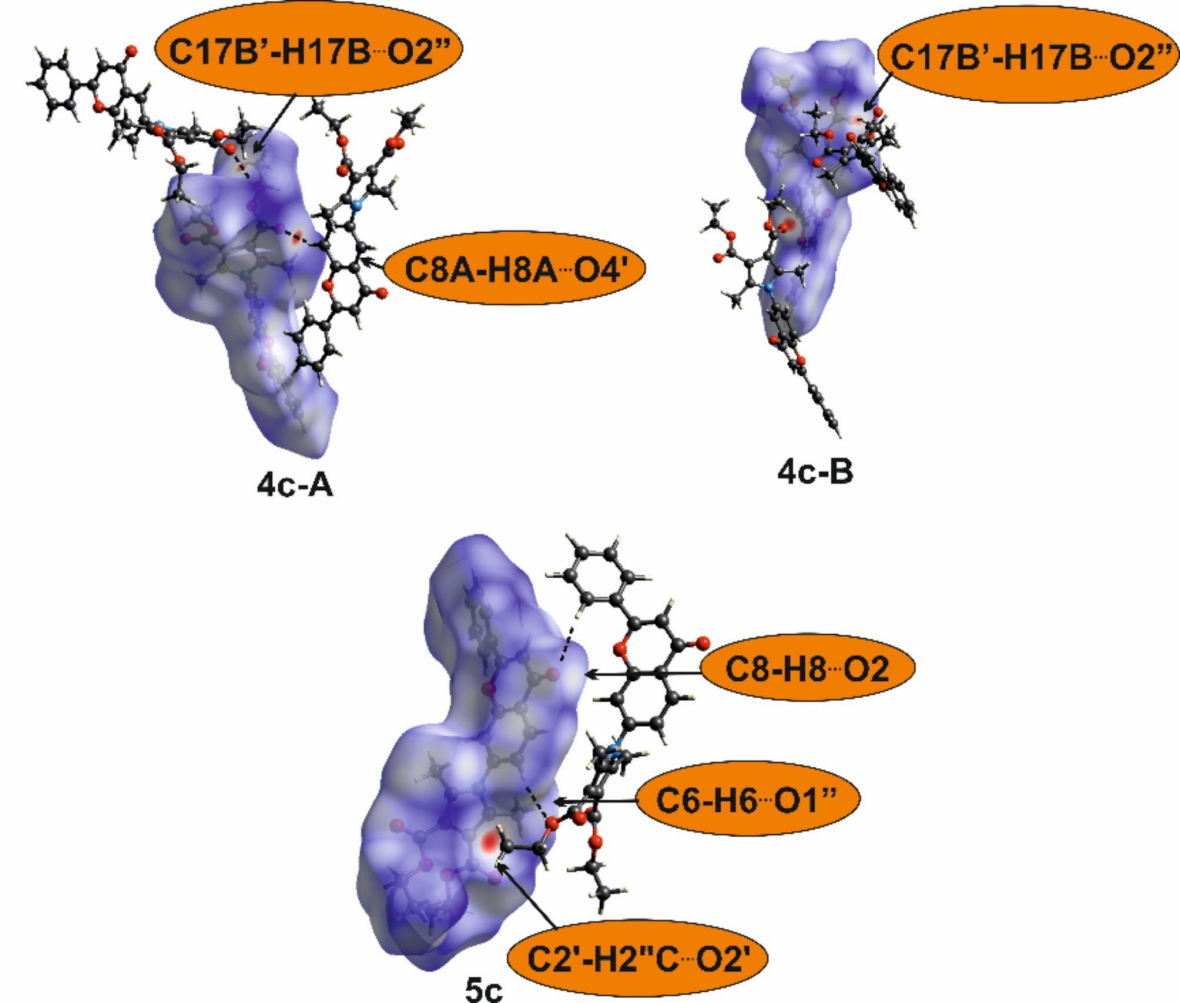
Molecules connected with short intermolecular contacts. For one molecule, Hirshfeld surfaces mapped with d_norm_ are shown. Geometrical parameters of short contact are given in Table S6.

The interaction of the carbonyl O2 atom with the aromatic C8-H group causes the formation of [001] chains in **5a** (Fig. 5, Table S6). In 3D structure, the overlay of the phenyl rings of flavone moiety is seen (Figure S4, but the geometrical parameters do not indicate π···π interactions (centroid···centroid, distances are longer than 4 Å). However, it should be noted that for this structure, the contribution of C···C contacts that might represent aromatic stacking to the Hirshfeld surface is the highest among all crystals (Fig. 4, Table S5).

In both crystals of methyl-phenyl derivatives, **4b** and **5b**, the primary structural motif is a centrosymmetric dimer based on C–H···O contact between carbonyl O2 and flavone C3–H atoms. In **4b**, the slight shift of the molecules allows the additional contact between carbonyl O2 and aromatic C6–H6 group within the dimer. Another short contact involving the carbonyl group revealed on the Hirshfeld surface is electrostatic interaction with the flavone C8–H8 group (Fig. 5). These two contacts arrange molecules into [100] chains. The overlay of the flavone phenyl and pyran-4-one rings allows the (010) layer to be distinguished (Figure S4).

The significantly higher percentage contribution of C···H contacts calculated for **5b** in comparison to other structures is connected with the number of C-H···π contacts in this crystal. They are formed between flavone benzene and pyrrole or phenyl ring attached to pyrrole (Fig. 4). In the Table S6 the geometry of these contacts is represented with C-H···C interactions.

Introducing additional carbonyl groups in the diester derivatives causes changes in the type of the shortest contacts.

In the **4c**, the flavone carbonyl group does not form contacts shorter than the sum of van der Waals radii with the C-H group. Instead, both symmetry-independent molecules A and B through C–H···O interactions of ester carbonyl and flavone C-H groups form two independent chains extending along [001] (Fig. 5). These chains are combined into (100) layers by contact between the carbonyl ester of molecule B and the CH_2_ group of molecule A (Table S6).

All three types of short C-H···O contacts represented with the negative values on the Hirshfeld surface, calculated for **5c,** are involved in the aggregation of the molecules into [001] chains (Fig. 5). They are formed between ester carbonyl O2′ and ester methyl C2′–H groups, ester alkoxyl O1′ (or O1′′) and aromatic flavone C6–H, and flavone carbonyl O2 and aromatic flavone C8–H (Table S6).

Crystal structures analysis completed with mapping of intermolecular contacts on the Hirshfeld surfaces demonstrated variability in structural motifs and molecular interactions not only between compounds with different substituents but also among isomers. Two interdependent factors might cause such diversity: (1) the inability to form strong intermolecular interactions and (2) overall molecular shape. Analyzed compounds can not interact through interactions considered as strong, e.g. O–H···O hydrogen bonds with energy *ca* 20–40 kJ/mol^44^. The observed C–H···O, C–H···π or stacking contacts usually have the energy of about 5 kJ/mol and lower^44^, thus not strong enough to be repeatable and definitive for crystal packing. Moreover, molecules have irregular and changeable shapes. To ensure the densest possible packing, the contact surfaces must change, causing the randomness of created intermolecular contacts and structural motifs.

The thermal properties of prepared powder samples were also characterized. TGA and DSC measurements were preceded by the powder X-ray diffraction (PXRD) experiments to ascertain the recrystallized samples’ phase composition. Registered diffractograms indicated their homogeneity and identity with phases observed in single crystals (Figs. S2 and S3). Thermogravimetric (TGA) analysis revealed that the thermal stability of the compounds was dependent on the type of the substituent (dimethyl, methyl-phenyl, or diester), not on the position (6 or 7) on the flavone core. The most stable were diester, and the least stable were dimethyl derivatives. It is impossible to show the trend for melting points determined using differential scanning calorimetry (DSC). However, no attempts were made to ascertain whether all obtained crystal structures are thermodynamically stable polymorphs (Figs. S2 and S3).

### Microtox analysis

All the synthesized derivatives were subjected to the Microtox test. Microtox is a rapid acute toxicity test that uses *Aliivibrio fischeri* bacteria^46^. *A. fischeri* is a bacterial species that shows bioluminescence (λ_max_ 490 nm), which decreases when the bacterial suspension is exposed to a toxic substance.

**Fig. 6:**
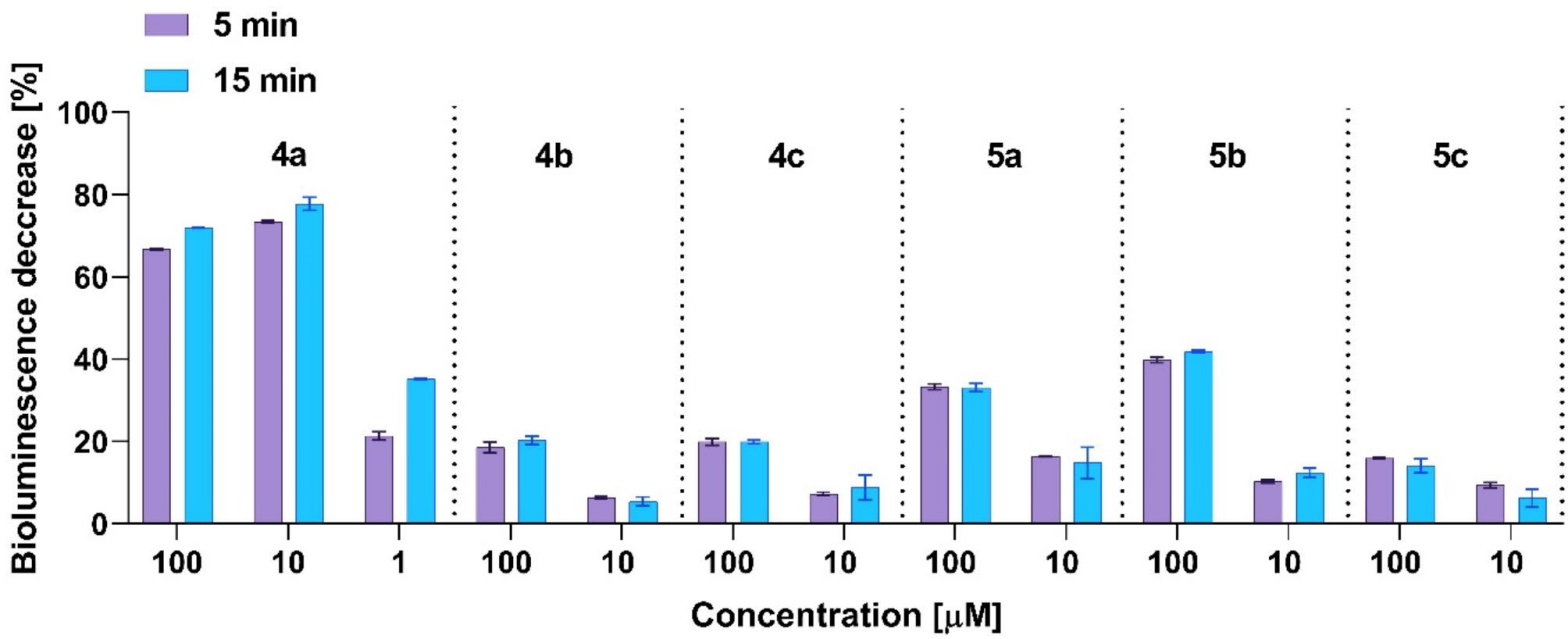
The decrease of *Aliivibrio fischeri* bioluminescence intensity upon exposition to the tested compounds **4a–c** and **5a–c** in different concentrations. Measurements were taken 5 and 15 min after adding of the pyrrolyl aminoflavones to the bacteria.

Due to the lack of water-solubility, aqueous solutions of compounds **4a**-**c** and **5a**-**c** were prepared using dimethylsulfoxide as a solubilizer, the addition of which was shown not to induce any toxic effect (< 1%). The results of the experiments are summarized in Fig. 6. It was found that the derivatives **4b**, **4c**, and **5a**-**c** exert similar effects, while compound **4a** stands out. This molecule may be regarded as the lead structure and could be further explored, as it shows greater effect in comparison to the 7-substituted isomer **5a**, and its 6-pyrrolyl analogs **4b** and **4c**.

The observed toxic effects may result from the tested compounds’ antibacterial action, as the test uses *A. fischeri*, which is a Gram-negative bacterium^47,48^. However, caution should be taken, as the tested bacteria are modified to improve the penetration into the bacterial cell for the purpose of rapid measurements.

### Cytotoxic effects of tested compounds

Natural compounds play a pivotal role in the development of anticancer therapies, with semisynthetic modifications significantly enhancing their clinical utility. Many natural compounds have been optimized for improved efficacy, selectivity, and reduced toxicity, making them indispensable in modern medicinal chemistry. For instance, epothilones, derived from myxobacteria, are a prominent example. These compounds stabilize microtubules similarly to paclitaxel but are more effective against taxane-resistant cancers^49^. The semisynthetic derivative ixabepilone has demonstrated significant clinical success in treating metastatic breast cancer^50^, while galactosylation of epothilone B has been reported as promising advancement in targeted anticancer therapies, addressing key limitations of natural epothilones, such as high toxicity and poor selectivity to tumor cells^51^. Podophyllotoxin is another cornerstone of anticancer drug development, utilizing plant-derived compounds. Its semisynthetic derivatives, such as etoposide and teniposide, are widely used in chemotherapy for their ability to inhibit topoisomerase II and disrupt cancer cell division^52^. Capsaicinoids, initially known for their analgesic effects, have been semisynthetically altered to enhance their anticancer activities, showing potential in targeting specific cancer pathways^53^. Additionally, glycopeptide antibiotics^54^, traditionally used as antimicrobial agents, have been chemically modified for anticancer applications due to their cytotoxic properties. To date, several flavonoid derivatives have been studied as potential therapeutic agents for breast, colon, and lung cancer cell lines and human lymphoma cells^7^. However, there is little information about the activity of such compounds on bladder cancer cells, thus the presented study can provide new insights into this topic. It should be highlighted that several naturally occurring flavones such as apigenin^55^, tangeretin^56^, baicalein, scutellarin^57^, nobiletin^58^, and orientin^59^ have already been tested using bladder cancer cell lines, and revealed anticancer activity through various mechanisms, including apoptosis induction, ferroptosis, cell cycle arrest, and reactive oxygen activation. However, information about synthetic flavone derivatives is limited.

**Fig. 7:**
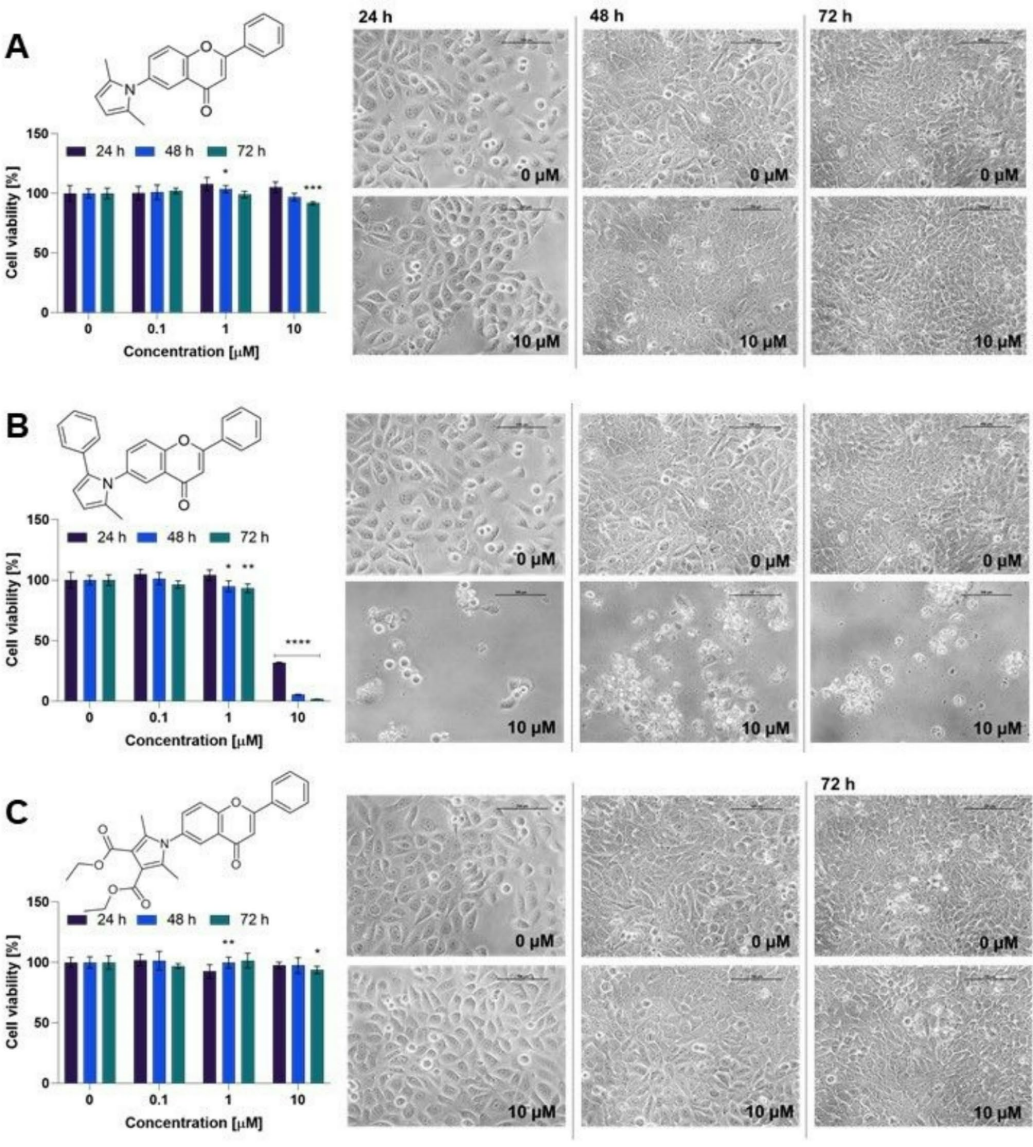
Results of the preliminary experiment for the 6-aminoflavone derivatives **4a–c**. Cell viability was determined using the MTT assay after incubation with compounds **4a** (**A**), **4b** (**B**), and **4c** (**C**) at a concentration of 0.1 µM, 1 µM, and 10 µM for 24, 48, and 72 h. A one-way ANOVA with post-hoc Dunnett’s test was assessed statistical significance, with *p* < 0.05 considered significant. Asterisks indicate statistical significance: *p < 0.05, ** *p* < 0.01, *** *p* < 0.001, and ****p < 0.0001 vs. the control group. The figure also shows representative images of 5637 cells morphology after incubation with **4a**–**c** at a concentration of 10 µM after 24, 48, and 72 h. The control cells were treated with DMSO at a concentration of 0.1% in a cell culture medium. The images were taken with a Nikon Eclipse TS100 microscope. The scale bar corresponds to 100 µm.

**Fig. 8:**
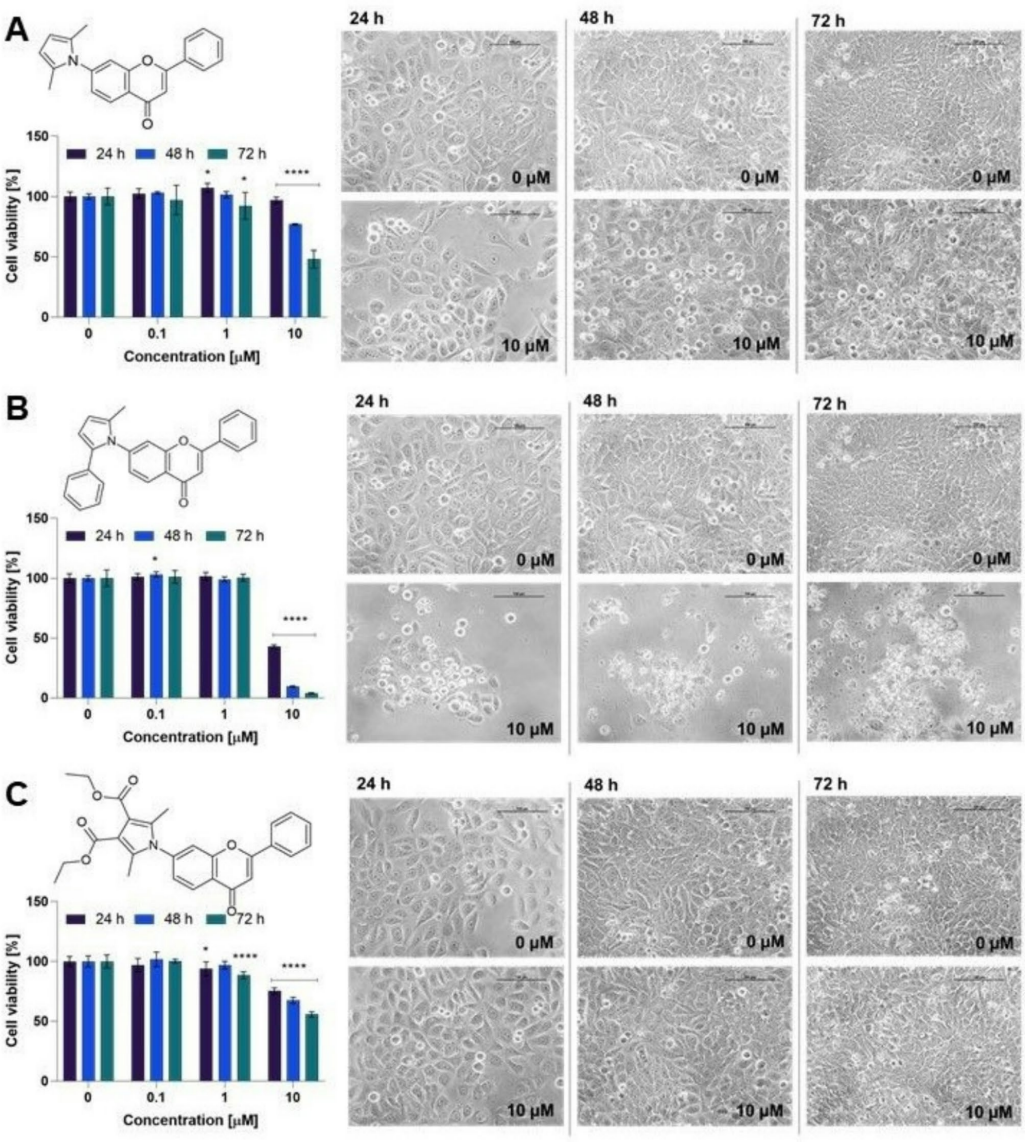
Results of the preliminary experiment for 7-aminoflavone derivatives **5a–c**. Cell viability after incubation with compounds **5a** (**A**), **5b** (**B**), and **5c** (**C**) at a concentration of 0.1 µM, 1 µM, and 10 µM for 24, 48, and 72 h was determined using the MTT assay. One-way ANOVA with post-hoc Dunnett’s test was used to determine significance, with *p* < 0.05 considered significant. Asterisks indicate statistical significance *p < 0.05, ****p < 0.0001 versus the control group. The figure shows the representative images of 5637 cells morphology after incubation with **5a**–**c** at a concentration of 10 µM after 24, 48, and 72 h. Control cells were treated with DMSO at a concentration of 0.1% in a cell culture medium. The images were taken with a Nikon Eclipse TS100 microscope. The scale bar corresponds to 100 µm.

In our study, all pyrrolyl flavones were studied towards 5637 cells using the high (10 µM), moderate (1 µM), and low (0.1 µM) concentrations. As presented in Figs. 7 and 8, two compounds exerted significant cytotoxic effects. Generally, the 2-methyl-5-phenylpyrrol-1-yl flavone derivatives (**4b** and **5b**) revealed the strongest cytotoxicity within the tested series.

Interestingly, compound **4a** decreased the bioluminescence of bacteria most effectively at a concentration of 10 µM, while it did not affect the viability of cancer cells at the same dose. On the other hand, the compound **5a** (an isomer of **4a**) reduced cell viability to 75.8% and 48% after 48 h and 72 h of incubation, respectively. Among the derivatives with a modification at position 6 of ring A, only compound **4a** demonstrated significant biological activity. Moreover, compound **4a** showed the greatest reduction in cell viability among all six compounds tested. In contrast, all compounds with a modification at position 7 of ring A affected cell viability, primarily after 72 h of incubation, with the most potent activity observed for compound **5b**. Furthermore, the substitution of the 6th or 7th position in ring A of the flavone scaffold by the 2-methyl-5-phenylpyrrol-1-yl group exerted a comparable effect on 5637 cell viability.

**Fig. 9:**
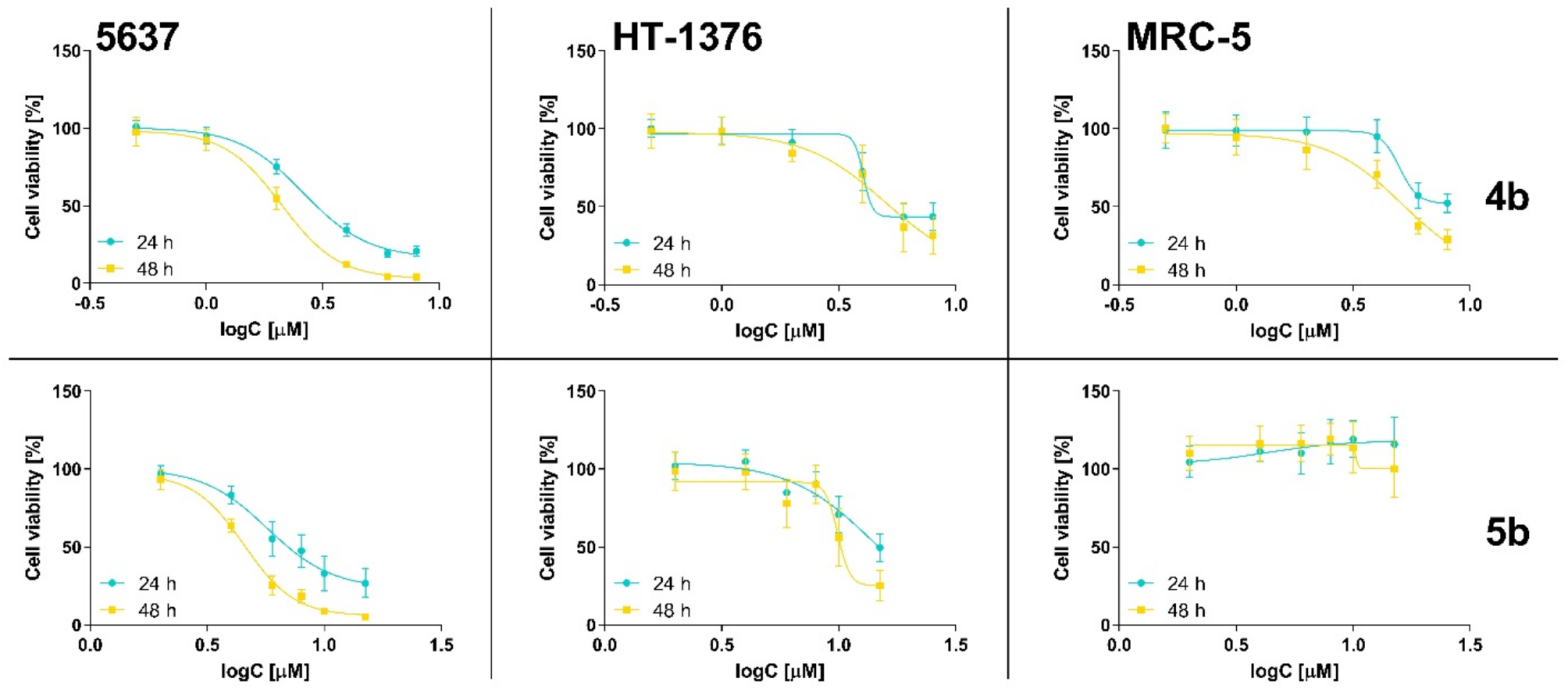
The dose–response curves for 5637, HT-1376, and MRC-5 cells after treatment with compounds **4b** and **5b** for 24 and 48 h. Data are presented as mean ± SD from at least three independent experiments.

Based on the preliminary results, compounds **4b** and **5b** were further analyzed to determine the IC_50_ values. Besides the 5637 cell line used in the preliminary results, both compounds were tested on another bladder cancer cell line, HT-1376. The cytotoxic effect was also evaluated on the non-cancerous human fibroblast MRC-5 cell line to ascertain the selectivity of the tested compounds. The dose–response curves for compounds **4b** and **5b** are presented in Fig. 9.

As presented in Table 1, compound **4b** exerted the strongest activity towards 5637 and HT-1376 cells. The IC_50_ values were approximately two times lower than those IC_50_ values obtained for compound **5b** at both incubation time points. However, after 48 h of incubation, compound **4b** decreased the viability of MRC-5 cells with IC_50_ values of 5.21 µM. Additionally, after 24 h of incubation, compound **4b** also affected the viability of MRC-5 cells, reducing cell viability to 57% and 52% for concentrations of 6 µM and 8 µM, respectively (Fig. 9). On the other hand, the compound **5b** did not decrease cell viability at all tested concentrations. Thus, it suggests that pyrrole moiety at position 7 ensures higher selectivity to non-cancerous cells. Moreover, increasing the incubation time did not change IC_50_ values for compound **4b**, while for compound **5b**, the treatment lasting 48 h decreased the cell viability of both cell lines more strongly.

**Table 1:** The IC_50_ values of compounds **4b** and **5b**.

	IC_50_ [µM]					
	5637		HT-1376		MRC-5	
	24 h	48 h	24 h	48 h	24 h	48 h
**4b**	2.97 ± 0.17	2.12 ± 0.19	5.89 ± 1.10	5.37 ± 1.44	> 8	5.21 ± 0.10
**5b**	7.39 ± 2.02	4.61 ± 0.10	13.54 ± 1.41	11.7 ± 1.74	> 15	> 15

The cytotoxic effect was studied against the 5637, HT-1376, and the MRC-5 cell lines. Data present the mean value ± SD from three independent experiments; only compound **5b** was tested towards 5637 in four biological replicates.

Furthermore, as shown in Fig. 8 and Table 1, compounds **4b** and **5b** exhibited stronger activity against 5637 cells. Both cell lines represent different histological subtypes of urothelial cell carcinoma: 5637 cells are non-muscle-invasive, while HT1376 cells represent muscle-invasive urothelial bladder cancer^60^. These subtypes differ in prognosis and treatment options, with muscle-invasive bladder cancer being associated with a higher risk of recurrence and a poorer prognosis^61^. Pinto-Leite and co-workers reported differences at the molecular level between 5637 and HT1376 cells^60^. Specifically, they identified *tumor suppressor protein 53* (*TP53*) deletions in both cell lines. HT1376 cells also exhibited a 10q deletion involving the *phosphatase and tensin homolog* (*PTEN*) region, with no alteration in the *phosphatidylinositol-4,5-bisphosphate 3-kinase catalytic subunit alpha* (*PIK3CA*) region, which contributes to the more invasive and metastatic properties of HT1376 cells compared to 5637 cells^60^. Thus, our study showed that the tested compounds are more active against less aggressive cancer cells; however, it should be highlighted that both compounds effectively decreased the viability of HT1376 cells, with IC_50_ values of approximately 5 µM for compound **4b** and approximately 13 µM for **5b** after 24 h of incubation.

As presented in Fig. 8, the results obtained for compound **5b**, including its selective activity against cancer cells, suggest that the mechanism of action may be related to the expression of receptors present on cancer cells or signaling pathways influencing the promotion of cancer cell proliferation and/or invasiveness. Literature data indicate that flavones’ anticancer activity may be related to protein tyrosine kinase inhibition, as observed for luteolin and chrysin^62^. Interestingly, Wang et al. showed that 5637 cells exhibit high expression of receptor d’Origine Nantais (RON), also known as the macrophage-stimulating protein (MSP) 1 receptor, which belongs to the receptor tyrosine kinase (RTK) family^63^. RON activation might trigger mitogen-activated protein kinase (MAPK), phosphatidylinositol 3-kinase (PI3K), and other signaling pathways, which can stimulate cancer cell proliferation, survival, migration, and invasion^63^. However, further studies, including cell cycle analysis, cell death modes such as apoptosis, necrosis, or autophagy, and screening analysis to find the potential molecular targets, are needed to fully understand the mechanism of action of the tested compounds.

## Conclusions

In the current work, the chemical synthesis of six new pyrrolyl flavones was described. All compounds were subjected to detailed physicochemical characterization and biological studies. Thermogravimetric analysis established a relationship between the type of pyrrolyl substituent attached to the A ring of the flavone core and the thermal stability of the whole molecule, which showed a decreasing tendency in the order diester > methyl-phenyl > dimethyl. X-ray diffraction analysis showed that crystal packing is governed by various intermolecular contacts, contributing to the formation of diverse structural motifs. It is not possible to distinguish recurring crystal structure building blocks.

In the Microtox test, the majority of compounds revealed low to moderate toxicity, which did not exceed a 40% decrease in the bioluminescence of the tested bacteria when using 100 μM concentrations for 15 min. However, flavone substituted with 2,5-dimethylpyrrolyl in the 6th position showed an 80% decrease in bioluminescence when using even a 10 μM solution. The cytotoxic properties of newly developed synthetic flavones were tested on bladder cancer cell lines 5637 and HT-1376, as well as on the MRC-5 fibroblast line. Among the tested compounds, 2-methyl-5-phenylpyrrolyl flavone derivatives substituted in the 6th and 7th positions showed the most pronounced cytotoxic effects, with 6-substituted regioisomer being the superior against cancer cells (IC_50_ after 48 h was 2.12 ± 0.19 μM on line 5637 and 5.37 ± 1.44 μM on line HT-1376), however, at the same time, it decreased the viability of MRC-5 cells. On the other hand, the 7-substituted regioisomer exhibited comparatively weaker cytotoxic properties (IC_50_ after 48 h was 4.61 ± 0.10 µM on line 5637 and 11.7 ± 1.74 µM on line HT-1376), yet, it demonstrated selectivity towards non-cancerous cells.

The present study constitutes an initial approach towards the synthesis of modified aminoflavones via Paal-Knorr condensation, along with investigating their physicochemical nature and evaluating their biological properties. Interestingly, the efficacy of aminoflavones as potential active pharmaceutical ingredients was proved against bladder cancer cells. The authors believe that the obtained data will draw attention to the chemistry of synthetic flavonoids, deepen the understanding of the properties of heterocyclic systems, and encourage further discoveries in the field of biologically active hybrid compounds. The two most active aminoflavones were functionalized in the 6th or the 7th position with a 2-methyl-5-phenylpyrrolyl group. The results of the study presented here prompted us to continue the aminoflavone modification with both halogenated pyrroles as well as phenyl-functionalized pyrrolyl systems. The subsequent series of structurally related molecules have been subjected to physicochemical and biological studies and will be presented soon.

## Materials and methods

### General experimental details

All solvents and reagents were obtained from commercial suppliers and used without further purification. Melting points (m.p.) were obtained on a “Stuart” Bibby SMP 10 apparatus and are uncorrected. The NMR spectra (^1^H NMR, ^13^C NMR, ^1^H-^1^H COSY, ^1^H-^13^C HSQC, and ^1^H-^13^C HMBC) were recorded using a full-fledged, two-channel 400 MHz Bruker AvanceCore NMR spectrometer. All spectra were recorded at 25 °C. Chemical shifts (δ) are quoted in parts per million (ppm) and are referred to as residual solvent peaks. Coupling constants (*J*) are quoted in Hertz (Hz). The abbreviations s, d, dd, t, q and m refer to singlet, doublet, doublet of doublets, triplet, quintet, and multiplet, respectively. Thin layer chromatography (TLC) was performed on silica gel Merck Kieselgel 60 F_254_ plates and DC Kieselgel 60 RP-18 F_254_ and visualized with UV illumination (λ_max_ 254 or 365 nm). UV–VIS spectra were recorded on a Jasco UV–VIS V-770 spectrophotometer; λ_max_ (logε), nm. Mass spectra (ESI^+^) were recorded at the Center for Advanced Technology, Adam Mickiewicz University.

### General synthetic procedures

#### General procedure for the synthesis of pyrrole flavones

Corresponding aminoflavone (237 mg, 1 mmol) was dissolved in methanol (10 mL) in a round bottom flask (25 mL). Then, diketone derivative (1.1 mmol) and trifluoroacetic acid (catalytic amount) were added to the reaction mixture. The resulting solution was stirred overnight at 70 °C in an inert gas (Ar) environment. After cooling the reaction mixture to room temperature, a precipitate was formed, and the solvent was evaporated to give pale-yellow solids, which were later purified using flash column chromatography (with silica gel as the stationary phase and *n*-hexane/EtOAc 7:3 as a mobile phase). Details for individual procedures and characteristics of products are described below.

##### *6-(2,5-Dimethylpyrrol-1-yl)-2-phenylchromen-4-one* (**4a**)

The following reagents were applied: 6-aminoflavone (1 mmol, 237 mg), 2,5-hexanedione (1.1 mmol, 129 µL) and trifluoroacetic acid (catalytic amount, ~ 3 drops). Compound **4a** was obtained as a pale-yellow solid (205 mg, 65% yield): m.p. 168–169 °C; R_*f*_ 0.535 (DCM/MeOH 50:1); UV–VIS (CH_3_OH): λ_max_, nm (logε) 259.2 (4.36), 296.8 (4.33). ^1^H NMR (400 MHz, DMSO-*d*_6_) δ 8.18 – 8.14 (m, 2H), 7.96 (dd, *J* = 8.6, 0.6 Hz, 1H), 7.79 (d, *J* = 2.4 Hz, 1H), 7.76 (dd, *J* = 8.6, 2.6 Hz, 1H), 7.66 – 7.58 (m, 3H), 7.14 (s, 1H), 5.85 (s, 2H), 2.00 (s, 6H); ^13^C NMR (101 MHz, DMSO-*d*_6_) δ 177.2, 163.5, 155.1, 136.0, 134.6, 132.5, 131.5, 129.7, 128.2, 127.0, 124.2, 123.7, 120.5, 107.3, 106.9, 13.3. HRMS ESI (pos) *m/z* calcd (C_21_H_18_NO_2_): 316.1332 [M + H]^+^, found 316.1332.

##### *6-(2-Methyl-5-phenylpyrrol-1-yl)-2-phenylchromen-4-one* (**4b**)

The following reagents were applied: 6-aminoflavone (1 mmol, 237 mg), 1-phenyl-1,4-pentanedione (1.1 mmol, 176 µL) and trifluoroacetic acid (catalytic amount, ~ 3 drops). Compound **4b** was obtained as a pale-yellow solid (245 mg, 65% yield): m.p. 172–173 °C; R_*f*_ 0.581 (DCM/MeOH 50:1); UV–VIS (CH_3_OH): λ_max_, nm (logε) 262.2 (4.36), 293.2 (4.35). ^1^H NMR (400 MHz, DMSO-*d*_6_) δ 8.16 – 8.10 (m, 2H), 7.89 (dd, *J* = 8.6, 0.7 Hz, 1H), 7.73 – 7.71 (m, 1H), 7.69 (dd, *J* = 8.6, 2.7 Hz, 1H), 7.65 – 7.57 (m, 3H), 7.20 – 7.13 (m, 2H), 7.11 – 7.07 (m, 2H), 7.07 – 7.01 (m, 2H), 6.37 (d, *J* = 3.5 Hz, 1H), 6.11 (dd, *J* = 3.5, 1.0 Hz, 1H), 2.10 (d, *J* = 1.0 Hz, 3H); ^13^C NMR (101 MHz, DMSO-*d*_6_) δ 177.1, 163.4, 154.9, 136.6, 134.8, 134.0, 133.3, 132.5, 132.0, 131.4, 129.6, 128.8, 127.8, 127.0, 126.3, 124.1, 124.0, 120.4, 109.8, 108.6, 107.3, 13.5. HRMS ESI (pos) *m/z* calcd (C_26_H_20_NO_2_): 378.1489 [M + H]^+^, found 378.1480.

##### *Diethyl 2,5-dimethyl-1-(4-oxo-2-phenylchromen-6-yl)pyrrole-3,4-dicarboxylate* (**4c**)

The following reagents were applied: 6-aminoflavone (1 mmol, 237 mg), diethyl 2,3-diacetylsuccinate (1.1 mmol, 284 mg) and trifluoroacetic acid (catalytic amount, ~ 3 drops). Compound **4c** was obtained as a pale-yellow solid (138 mg, 30% yield): m.p. 147–148 °C; R_*f*_ 0.186 (DCM/MeOH 50:1); UV–VIS (CH_3_OH): λ_max_, nm (logε) 257.4 (4.50), 296.6 (4.38), 310.2 (4.29). ^1^H NMR (400 MHz, DMSO-*d*_6_) δ 8.17 (dd, *J* = 8.0, 1.7 Hz, 2H), 8.03 (d, *J* = 8.9 Hz, 1H), 7.95 (d, *J* = 2.5 Hz, 1H), 7.87 (dd, *J* = 8.8, 2.6 Hz, 1H), 7.66 – 7.59 (m, 3H), 7.16 (s, 1H), 4.19 (q, *J* = 7.1 Hz, 4H), 2.09 (s, 6H), 1.25 (t, *J* = 7.1 Hz, 6H); ^13^C NMR (101 MHz, DMSO-*d*_6_) δ 177.0, 165.1, 163.5, 155.9, 134.6, 134.2, 133.7, 132.6, 131.4, 129.7, 127.0, 124.8, 124.5, 121.1, 112.9, 107.5, 60.1, 14.6, 12.0. HRMS ESI (pos) *m/z* calcd (C_27_H_25_NO_6_Na): 482.1580 [M + Na]^+^, found 482.1584.

##### *7-(2,5-Dimethylpyrrol-1-yl)-2-phenylchromen-4-one* (**5a**)

The following reagents were applied: 7-aminoflavone (1 mmol, 237 mg), 2,5-hexanedione (1.1 mmol, 129 µL) and trifluoroacetic acid (catalytic amount, ~ 3 drops). Compound **5a** was obtained as a pale-yellow solid (205 mg, 65% yield): m.p. 187–188 °C; R_*f*_ 0.465 (DCM/MeOH 50:1); UV–VIS (CH_3_OH): λ_max_, nm (logε) 250.4 (4.28), 307.4 (4.37). ^1^H NMR (400 MHz, DMSO-*d*_6_) δ 8.18 – 8.13 (m, 3H), 7.83 (d, *J* = 1.8 Hz, 1H), 7.66 – 7.56 (m, 3H), 7.41 (dd, *J* = 8.4, 2.0 Hz, 1H), 7.13 (s, 1H), 5.88 (s, 2H), 2.07 (s, 6H); ^13^C NMR (101 MHz, DMSO-*d*_6_) δ 177.1, 163.4, 156.4, 143.7, 132.4, 131.4, 129.6, 128.3, 126.9, 126.2, 125.9, 122.8, 118.2, 107.6, 107.5, 13.4. HRMS ESI (pos) *m/z* calcd (C_21_H_18_NO_2_): 316.1332 [M + H]^+^, found 316.1345.

##### *7-(2-Methyl-5-phenylpyrrol-1-yl)-2-phenylchromen-4-one* (**5b**)

The following reagents were applied: 7-aminoflavone (1 mmol, 237 mg), 1-phenyl-1,4-pentanedione (1.1 mmol, 176 µL) and trifluoroacetic acid (catalytic amount, ~ 3 drops). Compound **5b** was obtained as a pale-yellow solid (245 mg, 65% yield): m.p. 173–174 °C; R_*f*_ 0.581 (DCM/MeOH 50:1); UV–VIS (CH_3_OH): λ_max_, nm (logε) 258 (4.31), 299.6 (4.37). ^1^H NMR: (400 MHz, DMSO-*d*_6_): δ 8.13–8.10 (m, 2H), 8.04–8.02 (dd, J = 8.0 Hz, 2H), 7.81–7.80 (d, J = 7.8 Hz, 1H), 7.62–7.56 (m, 3H), 7.24-0.7.21 (m, 1H), 7.21–7.17 (m, 2H), 7.11 (s, 1H), 7.09–7.06 (m, 3H), 6.41–6.40 (d, J = 6.4 Hz, 1H), 6.15–6.14 (dd, J = 3.5, 1.0 Hz, 1H), 2.17 (s, 3H); ^13^C NMR (101 MHz, DMSO-*d*_6_): δ 177.1, 163.4, 156.3, 144.3, 133.9, 133.2, 132.5, 132.1, 131.3, 129.6, 128.8, 127.7, 126.9, 126.5, 126.4, 126.2, 122.6, 118.5, 110.3, 109.1, 107.7, 13.6. ^13^C NMR (101 MHz, DMSO-*d*_6_): δ 177.1, 163.4, 156.3, 144.3, 133.9, 133.2, 132.5, 132.1, 131.3, 129.6, 128.8, 127.7, 126.9, 126.5, 126.4, 126.2, 122.6, 118.5, 110.3, 109.1, 107.7, 13.6. HRMS ESI (pos) *m/z* calcd (C_26_H_20_NO_2_): 378.1489 [M + H]^+^, found 378.1505.

##### *Diethyl 2,5-dimethyl-1-(4-oxo-2-phenylchromen-7-yl)pyrrole-3,4-dicarboxylate* (**5c**)

The following reagents were applied: 7-aminoflavone (1 mmol, 237 mg), diethyl 2,3-diacetylsuccinate (1.1 mmol, 284 mg) and trifluoroacetic acid (catalytic amount, ~ 3 drops). Compound **5c** was obtained as a pale-yellow solid (138 mg, 30% yield): m.p. 191–192 °C; R_*f*_ 0.186 (DCM/MeOH 50:1); UV–VIS (CH_3_OH): λ_max_, nm (logε) 254.4 (4.42), 297.4 (4.37), 311.8 (4.30). ^1^H NMR (400 MHz, DMSO-*d*_6_) δ 8.20 (d, *J* = 8.4 Hz, 1H), 8.16 – 8.12 (m, 2H), 8.02 (d, *J* = 1.8 Hz, 1H), 7.66 – 7.58 (m, 3H), 7.52 (dd, *J* = 8.4, 1.9 Hz, 1H), 7.17 (s, 1H), 4.19 (q, *J* = 7.1 Hz, 4H), 2.15 (s, 6H), 1.25 (t, *J* = 7.1 Hz, 6H); ^13^C NMR (101 MHz, DMSO-*d*_6_) δ 177.0, 165.0, 163.6, 156.4, 141.2, 133.9, 132.6, 131.3, 129.6, 126.9, 126.8, 126.1, 124.0, 119.4, 113.2, 107.8, 60.2, 14.6, 12.0. HRMS ESI (pos) *m/z* calcd (C_27_H_25_NO_6_Na): 482.1580 [M + Na]^+^, found 482.1572.

### Solid state characterization

#### Single crystal X-ray diffraction (SXRD)

Single crystals used for X-ray diffraction experiments were obtained by slow evaporation of their solutions in DCM/methanol mixture. All measurements were conducted at room temperature with an Oxford Diffraction SuperNova diffractometer equipped with a hi-flux micro-focus Nova CuKα radiation source. Data collection and reduction were performed with CrysAlis PRO software^64^. The Olex2 1.3^65^ and programs implemented therein were used to solve, refine and finish crystal models. The structures were solved using a dual-space algorithm with the ShelXT^66^ structure solution program and refined with the ShelXL^67^ refinement package using Least Squares minimization. In **5c**, one ester group is refined as disordered over two positions. This model used the DFIX and SADI instructions to restrain bond lengths and atomic displacement parameters. All of the non-H atoms were refined anisotropically. Hydrogen atoms were placed in idealized positions and were refined as riding on their carriers with Uiso(H) = 1.2Ueq (CH) and Uiso(H) = 1.5Ueq(CH_3_).

Details on structure refinements are presented in Tables S1 and S2 The geometry of short contacts is given in Table S6. The numbering of atoms is shown in Figure S1.

#### Cambridge structural database (CSD) analysis

Surveying the Cambridge Structural Database^43^ with flavone’s unsubstituted B and C rings and allowing any substitution of ring A as an entry revealed over 117 crystal structures of flavon derivatives, including flavone itself and different cocrystals. Restriction of this result to 6- or 7-substituted flavone core returned only 15 entries. None of the structures were derivatives of aminoflavone.

#### Hirshfeld surfaces calculations

The Hirshfeld surfaces and fingerprint plots were calculated using *CrystalExplorer21*, Version 21.5 software^45^. Surfaces were computed with a high (standard) resolution with the d_norm_ property plotted. The blue regions correspond to contacts longer than van der Waals radii, with a positive d_norm_ value. White areas correspond to the distance of contacts exactly equal to the van der Waals separation with a d_norm_ value of zero. Red spots correspond to distances shorter than van der Waals radii, with a negative d_norm_ value.

#### Powder X-ray diffraction experiments (PXRD)

Powder X-ray data were collected on a Bruker AXS D2 Phaser diffractometer (Bruker, Germany) with Cu Kα radiation. Before X-ray diffraction experiments, samples were ground with a mortar and pestle. Steel holders with 25 mm diameter and 1 mm depth were used for measurements. Scans were collected with a 2θ range between 5–35°, a step size of 0.02°, a counting rate of 2 s/step, and operating conditions of 30 kV and 10 mA. The samples were spinning at 30 rpm. A 1 mm slit module was used during measurements.

#### Differential scanning calorimetry (DSC)

Differential scanning calorimetry thermograms were collected with DSC 214 Polyma (NETZSCH, Selb, Germany). Samples were closed in aluminum pans with the pierced cover. The nitrogen atmosphere (30 mL/min) was maintained during measurements. The scans were made with a heating rate of 5 K/min.

#### Thermogravimetry (TGA)

Thermogravimetric analysis was performed using a TG 209 F3 Tarsus instrument (NETZSCH, Selb, Germany). The open corundum crucible was used for measurements. The analyzed samples were heated at a 5 K/min rate under a nitrogen atmosphere (30 mL/min).

### Biological study

#### Evaluation of acute toxicity using the Microtox test

The acute toxicity of the tested compounds was measured using the Microtox test. For the measurements, the 81.9% screening test procedure was applied using the Microtox M500 apparatus along with the Microtox Omni 4.2 software supplied by the manufacturer (Modern Water plc). Briefly, the bioluminescence of bacterial suspension was measured before the addition of the solution of the tested compound and after 5 and 15 min upon exposure. For the measurements, 10 mM stock solutions of the compounds **4a**-**c** and **5a**-**c** were prepared in DMSO and were diluted with deionized water to appropriate concentrations. As the control, 2% sodium chloride solution was used (Modern Water Microtox Diluent).

#### Cytotoxic activity

##### Cell cultures

Reagents used for in vitro experiments, such as Dulbecco’s modified Eagle’s medium (DMEM), fetal bovine serum (FBS), penicillin–streptomycin-L-glutamine solution, Dulbecco’s phosphate-buffered saline (DPBS), trypsin–EDTA, dimethyl sulfoxide (DMSO), 3-(4, 5-dimethylthiazol-2-yl)-2,5-diphenyltetrazolium bromide (MTT) were purchased from Sigma Aldrich (St. Louis, MO, USA). Roswell Park Memorial Institute 1640 (RPMI) medium, Eagle’s Minimum Essential Medium (EMEM) were obtained from Gibco, Thermofisher Scientific (Waltham, MA, USA). The DMSO for MTT assay was obtained from Avantor Performance Materials (Gliwice, Poland).

Human urinary bladder grade II carcinoma cell line (5637), human urinary bladder carcinoma (HT-1376), and human lung fibroblasts (MRC-5) were purchased from ATCC (Manassas, VA, USA). The 5637 cells were grown in RPMI, HT-1376 cells were cultured in EMEM, and MRC-5 in DMEM. All media were supplemented with 10% (*v*/*v*) FBS, 2 mM L-glutamine, 100 U/mL penicillin, and 10 mg/mL streptomycin (1% *v*/*v*). The cells were cultured at 37 °C in a humidified atmosphere with 5% CO_2_.

Tested compounds were dissolved in DMSO at a concentration of 10 mM for preliminary study. In contrast, for determining the IC_50_, compound **4a** was dissolved at a concentration of 8 mM, and compound **5b** to 15 mM. Stock solutions were stored in the dark at -20 °C.

##### MTT assay

For the preliminary experiments, the 5637 cells were used. The compounds were prepared in the cell culture medium at a concentration of 0.1 µM, 1 µM, and 10 µM. DMSO was used as a control. The concentration in cell culture medium did not exceed 0.1%. Cells were treated with compounds for 24, 48, and 72 h. Cells were washed twice with DPBS, and 170 µL of MTT solution at a final concentration of 0.59 mg/mL in culture medium was added. Cells were incubated with MTT solution for 1.5 h at standard cell culture conditions. After incubation, MTT was removed, and formazan crystals were dissolved with 200 µL DMSO. The absorbance was measured at a wavelength of 570 nm using a plate reader (Biotek Instruments, Elx-800, Winooski, VT, USA).

To determine the IC_50_ values, 5637, HT-1376, and MRC-5 cells were seeded at a density of 15 × 10^3^ cells per well in 96-well plates and incubated overnight at standard cell culture conditions. Subsequently, cells were treated with compound **4a** at a concentration of 0.5 µM, 1 µM, 2 µM, 4 µM, 6 µM, and 8 µM and compound **5b** at a concentration range of 2 µM, 4 µM, 6 µM, 8 µM,10 µM, and 15 µM for 24 h and 48 h. DMSO was used as a control, with a concentration in the cell culture medium did not exceeding 0.1%. The MTT assay was performed as described above.

The IC_50_ values were calculated using the GraphPad Prism 8 software (GraphPad Software, Inc., La Jolla, CA, USA), and cell viability was expressed as a percentage of the control. At least three biological replicates were performed for each compound.

#### Statistical analysis

The statistical analysis was performed using GraphPad Prism8 (GraphPad Software, Inc., La Jolla, CA, USA). One-way ANOVA with post-hoc Dunnett’s test was used to determine the significance; p < 0.05 was considered as significant.

## Electronic supplementary material

Below is the link to the electronic supplementary material.

Supplementary Material 1

## Data Availability

All data generated or analysed during this study are included in this published article [and its supplementary information files].
