# Supplementary material for: Synthesis, physicochemical characterization and biological activity of novel pyrrole flavones
Source: Sci Rep. 2025 Mar 3;15:7385. doi: 10.1038/s41598-025-91772-9 (PMC11876692; doi:10.1038/s41598-025-91772-9)
Supplement: Supplementary file 1 — Supplementary Material 1 [file 41598_2025_91772_MOESM1_ESM.docx]

**Synthesis, Physicochemical Characterization and Biological Activity of Novel** **Pyrrole Flavones**

Stepan Sysak^a,b^, Barbara Wicher^a^, Malgorzata Kucinska^c^, Paulina Kobylka^b,c^, Dariusz T. Mlynarczyk^a^, Roman Lesyk^d,e^, Ewa Tykarska^a^, Marek Murias^c^, Tomasz Goslinski^a,*^ Wojciech Szczolko^a,*^

^a^ Chair and Department of Chemical Technology of Drugs, Poznan University of Medical Sciences, Rokietnicka 3, 60-806 Poznań, Poland;

^b^ Doctoral School, Poznan University of Medical Sciences, Bukowska 70, 60-812 Poznań, Poland;

^c^ Chair and Department of Toxicology, Poznan University of Medical Sciences, Poznan University of Medical Sciences, Rokietnicka 3, 60-806 Poznań, Poland;

^d^ Department of Biotechnology and Cell Biology, Medical College, University of Information Technology and Management in Rzeszów, Sucharskiego 2, 35-225 Rzeszow, Poland;

^e^ Department of Pharmaceutical, Organic and Bioorganic Chemistry, Danylo Halytsky Lviv National Medical University, Pekarska 69, 79010 Lviv, Ukraine

* Correspondence: wszczolko@ump.edu.pl (WS), tomasz.goslinski@ump.edu.pl (TG)

# **Supplementary Information**

# **Characterization of the solid-state…………………………………………...............S2**

**NMR data of 4a-5c.....………….………………………………….……………...…S10**

**UV-VIS spectra of 4a-c and 5a-c……………………………………………………S27**

# **Characterization of the solid-state**

**Table S1.** Crystal data and refinement details for **4a**, **4b** and **4c**.

| **Identification code** | **4a** | **4b** | **4c** |
| --- | --- | --- | --- |
| **Chemical formula** | C_21_H_17_NO_2_ | C_26_H_19_NO_2_ | C_27_H_25_NO_6_ |
| **Formula weight** | 315.36 | 377.42 | 459.48 |
| **Crystal system** | Monoclinic | Monoclinic | Monoclinic |
| **Space group** | *P*2_1_/n | *P*2_1_/c | *P*2_1_/c |
| **a, b, c (Å)** | 14.2591 (6),  7.1953 (2),  16.5955 (5) | 6.0238 (2),  38.492 (1),  8.4012 (3) | 34.098 (5),  7.8911 (8),  18.876 (2) |
| **β** (°) | 99.396 (3) | 96.099 (4) | 105.447 (15) |
| **Volume (Å^3^)** | 1679.8 (1) | 1936.95 (12) | 4895.4 (11) |
| ***Z*** | 4 | 4 | 8 |
| **Density (calculated) (Mg m^-3^)** | 1.247 | 1.294 | 1.247 |
| **Absorption coefficient (mm^-1^)** | 0.64 | 0.65 | 0.73 |
| **Crystal size (mm)** | 0.40 × 0.30 × 0.05 | 0.50 × 0.07 × 0.07 | 0.20 × 0.15 × 0.02 |
| **Reflections collected** | 14024 | 12963 | 38192 |
| **Reflections observed**  **[*I* > 2σ(*I*)]** | 2571 | 2859 | 5350 |
| ***R*_int_** | 0.022 | 0.46 | 0.73 |
| **Data/parameters/restrains** | 3425/219/ 0 | 3898/263/0 | 8991/622/2 |
| **Goodness-of-fit on F^2^** | 1.03 | 1.03 | 1.03 |
| **Final R indices [*I* > 2σ(*I*)]** | 0.0462, 0.1274 | 0.0537, 0.1404 | 0.0708, 0.1988 |
| **R indices (all data)** | 0.0606, 0.1445 | 0.0705, 0.1585 | 0.1096, 0.2361 |
| **Largest diff. peak and hole** | 0.13, -0.14 | 0.15, -0.22 | 0.30, -0.25 |
| **CCDC #** | 2394582 | 2394581 | 2394584 |

**Table S2.** Crystal data and refinement details for **5a**, **5b** and **5c**.

| **Identification code** | **5a** | **5b** | **5c** |
| --- | --- | --- | --- |
| **Chemical formula** | C_21_H_17_NO_2_ | C_26_H_19_NO_2_ | C_27_H_25_NO_6_ |
| **Formula weight** | 315.36 | 377.42 | 459.48 |
| **Crystal system** | Monoclinic | Monoclinic | Monoclinic |
| **Space group** | *P*2_1_/c | *P*2_1_/n | *P*2_1_/c |
| **a, b, c (Å)** | 8.0088 (3), 30.6676 (9), 7.3600 (3) | 5.5474 (2),  25.5876 (7), 13.7806 (3) | 16.5661 (4), 11.5687 (2), 12.6365 (3) |
| **β** (°) | 113.626 (5) | 94.054 (2) | 105.319 (3) |
| **Volume (Å^3^)** | 1656.18 (12) | 1951.19 (10) | 2335.72 (10) |
| ***Z*** | 4 | 4 | 4 |
| **Density (calculated) (Mg m^-3^)** | 1.265 | 1.285 | 1.307 |
| **Absorption coefficient (mm^-1^)** | 0.65 | 0.64 | 0.73 |
| **Crystal size (mm)** | 0.40 × 0.25 × 0.05 | 0.30 × 0.15 × 0.07 | 0.30 × 0.20 × 0.03 |
| **Reflections collected** | 17097 | 16064 | 19505 |
| **Reflections observed**  **[*I* > 2σ(*I*)]** | 2785 | 3108 | 4006 |
| ***R*_int_** | 0.035 | 0.32 | 0.732 |
| **Data/parameters/restrains** | 3425/219/ 0 | 3976/263/0 | 4782/340/12 |
| **Goodness-of-fit on F^2^** | 1.02 | 1.02 | 1.05 |
| **Final R indices [*I* > 2σ(*I*)]** | 0.0428, 0.1105 | 0.0430, 0.1122 | 0.0589, 0.1687 |
| **R indices (all data)** | 0.0510, 0.1192 | 0.0562, 0.1260 | 0.0671, 0.1805 |
| **Largest diff. peak and hole** | 0.12, -0.16 | 0.15, -0.20 | 0.37, -0.36 |
| **CCDC #** | 2394583 | 2394585 | 2394586 |


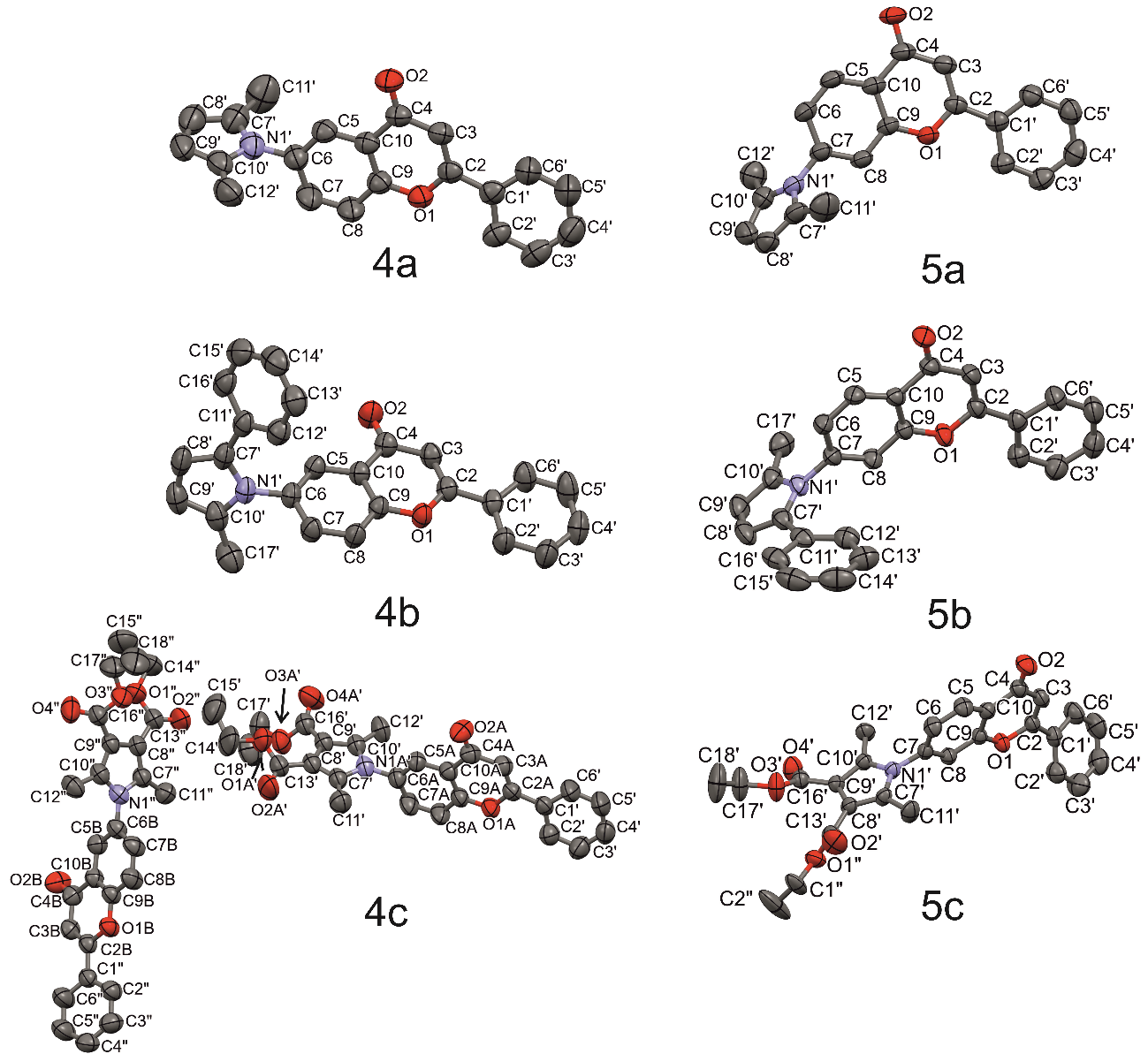


**Figure S1.** ORTEP representation of asymmetric units. Displacement ellipsoids are shown at the 50% probability level. H atoms have been omitted for clarity. For disordered ester moiety in **5c**, only the major position is shown.


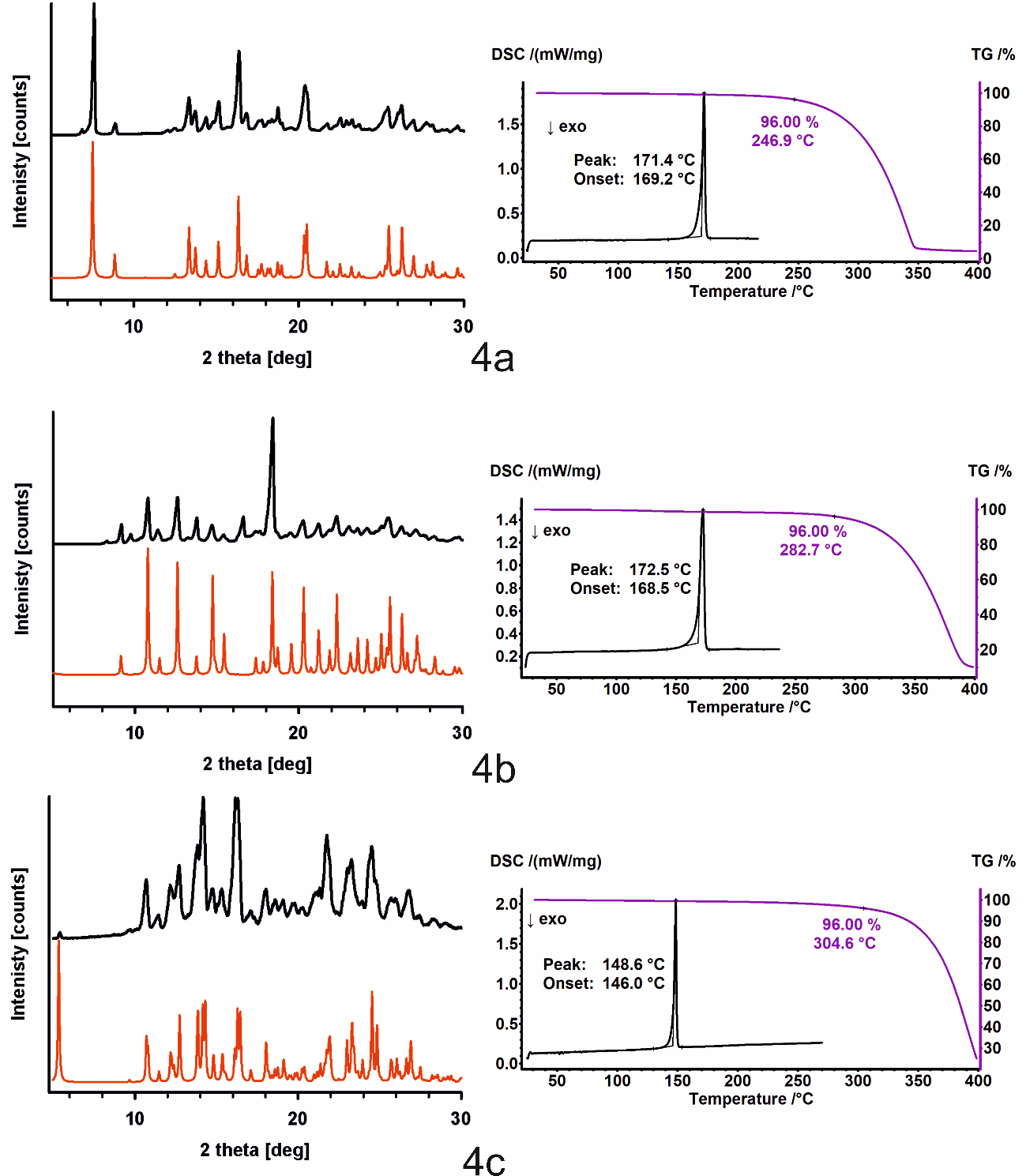


**Figure S2**. Powder X-ray diffractograms and DSC (black) and TGA (purple) thermograms of **4a**, **4b** and **4c**. Red diffractograms are simulated based on crystal structures, and black are registered for the recrystallized samples.


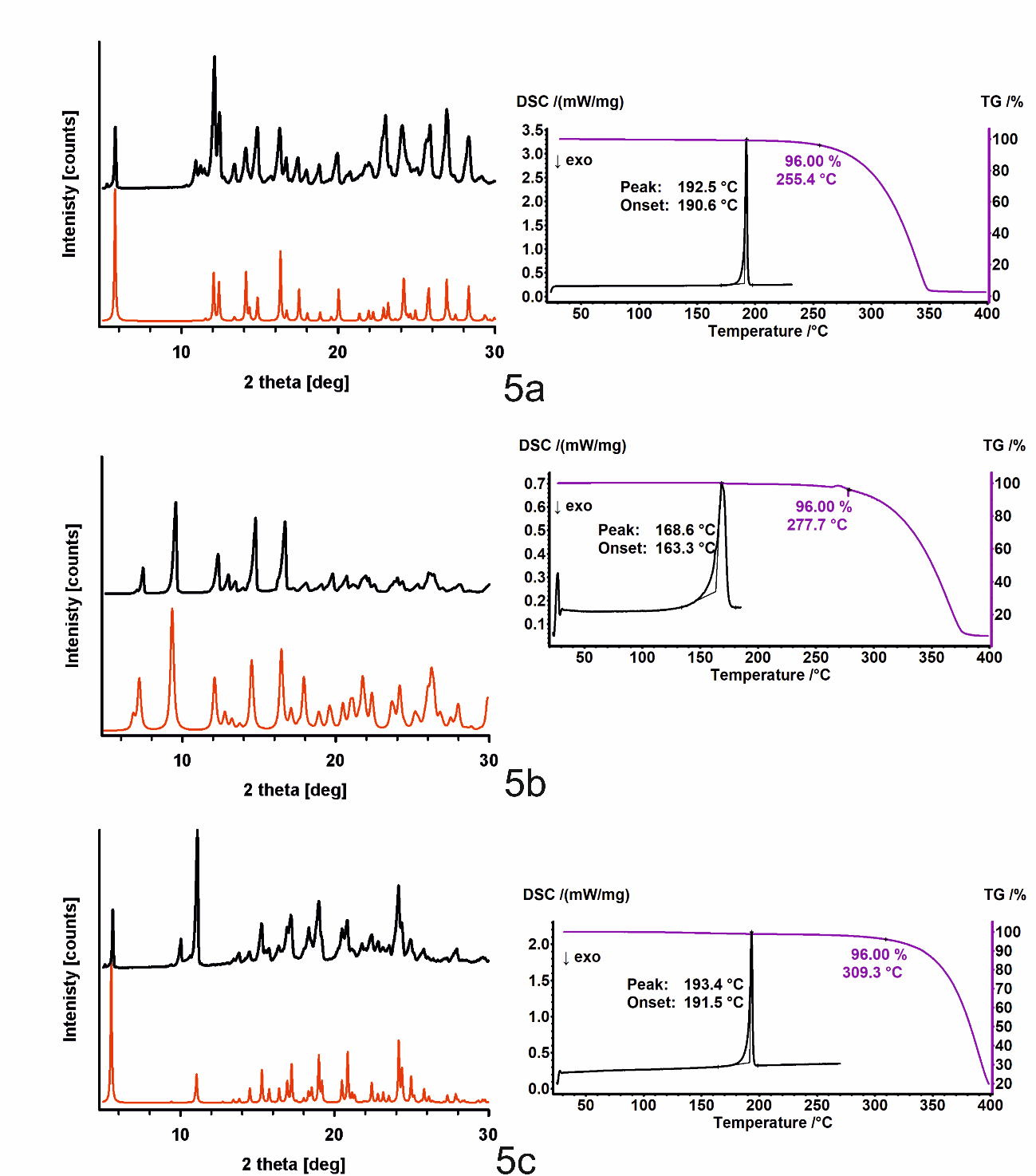


**Figure S3**. Powder X-ray diffractograms and DSC (black) and TGA (purple) thermograms of **5a**, **5b** and **5c**. Red diffractograms are simulated based on crystal structures, and black are registered for the recrystallized samples.

**Table S3.** Dihedral angles (°) between different ring moieties observed in the solid state.

|  | Benzopyran-4-one - phenyl | Benzopyran-4-one- pyrrole | Pyrrolyl - phenyl |
| --- | --- | --- | --- |
| **4a** | 7.30 (6) | 76.92 (7) |  |
| **4b** | 9.56 (8) | 71.00 (7) | 30.48 (9) |
| **4c-A** | 10.11 (13) | 88.32 (12) |  |
| **4c-B** | 15.65 (13) | 88.52 (10) |  |
| **5a** | 10.66 (4) | 66.59 (6) |  |
| **5b** | 1.30 (5) | 81.68 (6) | 28.52 (7) |
| **5c** | 39.73 (8) | 75.20 (7) |  |

**Table S4.** Dihedral angles (°) between benzopyran-4-one and phenyl rings observed for flavone moieties in the crystal structures deposited in the Cambridge Structural Database.

| **REFCODE** | **angle** | **REFCODE** | **angle** | **REFCODE** | **angle** | **REFCODE** | **angle** |
| --- | --- | --- | --- | --- | --- | --- | --- |
| IYIWOE | 54.99 | JICKIS | 13.83 | GOPZET | 7.90 | IDAYUI | 2.97 |
| OQOGIN | 41.18 | BALREN | 13.76 | ZUNHIB | 7.82 | SOLPUF | 2.93 |
| OQOGIN | 40.88 | ALAJUS | 12.59 | JAYNAZ | 7.56 | VICBIV | 2.91 |
| IYIWEU | 37.29 | HECHAY02 | 12.38 | PEHROK | 7.54 | HURCAY | 2.84 |
| MIXVIZ | 34.71 | HECHAY | 12.23 | ALAJOM | 7.30 | GUZSIF | 2.79 |
| NOCTIL01 | 33.40 | EJEQOB | 12.15 | QOLLAF | 7.25 | KASXIM | 2.77 |
| LEPBOZ | 31.85 | QARGEX | 12.07 | VUXZAQ | 7.15 | BIYQAE | 2.31 |
| EGURUU01 | 31.01 | GUZSEB | 12.02 | VOJDUU | 6.82 | PUZLII | 2.23 |
| LEPBOZ | 30.99 | HECHAY03 | 11.91 | RAMGOB02 | 6.80 | AFOCUV | 1.90 |
| EGURUU | 30.86 | PUZKUT | 11.85 | POMDUR | 6.70 | EMEWID | 1.82 |
| LEPBIT | 29.91 | LOHDAQ | 11.77 | KUNFAA | 6.69 | ZOGDEH | 1.79 |
| TOHZIC | 29.62 | EJEQAN | 11.64 | QOLMUA | 6.15 | LIXMEN | 1.61 |
| LEPBIT | 29.18 | NIXLOW | 11.47 | PIMLAB | 6.15 | NEFQOF | 1.48 |
| XOYVOY | 29.01 | HECHAY01 | 11.13 | KAMQOH | 6.11 | NEFQOF01 | 1.38 |
| LEPBUF | 28.31 | RUHDOP | 10.87 | POMPAK | 6.04 | WADRAV | 1.34 |
| JICKIS | 26.77 | KUNFEE | 10.81 | GUZRIE | 5.91 | KAMQUN | 1.20 |
| LEPBUF | 26.29 | HIQWUZ | 10.67 | KAMQIB | 5.75 |  |  |
| KAMJUD | 24.79 | EGURUU01 | 10.58 | KEJBAC | 5.68 |  |  |
| KAMJUD01 | 24.77 | CAHYIW | 10.57 | YAYQOF | 5.65 |  |  |
| BAZGOB | 24.75 | CAHYIW01 | 10.46 | POMDUR | 5.45 |  |  |
| XAGKOF | 21.69 | WADRAV | 10.43 | KAMQOH | 5.44 |  |  |
| ZUNHOH | 21.21 | EGURUU | 10.36 | KUPLOZ | 5.42 |  |  |
| UQEHAA | 21.18 | GUZREA | 10.34 | WAPMIK | 5.03 |  |  |
| GAZWUB | 19.99 | VUCFUU01 | 10.08 | LUFTIR | 4.84 |  |  |
| VOHKEK | 19.96 | GUZRUQ | 10.05 | YULFIV | 4.53 |  |  |
| LAMDIM | 19.03 | GUZSAX | 9.86 | NOCTIL | 4.45 |  |  |
| GOPZET | 18.39 | ZUNHOH01 | 9.80 | LOWQOG | 4.43 |  |  |
| RUHDOP | 18.29 | SULFUB | 9.77 | ETATOL | 4.40 |  |  |
| IYIWIY | 18.25 | VUCFUU | 9.73 | RUHDOP | 4.31 |  |  |
| SOLPUF01 | 17.07 | RAMGOB01 | 9.72 | LOWQOG | 4.19 |  |  |
| OWIDIJ | 16.69 | YEJVEO | 9.67 | KAMREY | 4.04 |  |  |
| VOHKEK01 | 16.48 | RUHDOP | 9.64 | JIJWIL | 4.04 |  |  |
| TOLQOD | 16.38 | MUJPAL | 9.50 | EWIVEN | 3.96 |  |  |
| OQIWIX | 16.02 | RAMGOB02 | 9.06 | LAWTOV | 3.85 |  |  |
| JICKIS | 15.92 | UMAXEO | 8.90 | LUFTIR | 3.79 |  |  |
| BUTWAQ | 15.52 | KAMRAU | 8.86 | SULFOV | 3.76 |  |  |
| GUZRAW | 14.71 | TALGIX | 8.72 | EGUXUA | 3.70 |  |  |
| QARGEX | 14.06 | EDEJUS | 8.70 | AMEXIB | 3.68 |  |  |
| TUKGIS | 13.94 | RAMGOB | 8.60 | SULFOV | 3.43 |  |  |
| ISATAZ | 13.94 | YULFIV | 8.35 | JILSIJ | 3.23 |  |  |
| MURRUO | 13.91 | GUZROK | 8.03 | GUZSEB | 3.04 |  |  |

**Table S5** Percentage contribution of short contacts to the Hirshfeld surface area.

|  | **H···H** | **C···H** | **O···H** | **C···C** | **O···C** | **N···H** | **O···O** | **N···C** |
| --- | --- | --- | --- | --- | --- | --- | --- | --- |
| **4A** | 57.5 | 23.9 | 8.2 | 3.8 | 6.1 | 0.6 | 0 | 0 |
| **5A** | 58.0 | 19.2 | 10.6 | 8.3 | 2.9 | 0.9 | 0 | 0 |
| **4B** | 56.3 | 24.6 | 7.6 | 6.5 | 4 | 0.5 | 0.3 | 0.1 |
| **5B** | 50.8 | 32.5 | 9.8 | 3.8 | 2.3 | 0.5 | 0 | 0.2 |
| **4C-A** | 54.1 | 15.5 | 21.6 | 4.5 | 3.9 | 0.3 | 0 | 0 |
| **4C-B** | 53.4 | 16.6 | 22.0 | 3.9 | 1.8 | 0.3 | 0 | 0 |
| **5C** | 47.7 | 26.3 | 22.8 | 0.3 | 1.7 | 0.6 | 0.7 | 0 |

**Table S6** Geometry (Å,°) of short contacts observed in crystal structures.

| *D*—H···*A* | *D*—H | H···*A* | *D*···*A* | *D*—H···*A* |
| --- | --- | --- | --- | --- |
| ***4a*** |  |  |  |  |
| C12’-H12C···O2^i^ | 0.96 | 2.66 | 3.615 (3) | 176 |
| C5-H5···C3^i^ | 0.93 | 2.83 | 3.560 (2) | 136 |
| C7-H7···C10’^xi^ | 0.93 | 2.90 | 3.703 (2) | 145 |
| **5a** |  |  |  |  |
| C8-H8···O2^iv^ | 0.93 | 2.45 | 3.311 (2) | 154 |
| **4b** |  |  |  |  |
| C3-H3···O2^iii^ | 0.93 | 2.54 | 3.456 (2) | 170 |
| C6’-H6’···O2^iii^ | 0.93 | 2.60 | 3.457 (3) | 153 |
| **5b** |  |  |  |  |
| C3-H3···O2^v^ | 0.93 | 2.47 | 3.348 (2) | 158 |
| C5-H5···C8’^vi^ | 0.93 | 2.87 | 3.681 (2) | 147 |
| C5-H5···C9’^vi^ | 0.93 | 2.78 | 3.592 (2) | 147 |
| C8-H8···C11’^vii^ | 0.93 | 2.82 | 3.721 (2) | 164 |
| C8-H8···C16’^vii^ | 0.93 | 2.81 | 3.625 (2) | 147 |
| **4c** |  |  |  |  |
| C8A-H8A···O4A’^ix^ | 0.93 | 2.52 | 3.417 (4) | 162 |
| C8B-H8B···O4”^viii^ | 0.93 | 2.46 | 3.348 (4) | 157 |
| C17’-H17B···O2” | 0.97 | 2.53 | 3.336 (5) | 140 |
| **5c** |  |  |  |  |
| C6-H6···O1”^x^ | 0.93 | 2.49 | 3.391 (7) | 164 |
| C6-H6···O1’^x^ | 0.93 | 2.78 | 3.625 (7) | 156 |
| C2’-H2”C···O2’^ix^ | 0.96 | 2.71 | 3.493 (9) | 139 |
| C8-H8···O2^ix^ | 0.93 | 2.61 | 3.230 (2) | 124 |

Symmetry codes: (i) 1.5-X, 0.5+Y, 1.5-Z; (ii) -0.5+X, 0.5-Y, -0.5+Z; (iii) -X, 0.5+Y, 0.5-Z, (iv) X, Y, -1+Z; (v) 1-X, 1-Y, 2-Z; (vi) ) 0.5+X, 0.5-Y, 0.5+Z; (vii) -1+X, Y ,Z; (viii) X, 2.5-Y, 0.5+Z; (ix) X, 0.5-Y, 0.5+Z; (x) X, 0.5-Y, -0.5+Z; (xi) 2-X, 1-Y, 1-Z


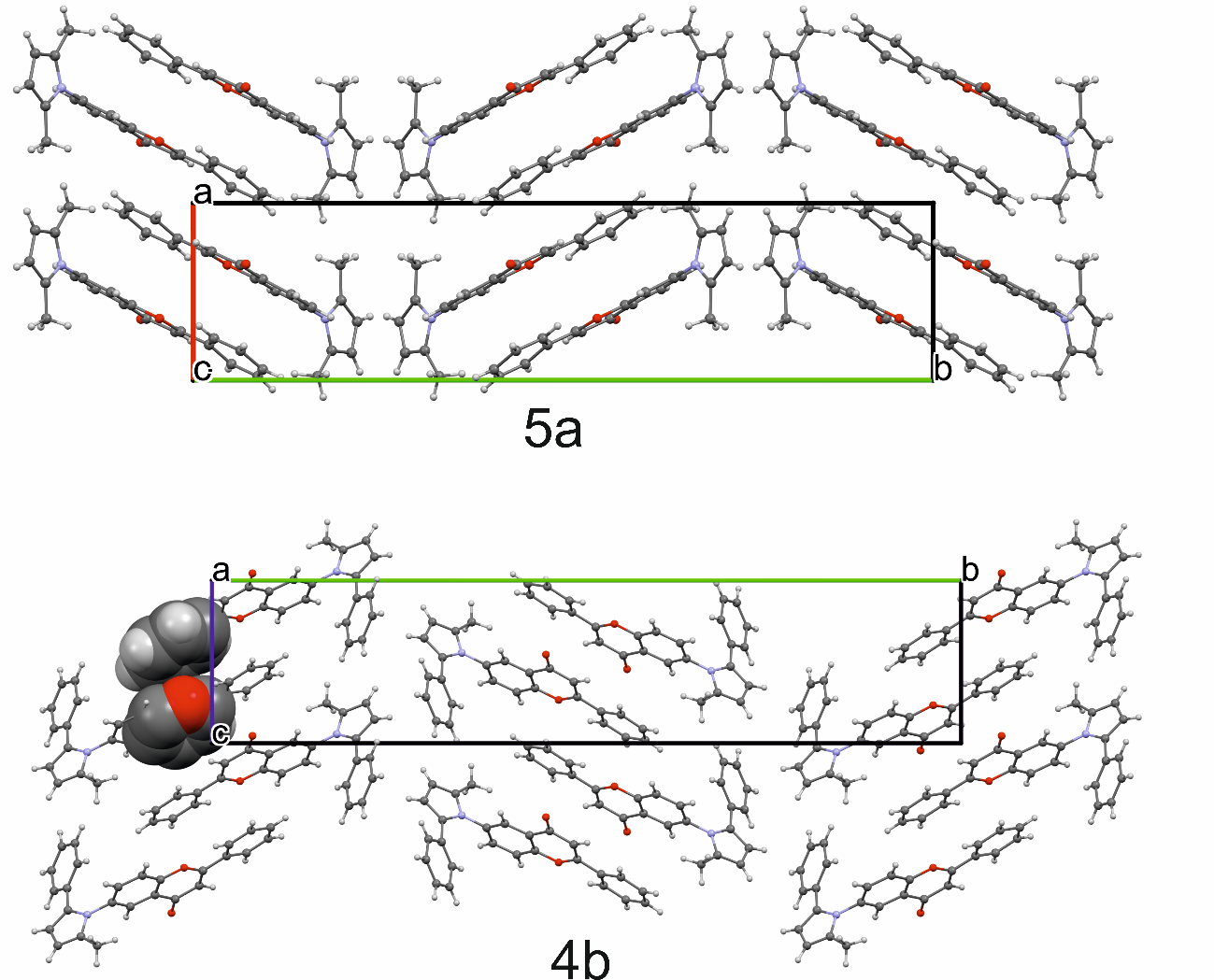


**Figure S4**. 3D structures in **5a** and **4b**. For the latter, one pair of overlay flavone phenyl and pyran-4-one rings are shown in a spacefill style.

**NMR data of 4a-4c and 5a-5c**

**6-(2,5-Dimethylpyrrol-1-yl)-2-phenyl-chromen-4-one (4a)**


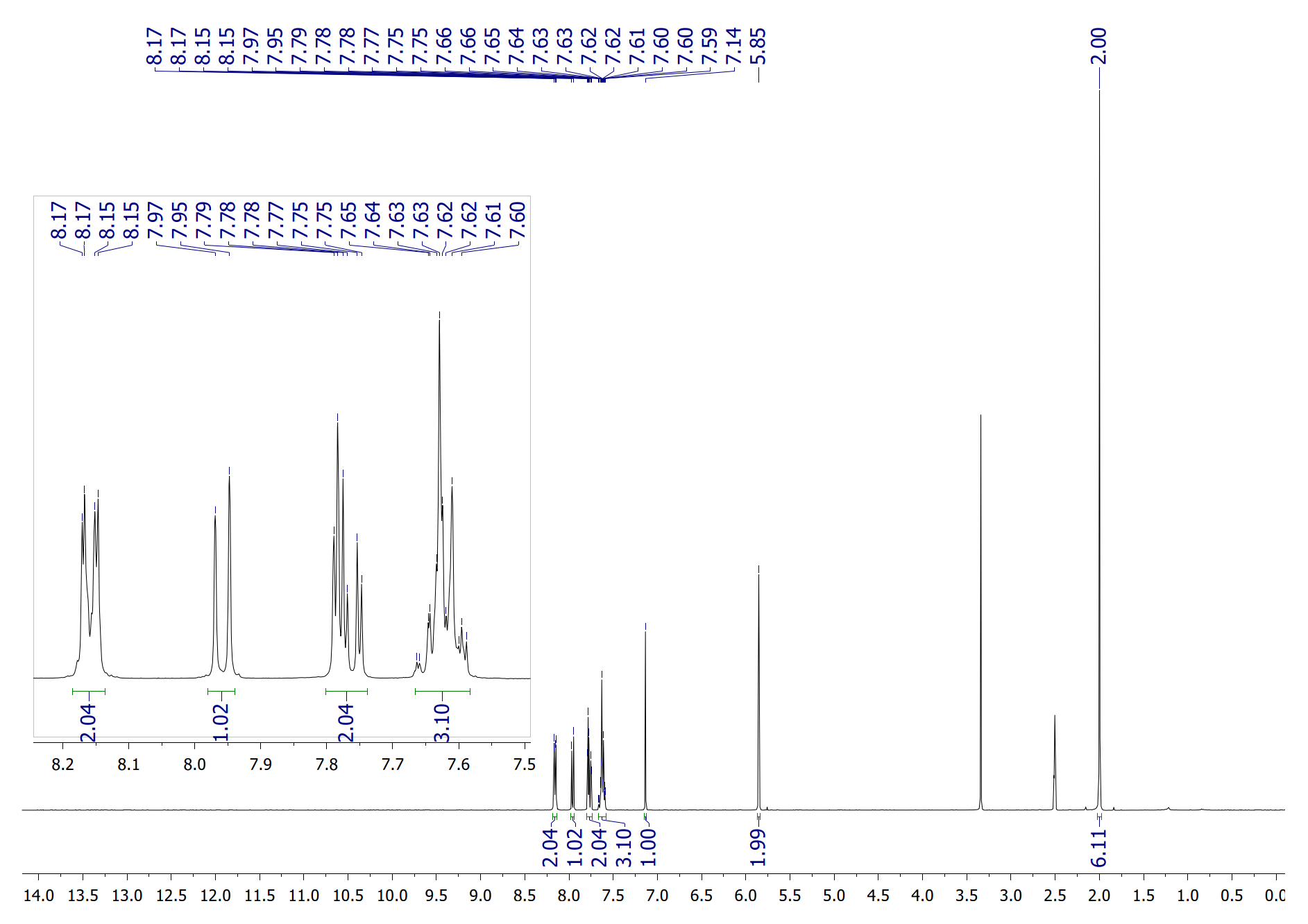

^1^H NMR spectrum of **4a** (DMSO-*d_6_*, 25°C).


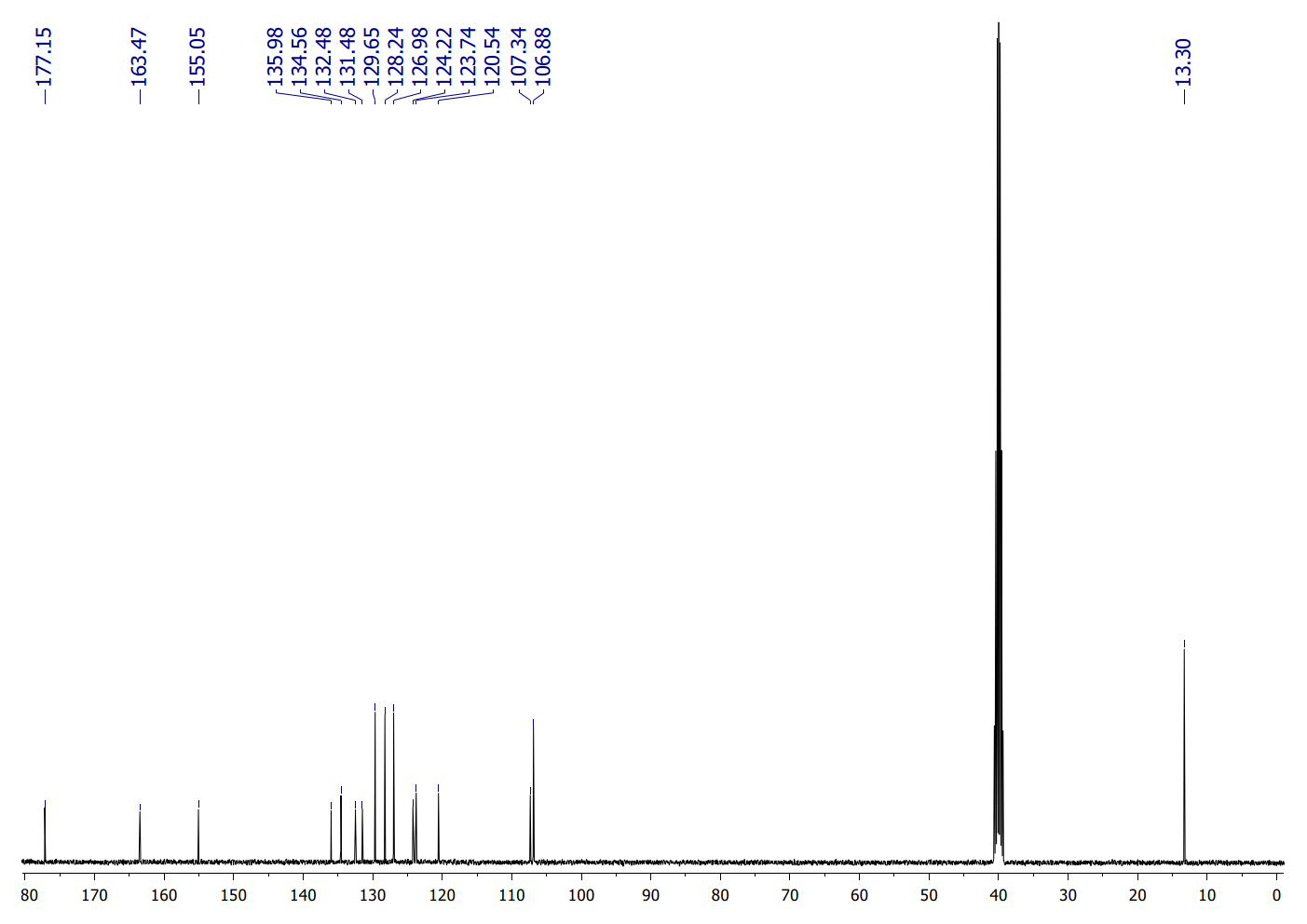

^13^C NMR spectrum of **4a** (DMSO-*d_6_*, 25°C).


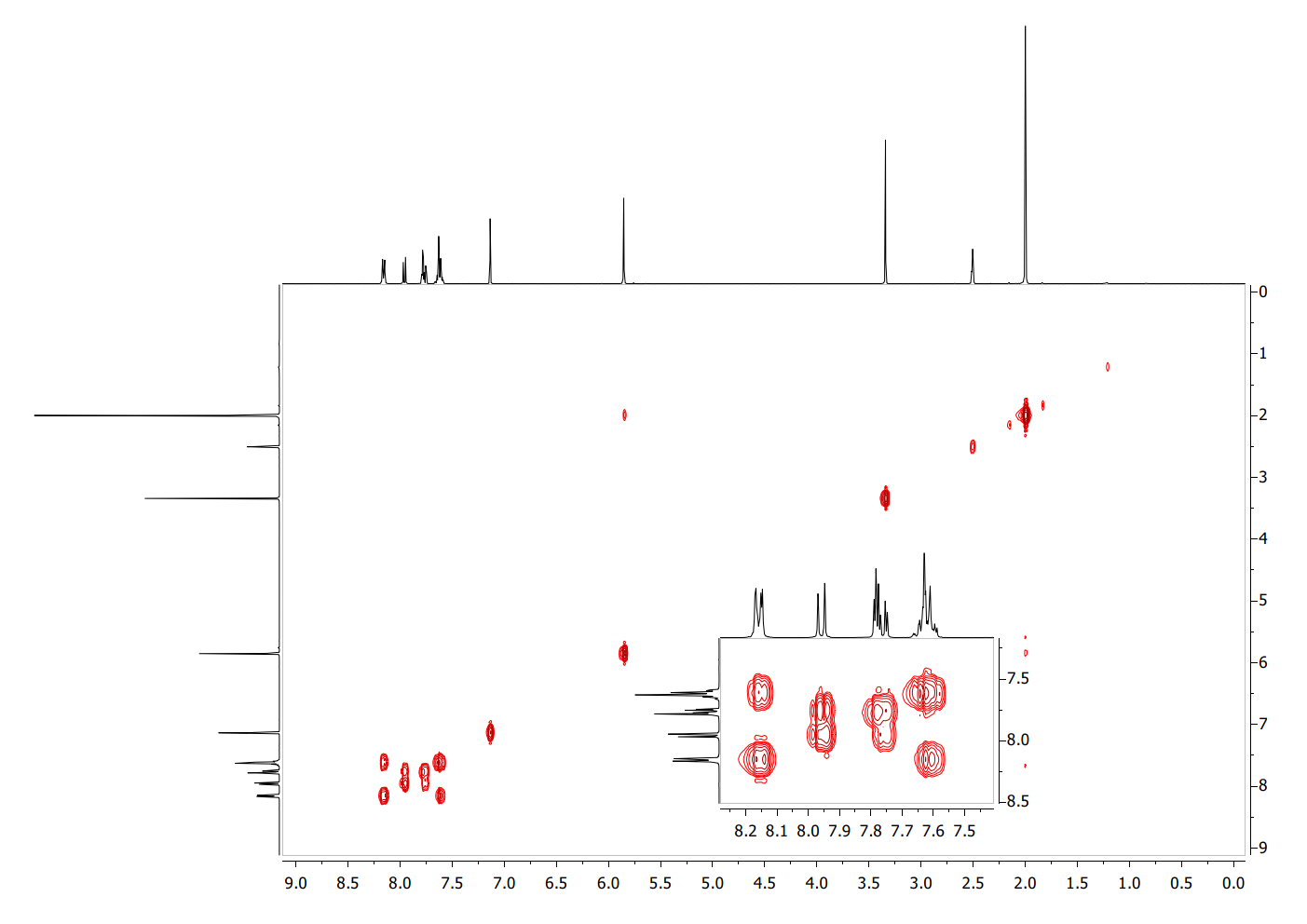

^1^H^1^H COSY NMR spectrum of **4a** (DMSO-*d_6_*, 25°C).


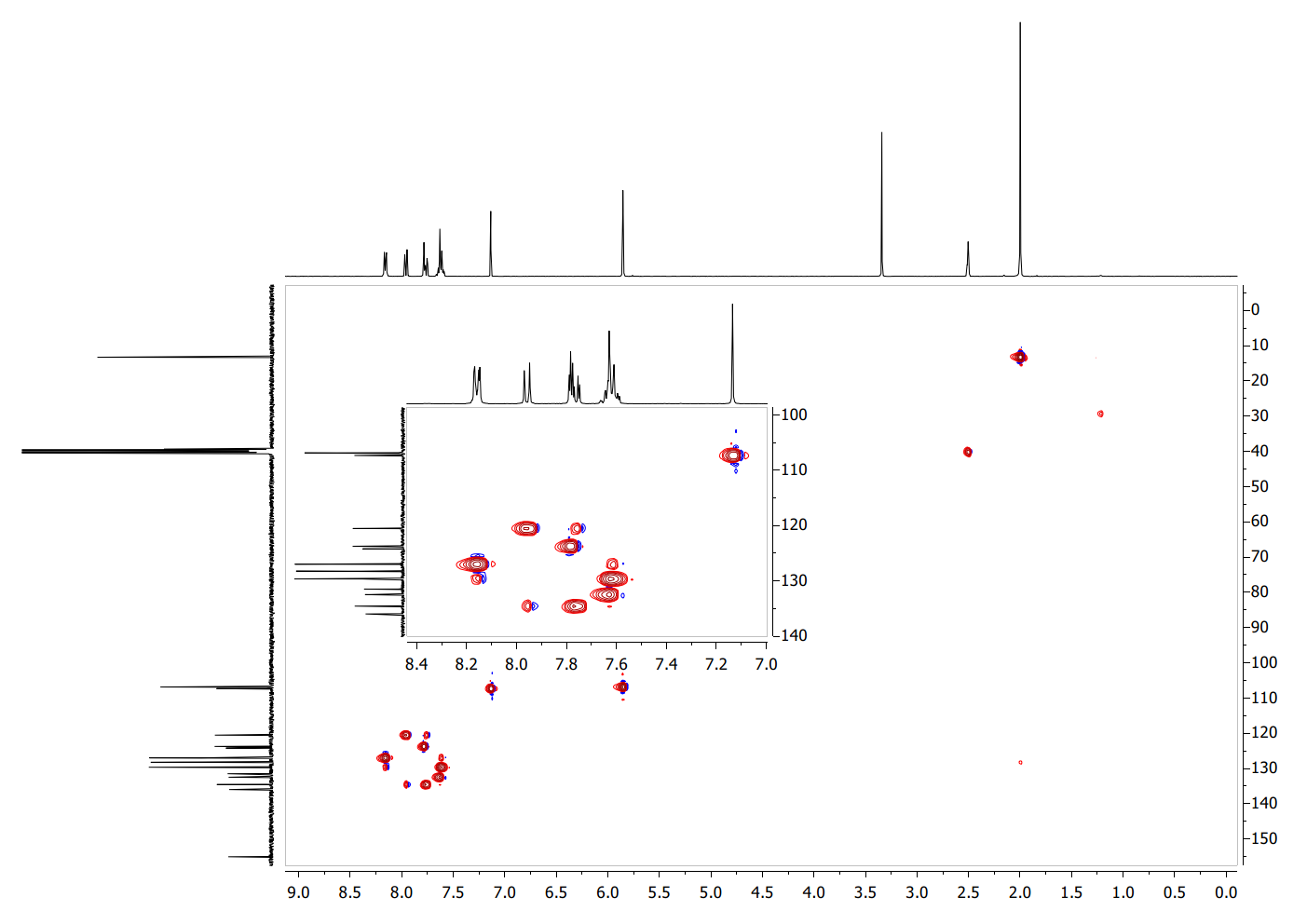

^1^H^13^C HSQC NMR spectrum of **4a** (DMSO-*d_6_*, 25°C).


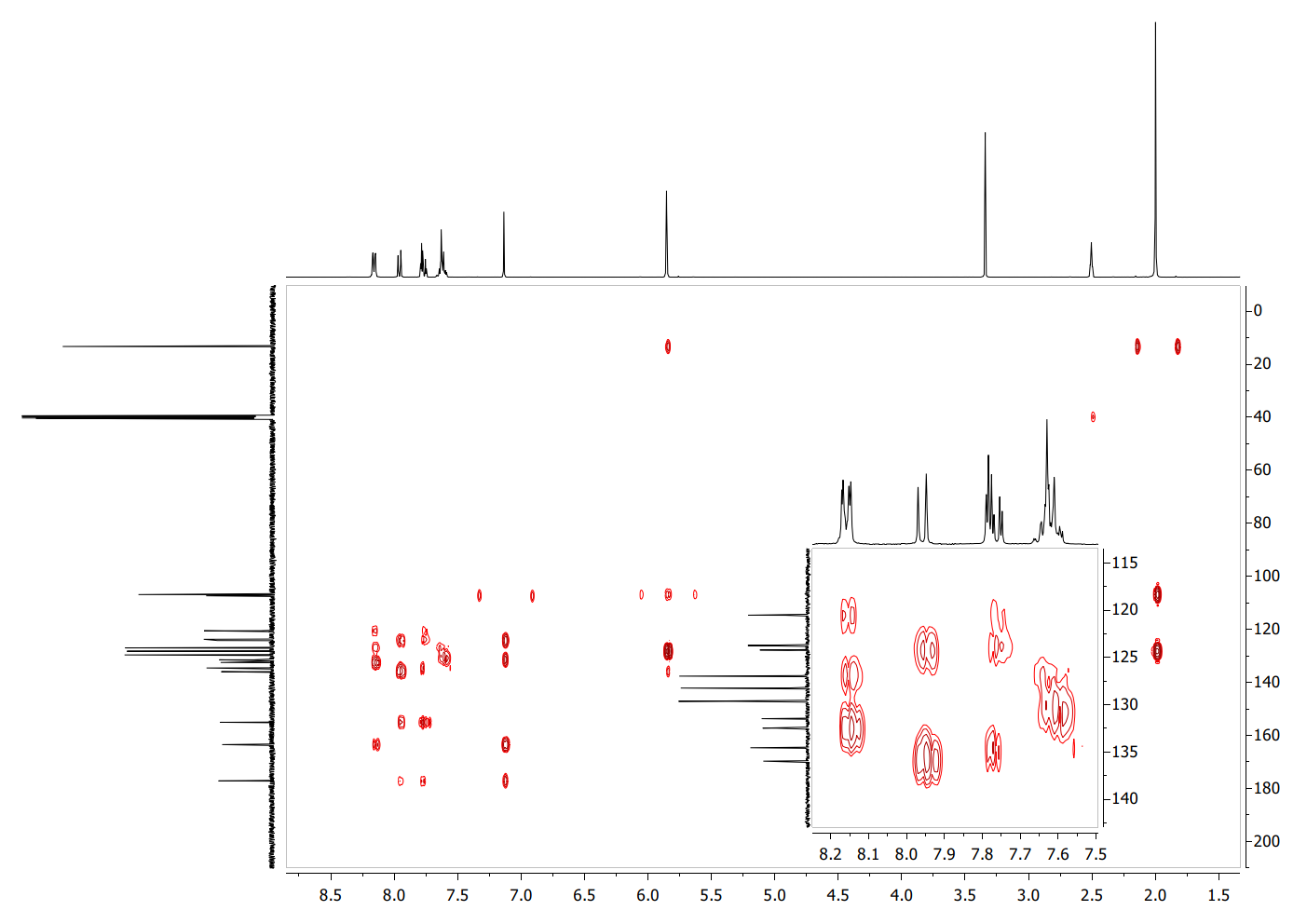

^1^H^13^C HMBC NMR spectrum of **4a** (DMSO-*d_6_*, 25°C).

**6-(2-Methyl-5-phenyl-pyrrol-1-yl)-2-phenyl-chromen-4-one (4b)**


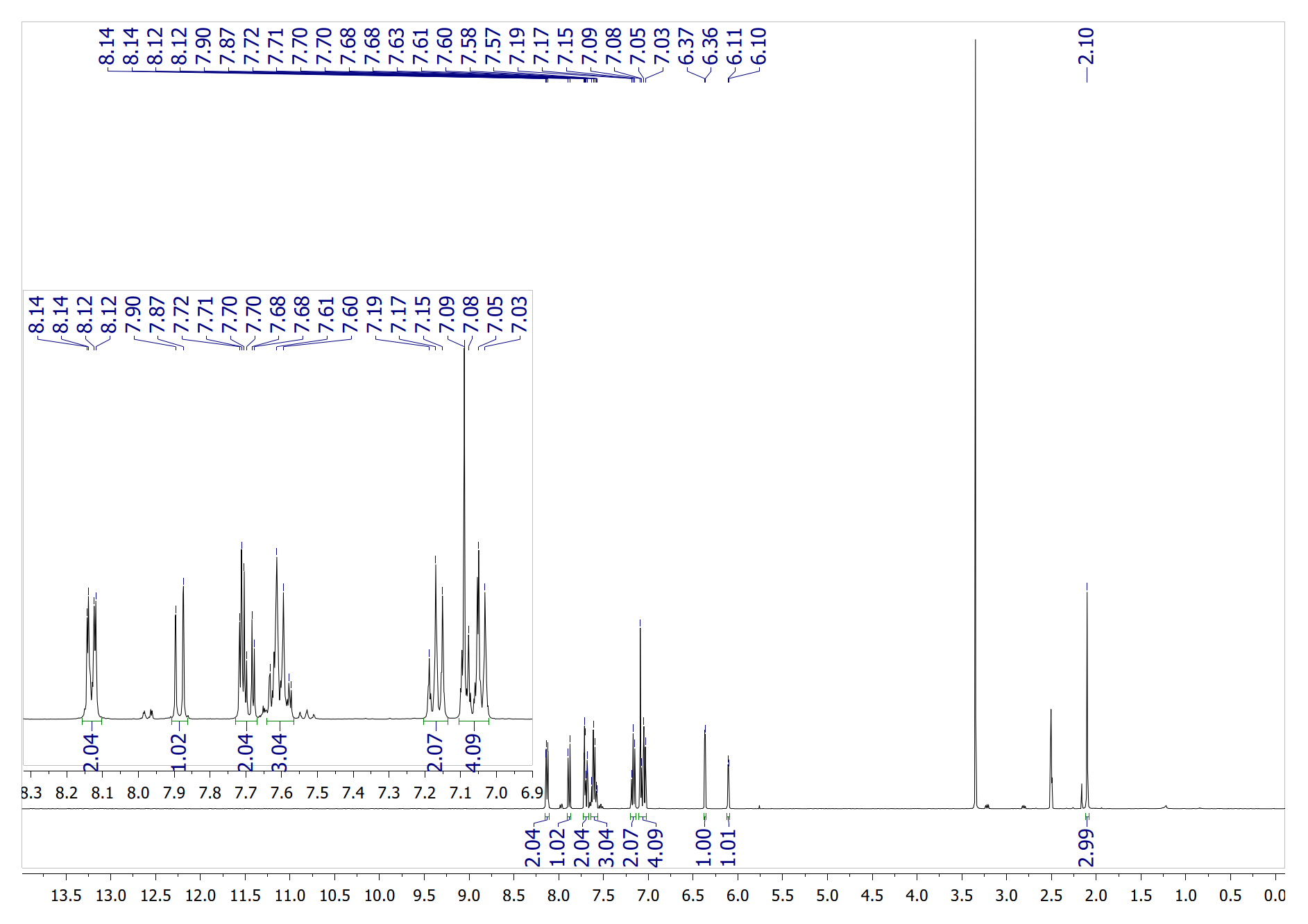

^1^H NMR spectrum of **4b** (DMSO-*d_6_*, 25°C).


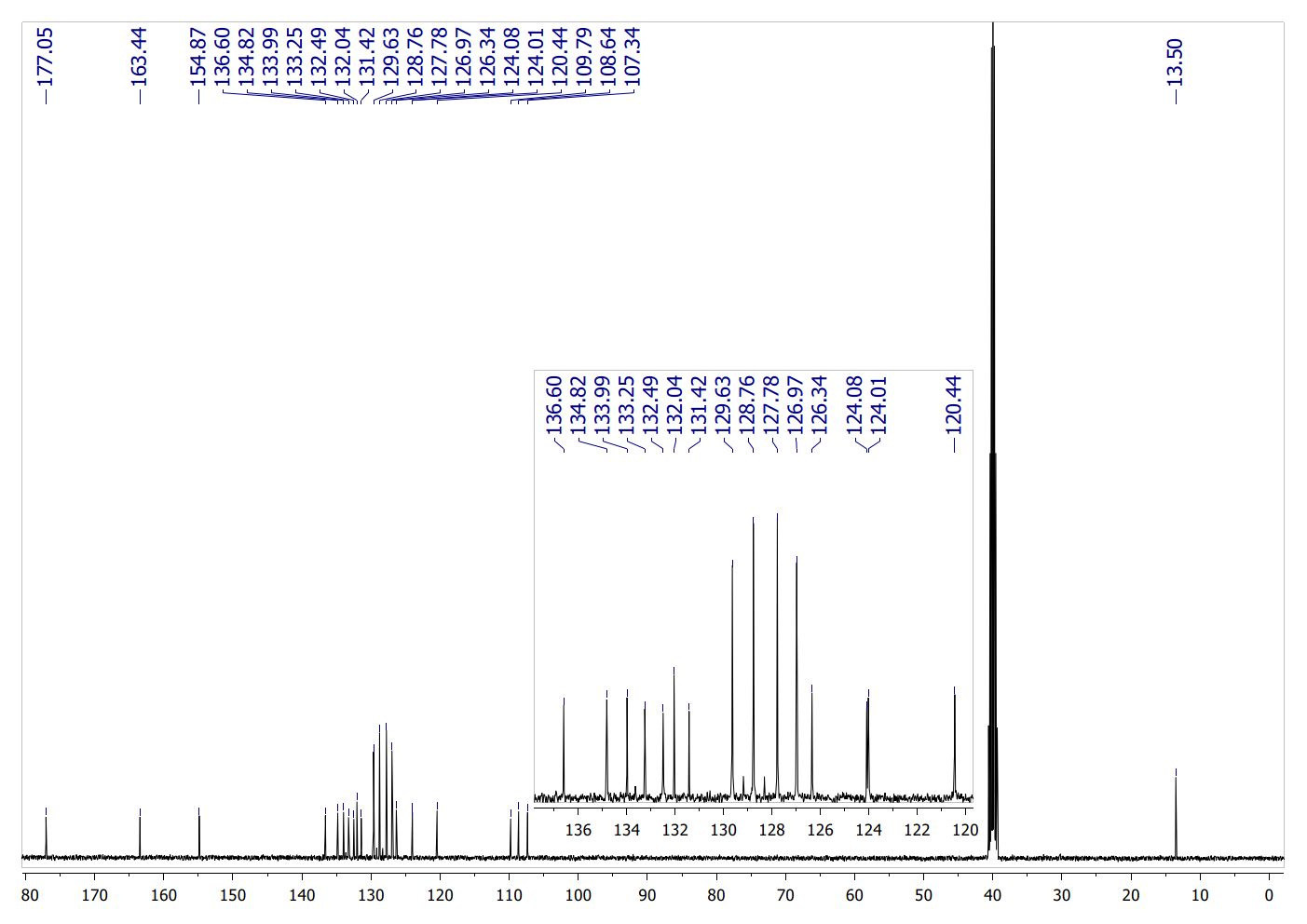

^13^C NMR spectrum of **4b** (DMSO-*d_6_*, 25°C).


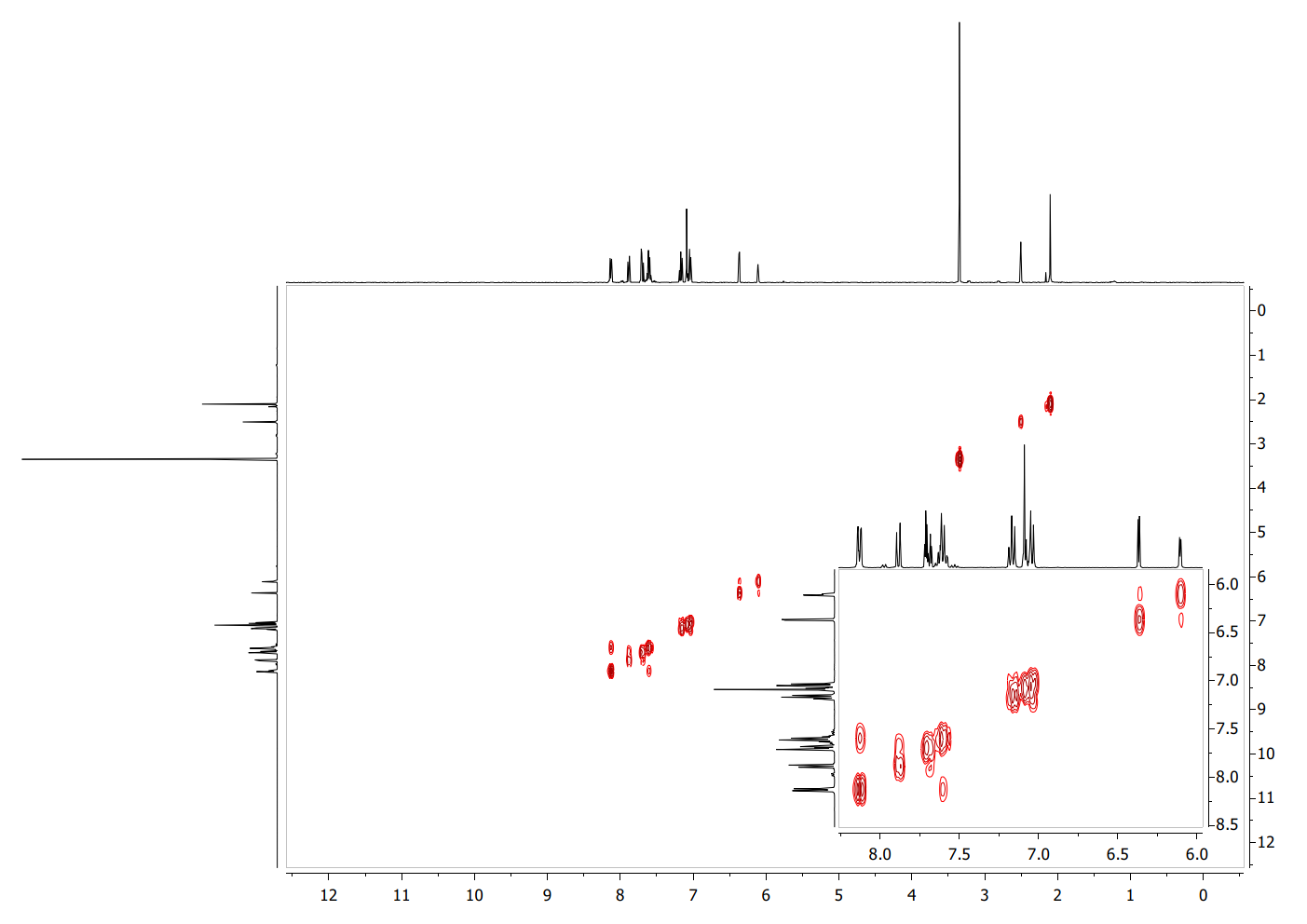

^1^H^1^H COSY NMR spectrum of **4b** (DMSO-*d_6_*, 25°C).


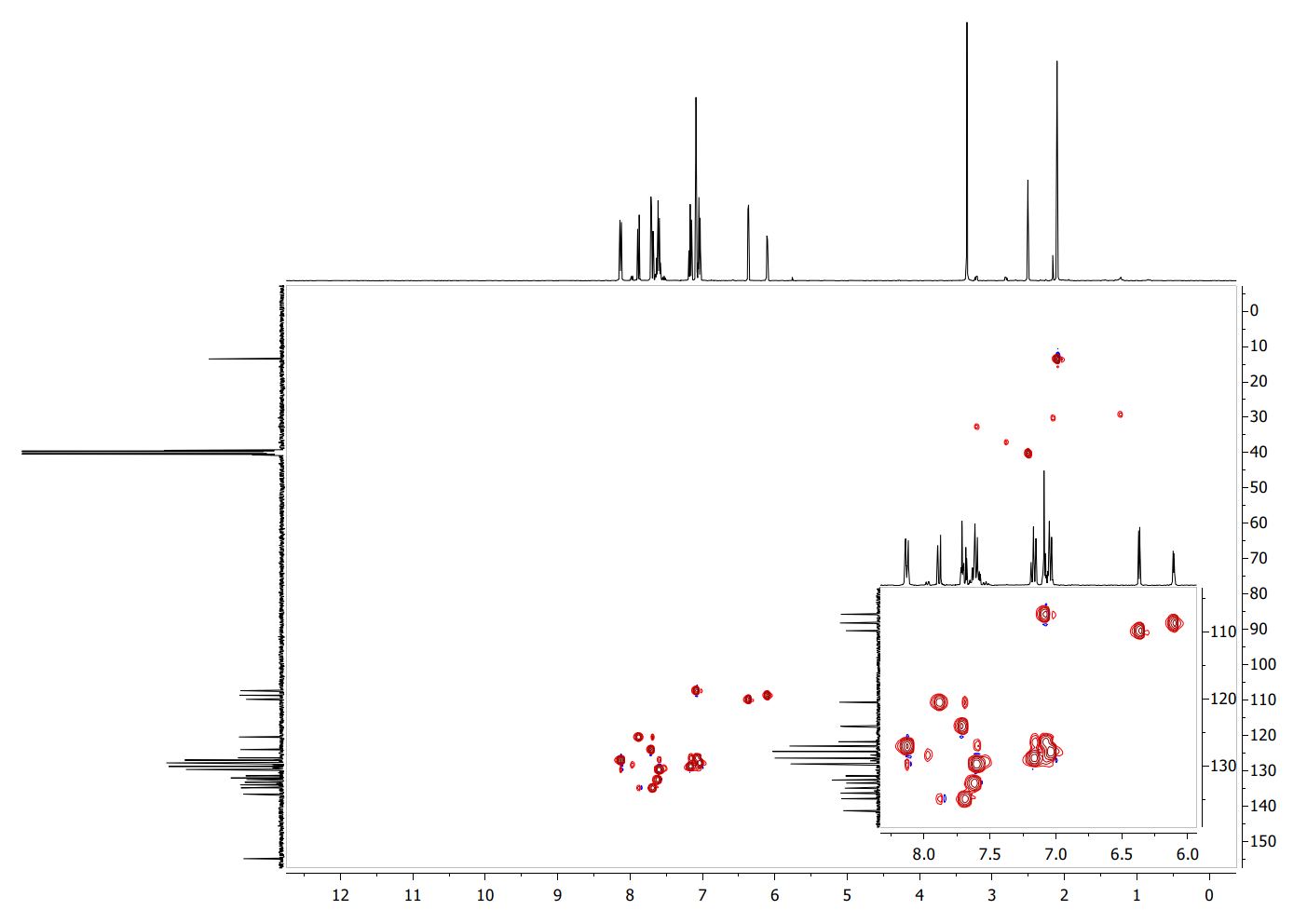

^1^H^13^C HSQC NMR spectrum of **4b** (DMSO-*d_6_*, 25°C).


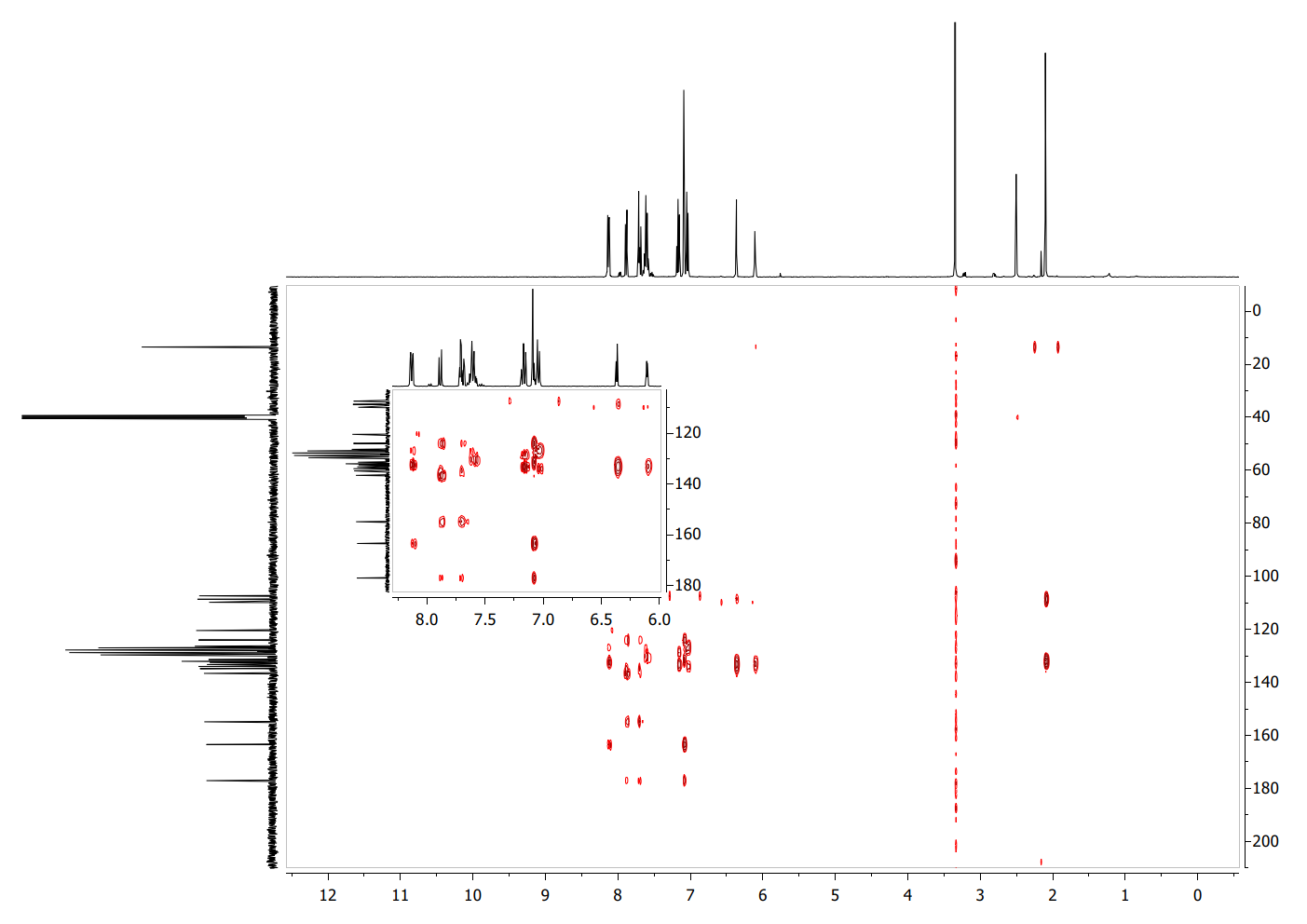

^1^H^13^C HMBC NMR spectrum of **4b** (DMSO-*d_6_*, 25°C).

**Diethyl 2,5-dimethyl-1-(4-oxo-2-phenyl-chromen-6-yl)pyrrole-3,4-dicarboxylate (4c)**


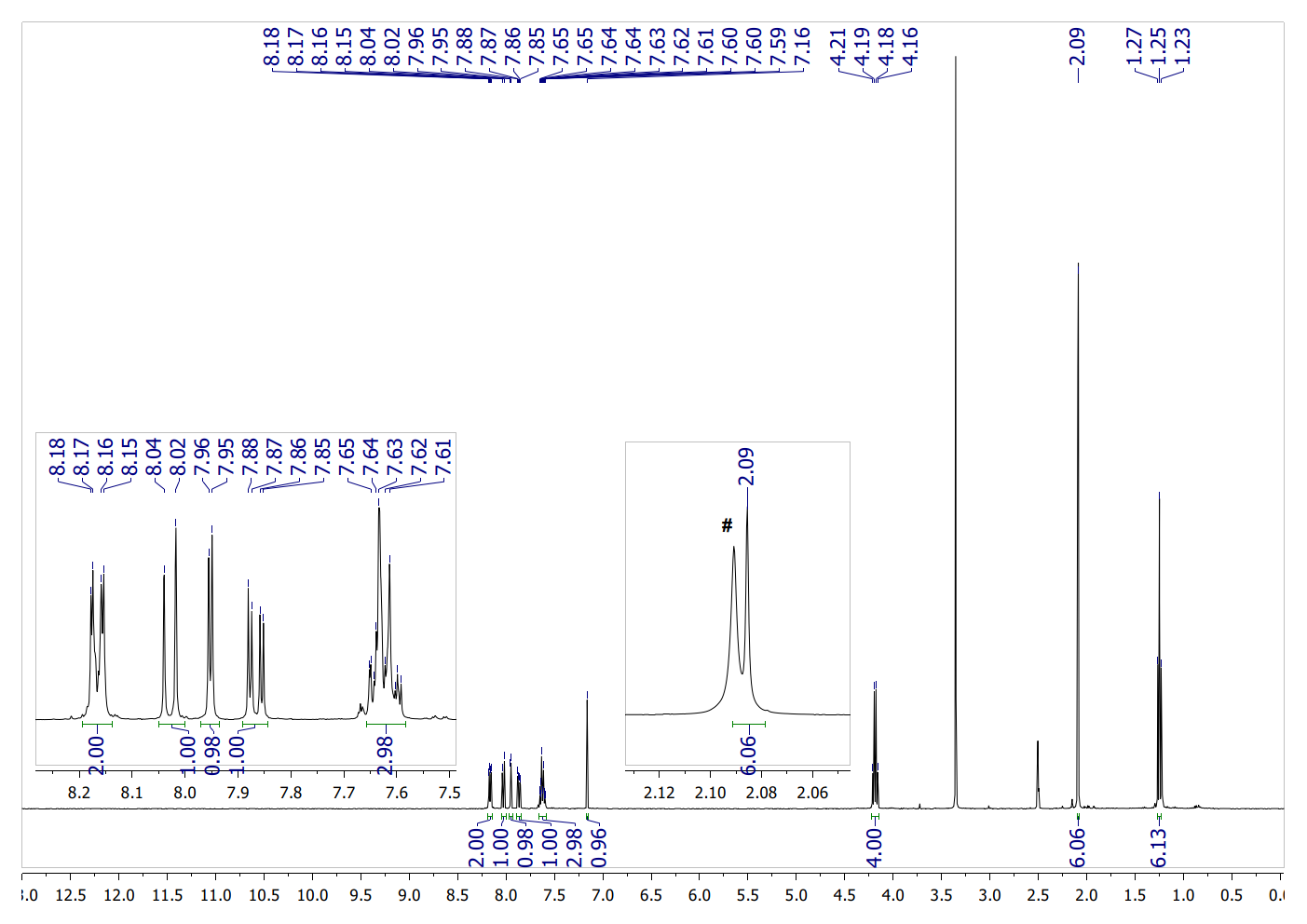

^1^H NMR spectrum of **4c** (DMSO-*d_6_*, 25°C) # - residual peak from acetone.


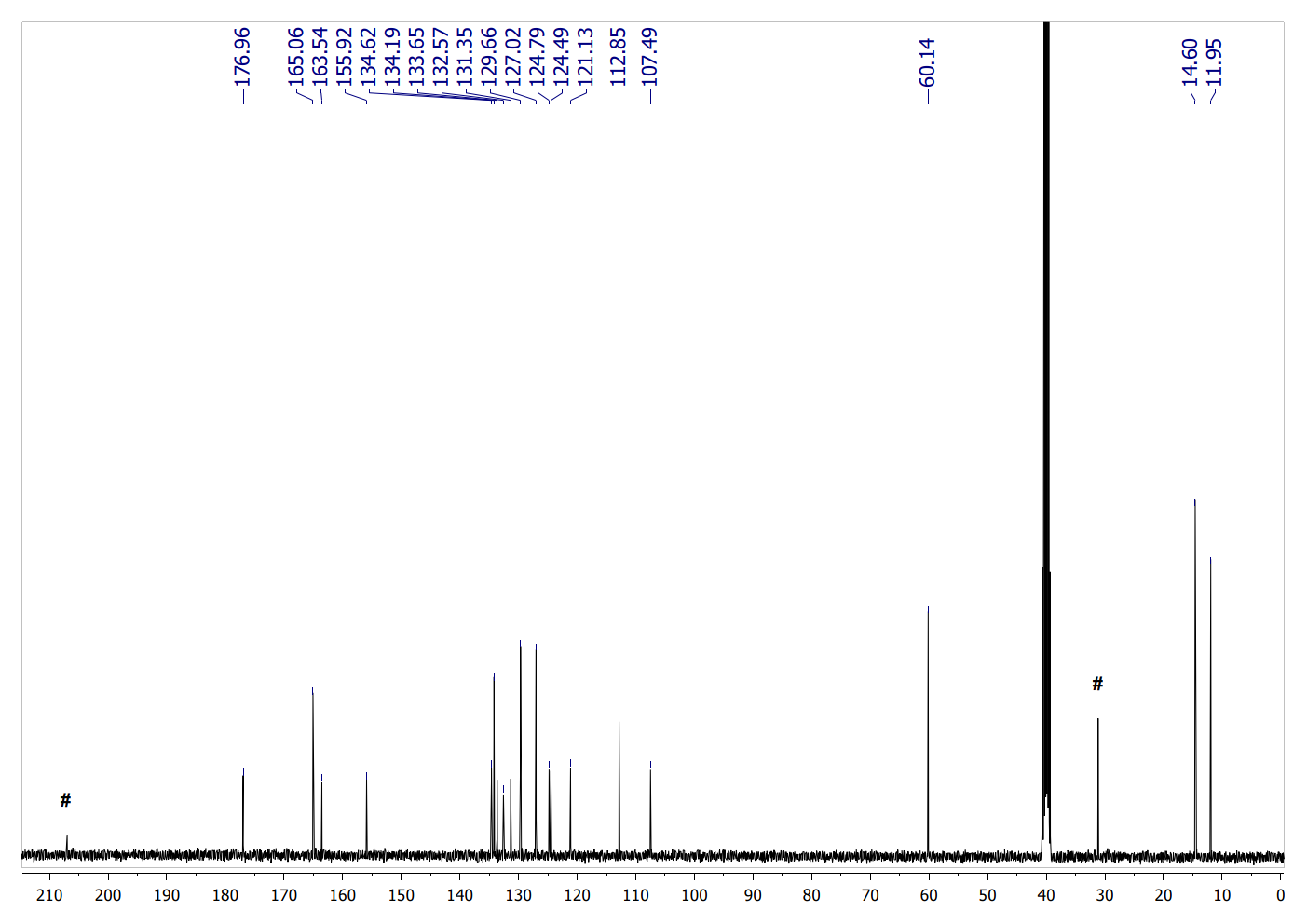

^13^C NMR spectrum of **4c** (DMSO-*d_6_*, 25°C) # - residual peaks from acetone.


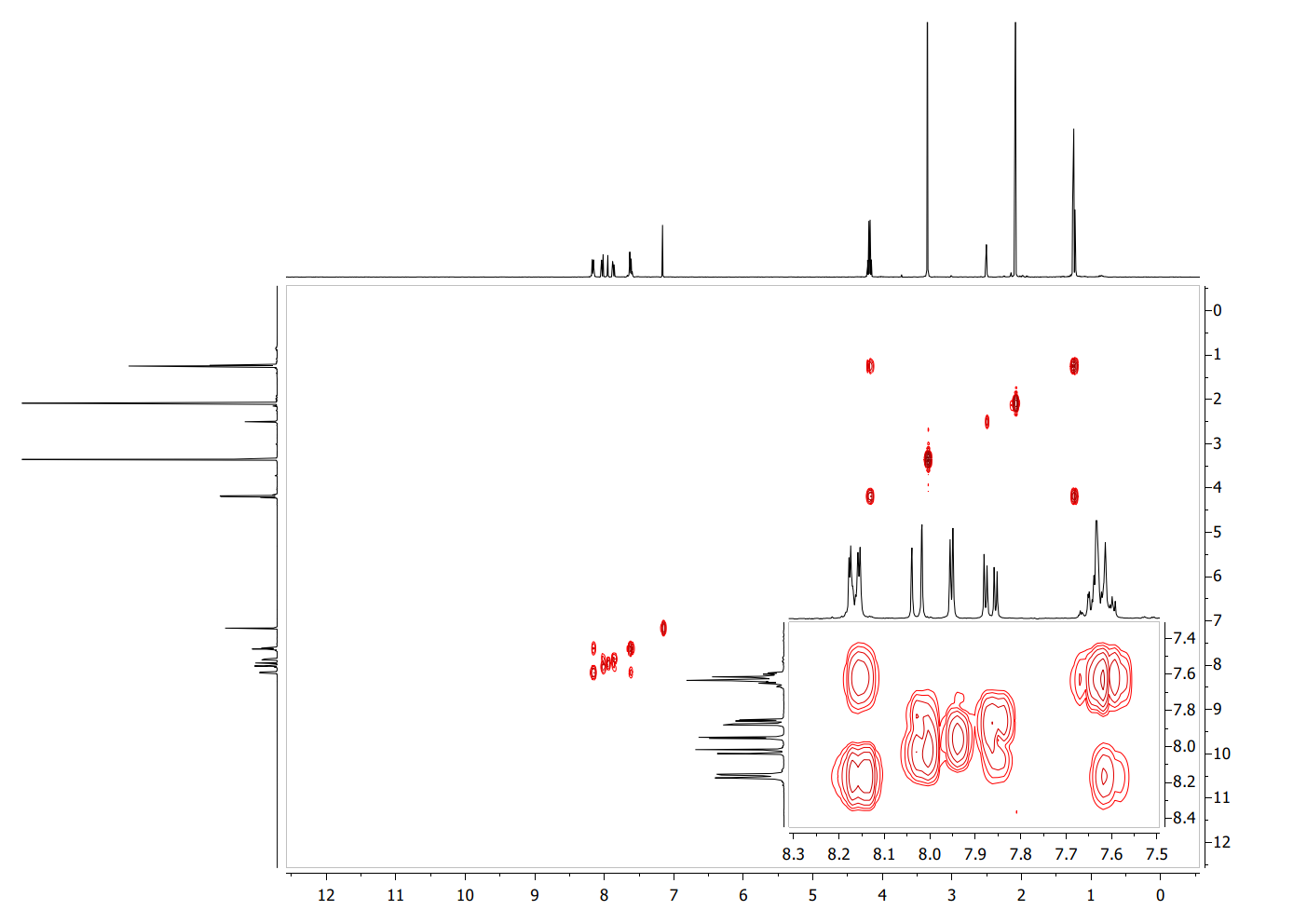

^1^H^1^H COSY NMR spectrum of **4c** (DMSO-*d_6_*, 25°C).


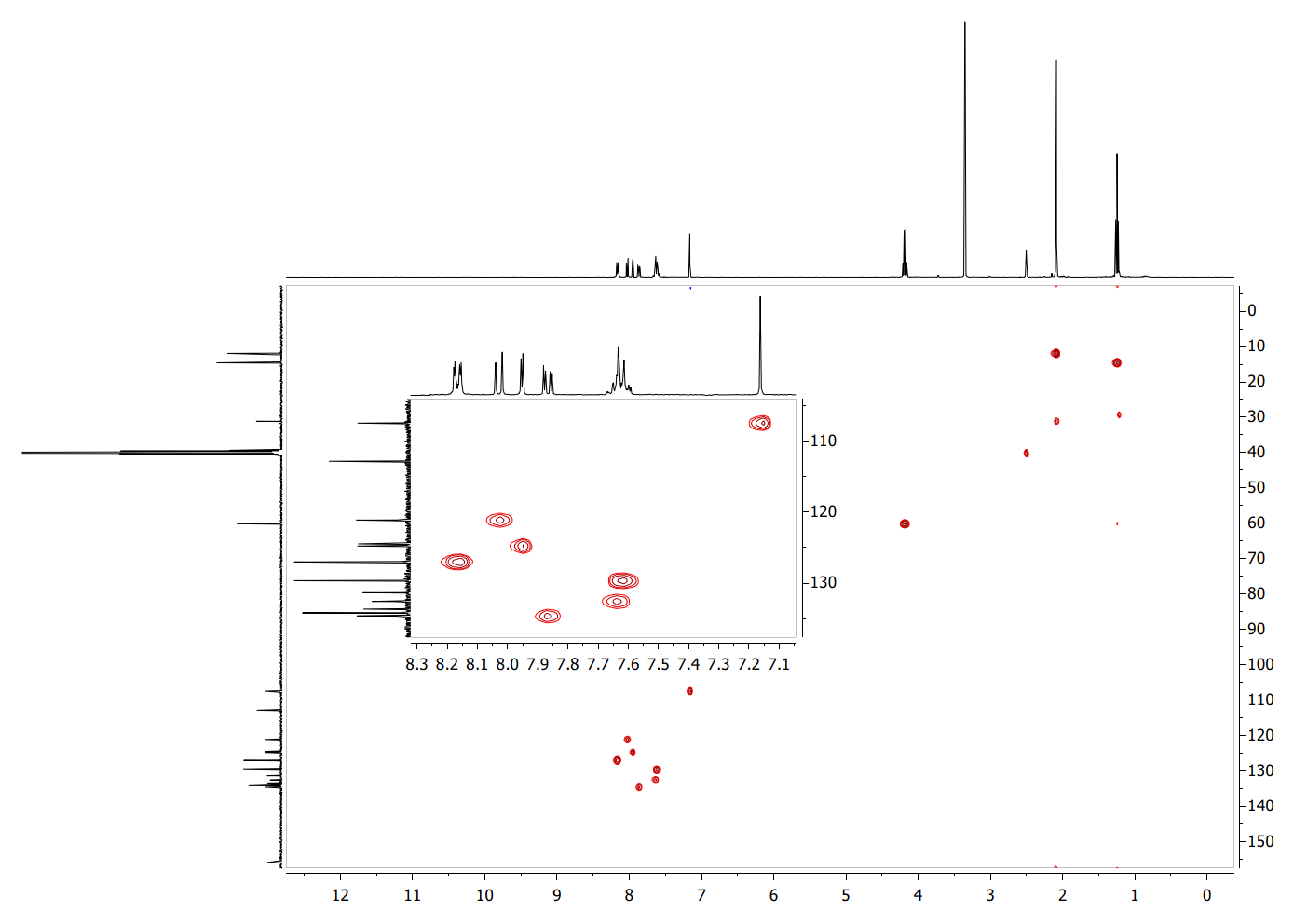

^1^H^13^C HSQC NMR spectrum of **4c** (DMSO-*d_6_*, 25°C).


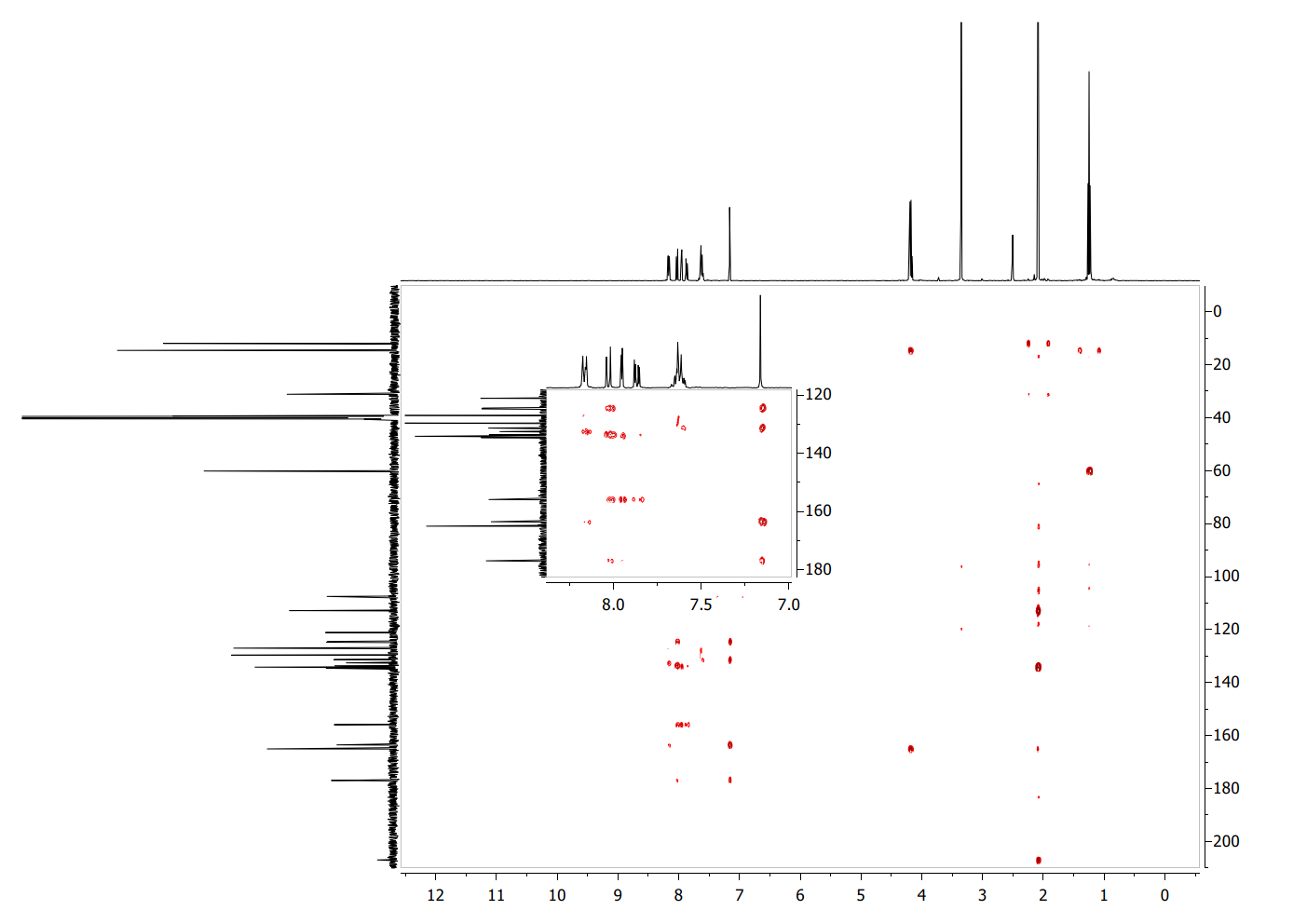

^1^H^13^C HMBC NMR spectrum of **4c** (DMSO-*d_6_*, 25°C).

**7-(2,5-Dimethylpyrrol-1-yl)-2-phenyl-chromen-4-one (5a)**


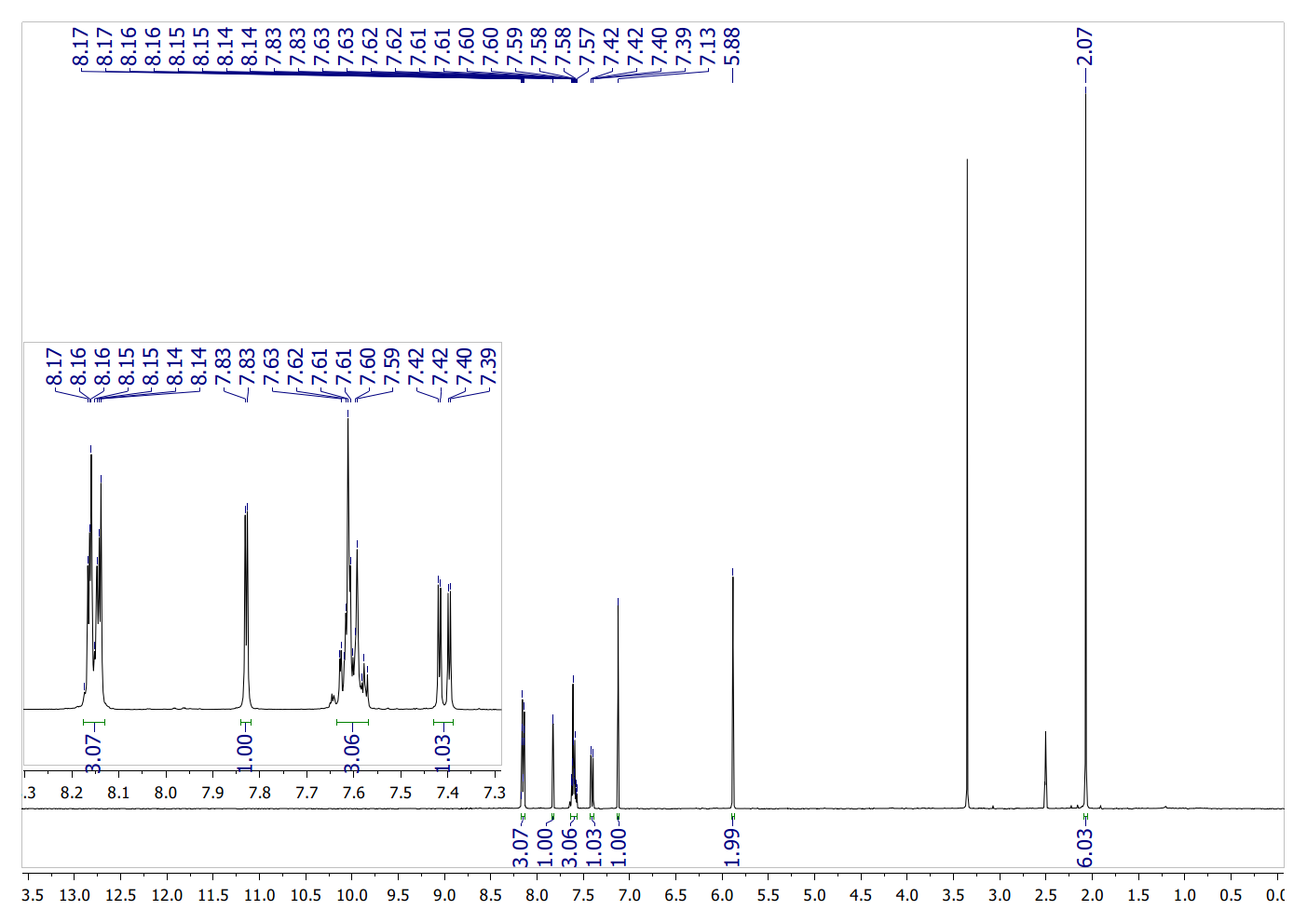

^1^H NMR spectrum of **5a** (DMSO-*d_6_*, 25°C).


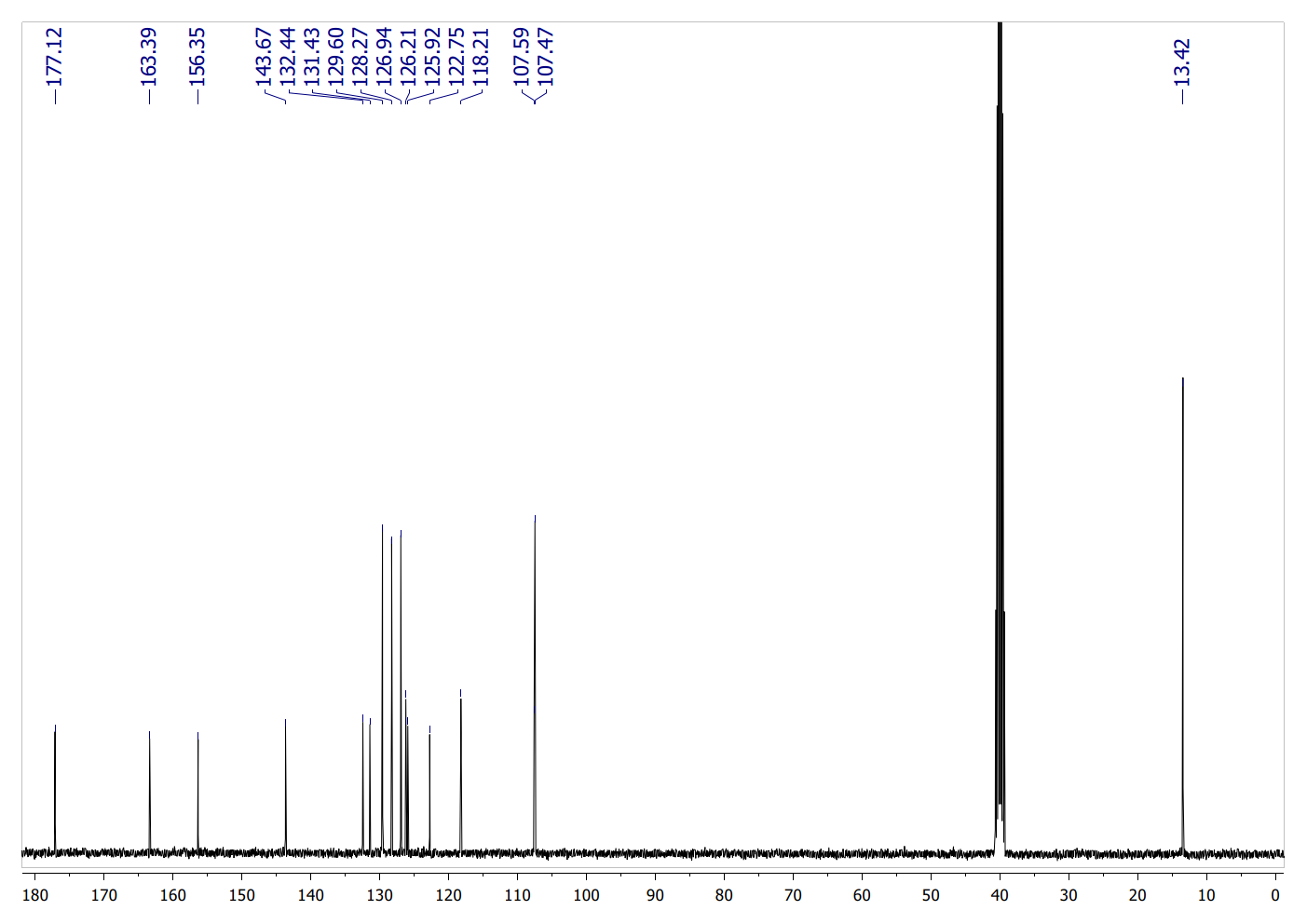

^13^C NMR spectrum of **5a** (DMSO-*d_6_*, 25°C).


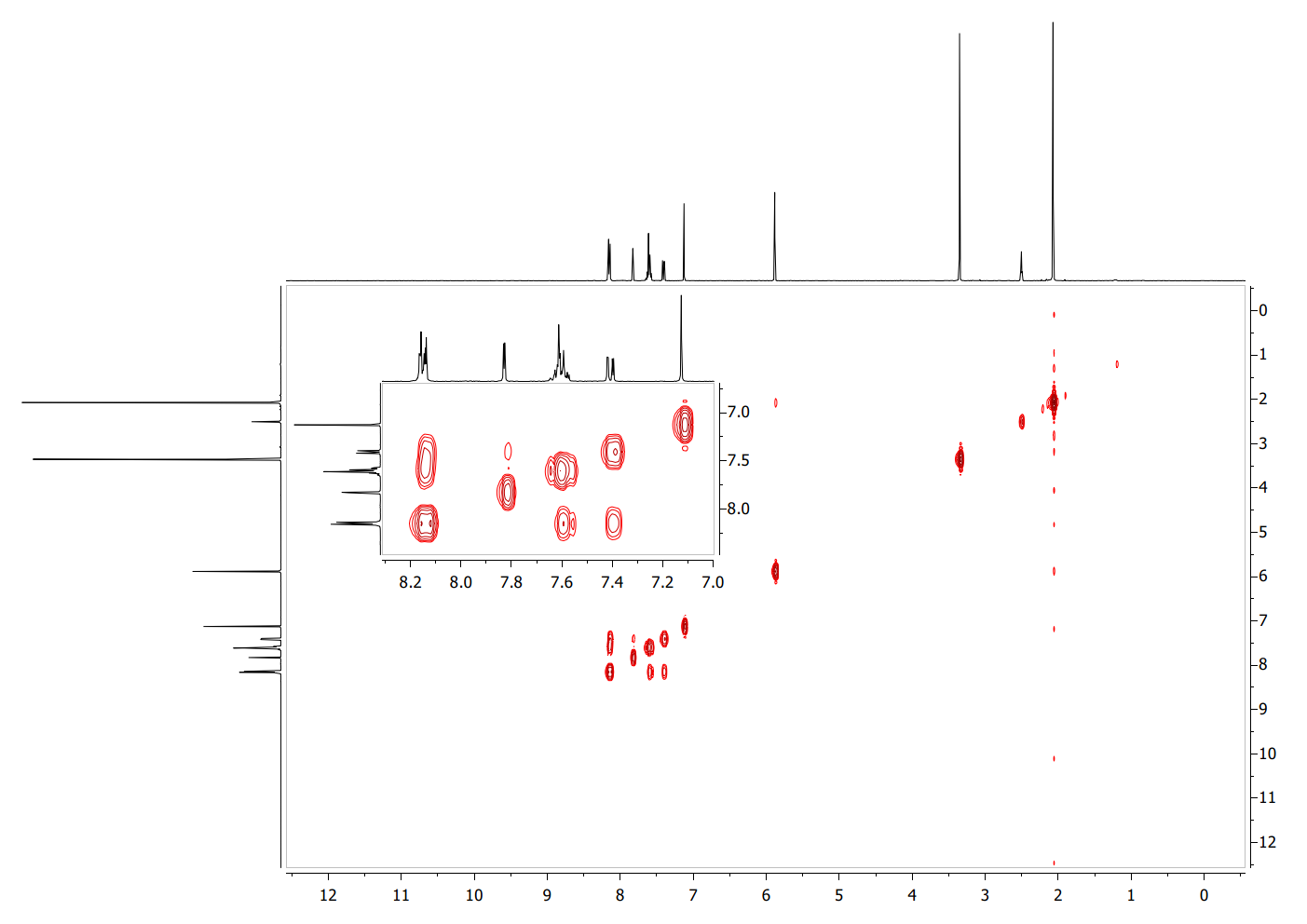
 ^1^H^1^H COSY NMR spectrum of **5a** (DMSO-*d_6_*, 25°C).


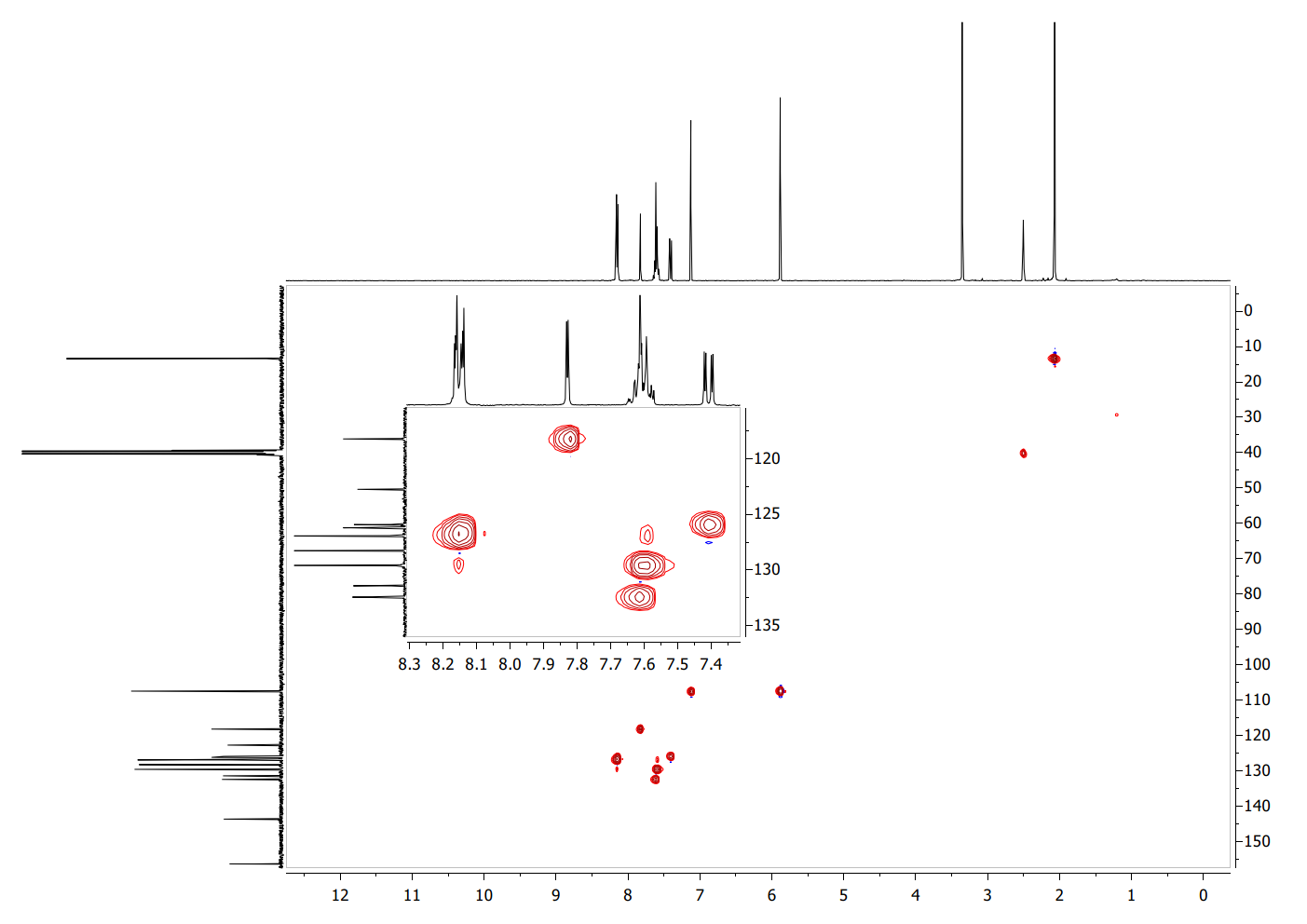

^1^H^13^C HSQC NMR spectrum of **5a** (DMSO-*d_6_*, 25°C).


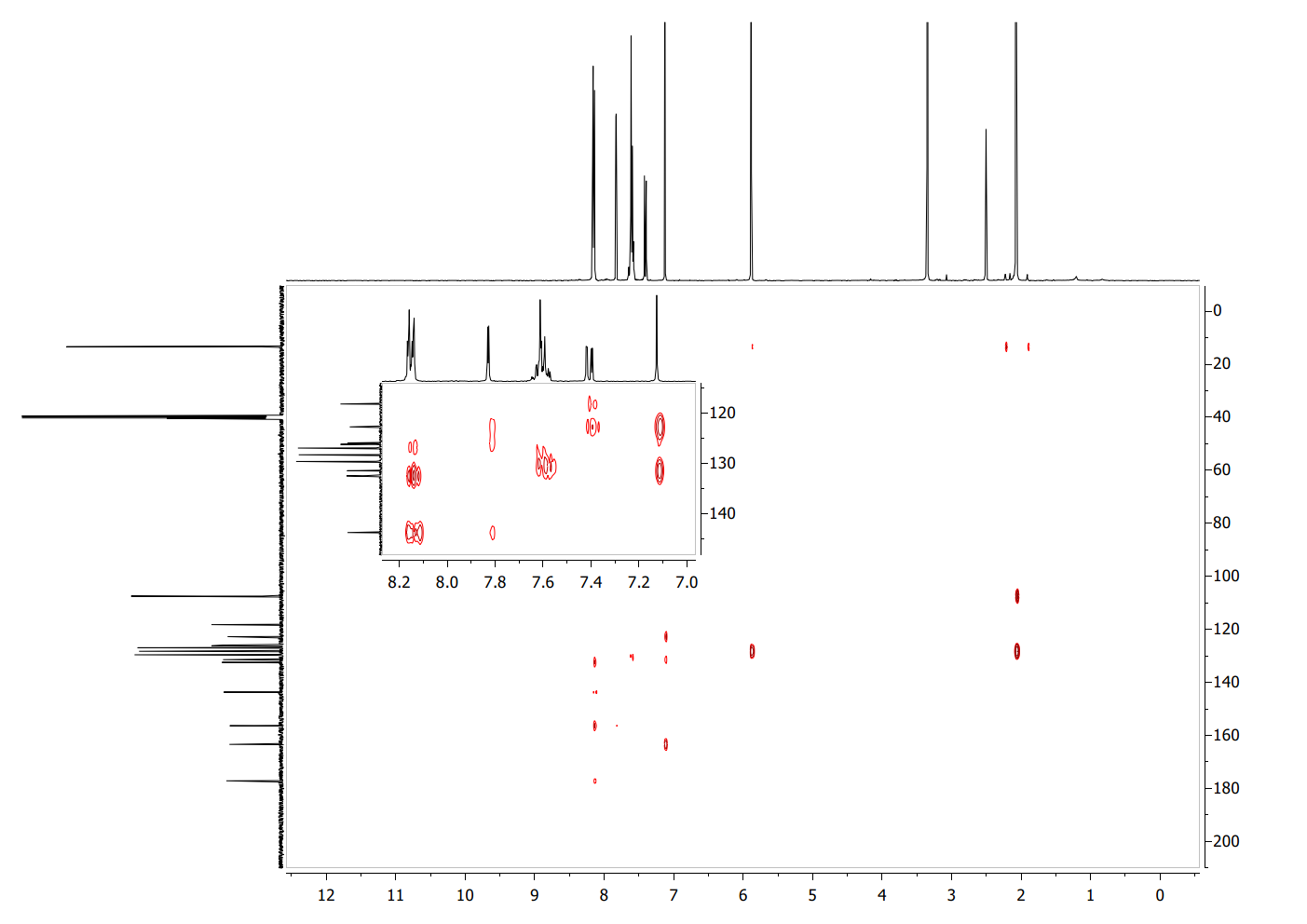

^1^H^13^C HMBC NMR spectrum of **5a** (DMSO-*d_6_*, 25°C).

**7-(2-Methyl-5-phenyl-pyrrol-1-yl)-2-phenyl-chromen-4-one (5b)**


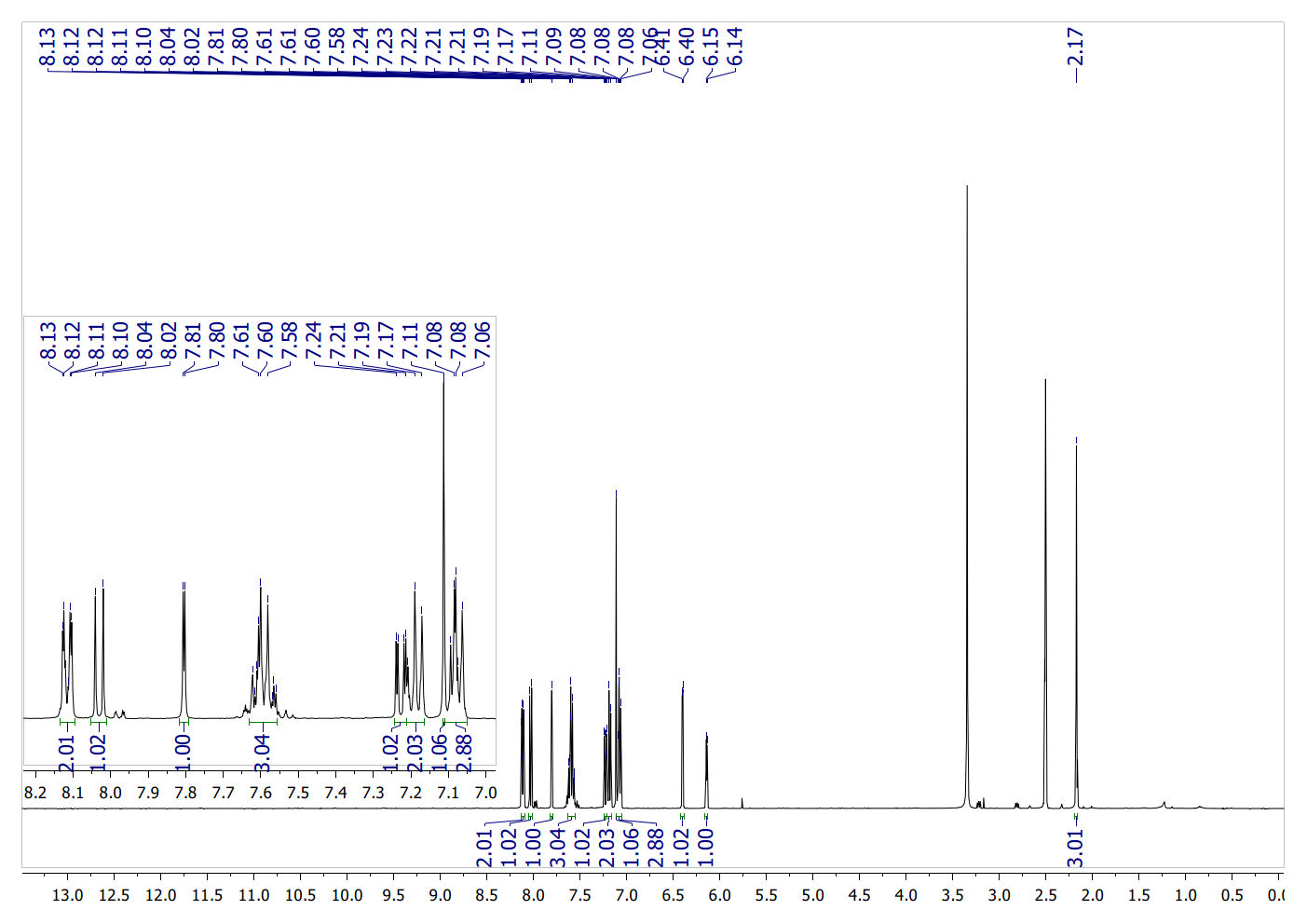

^1^H NMR spectrum of **5b** (DMSO-*d_6_*, 25°C).


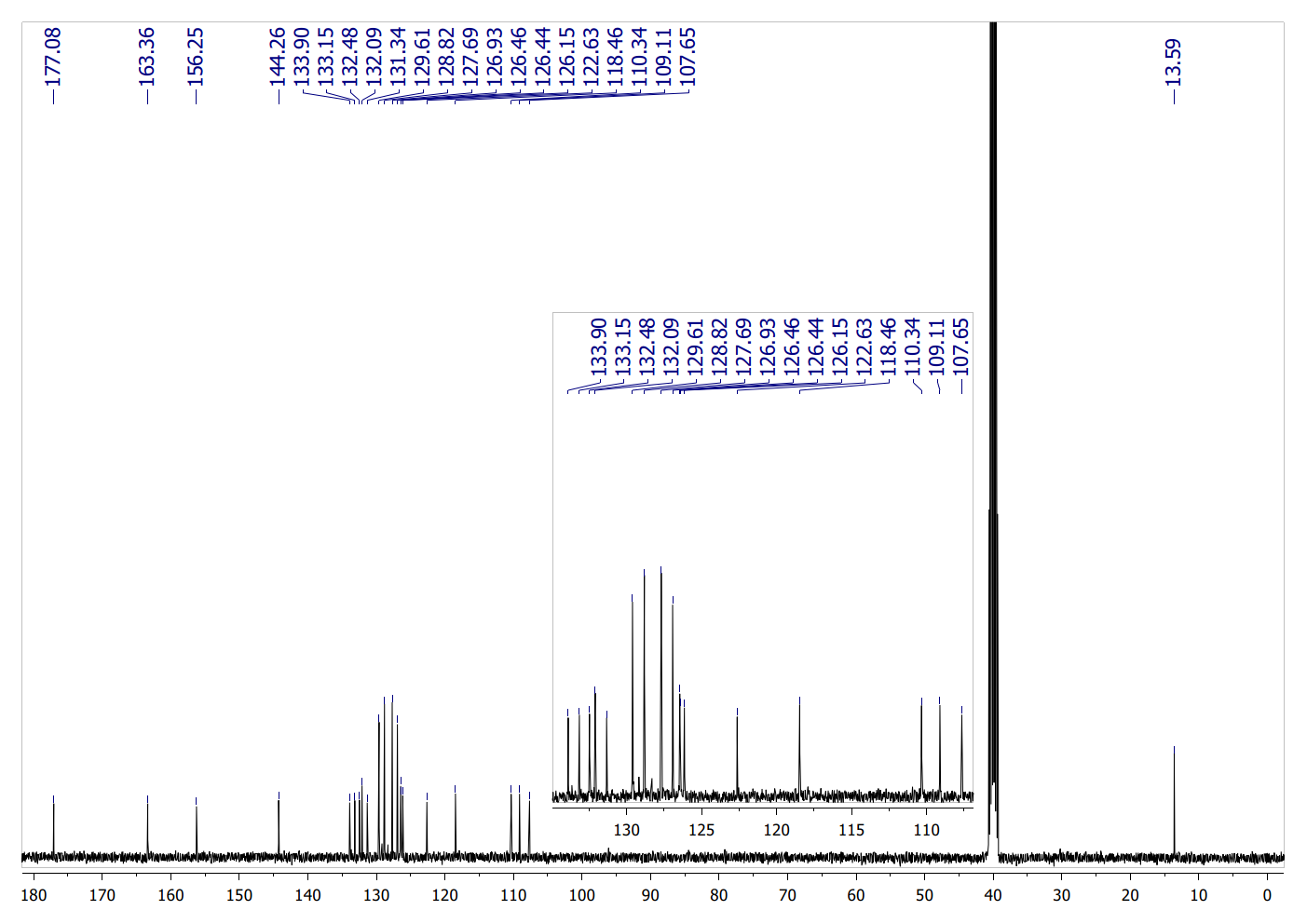

^13^C NMR spectrum of **5b** (DMSO-*d_6_*, 25°C).


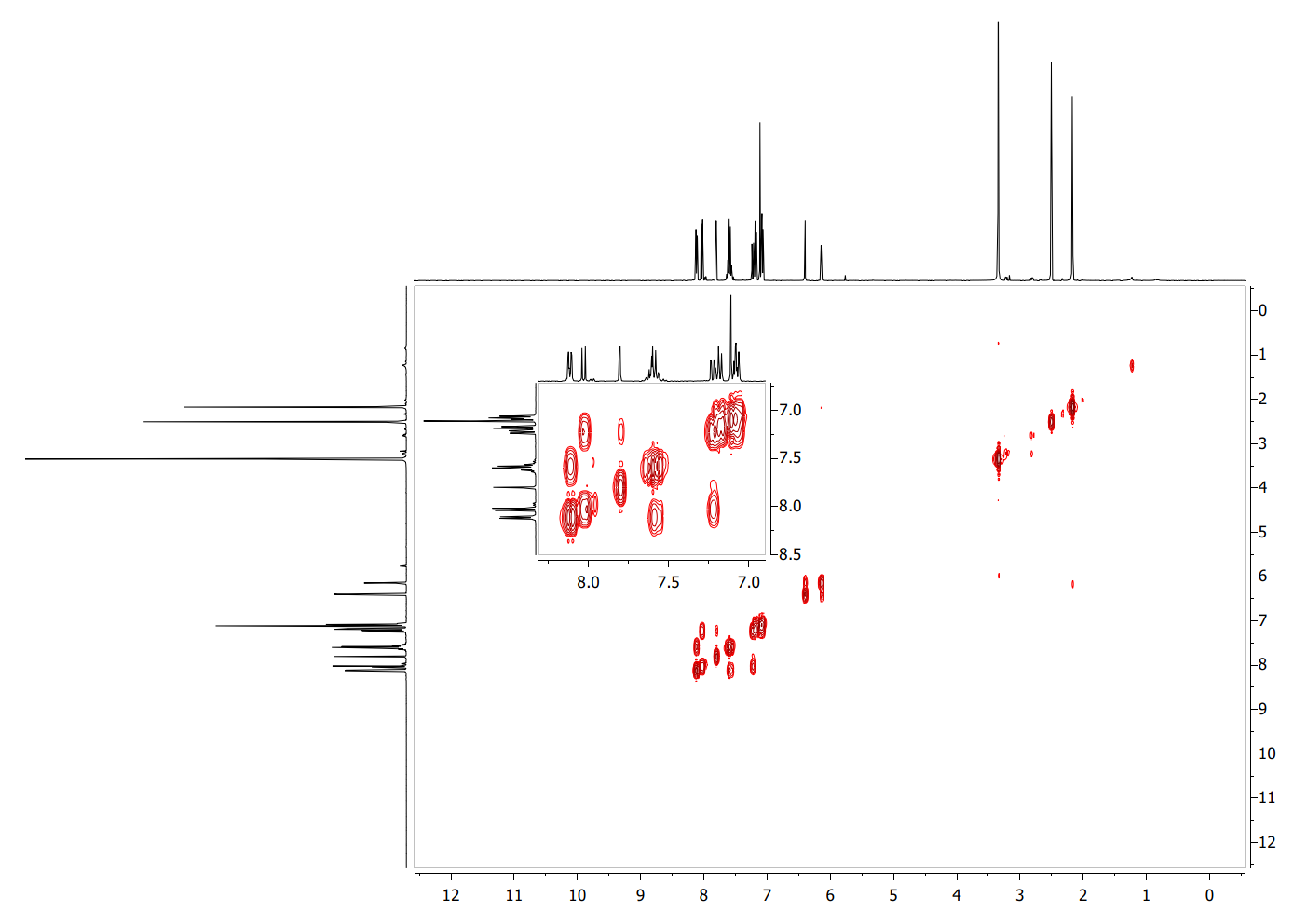

^1^H^1^H COSY NMR spectrum of **5b** (DMSO-*d_6_*, 25°C).


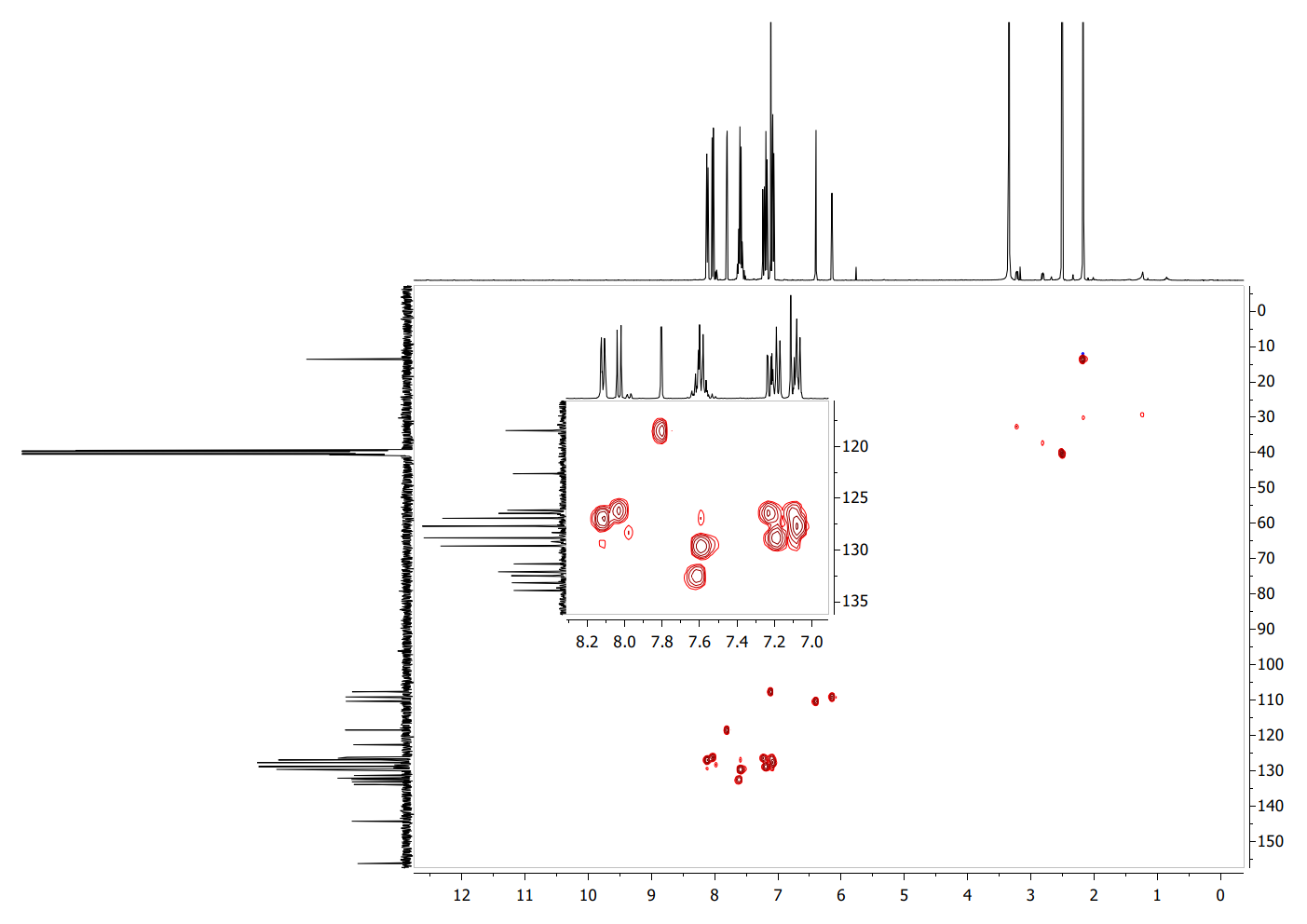

^1^H^13^C HSQC NMR spectrum of **5b** (DMSO-*d_6_*, 25°C).


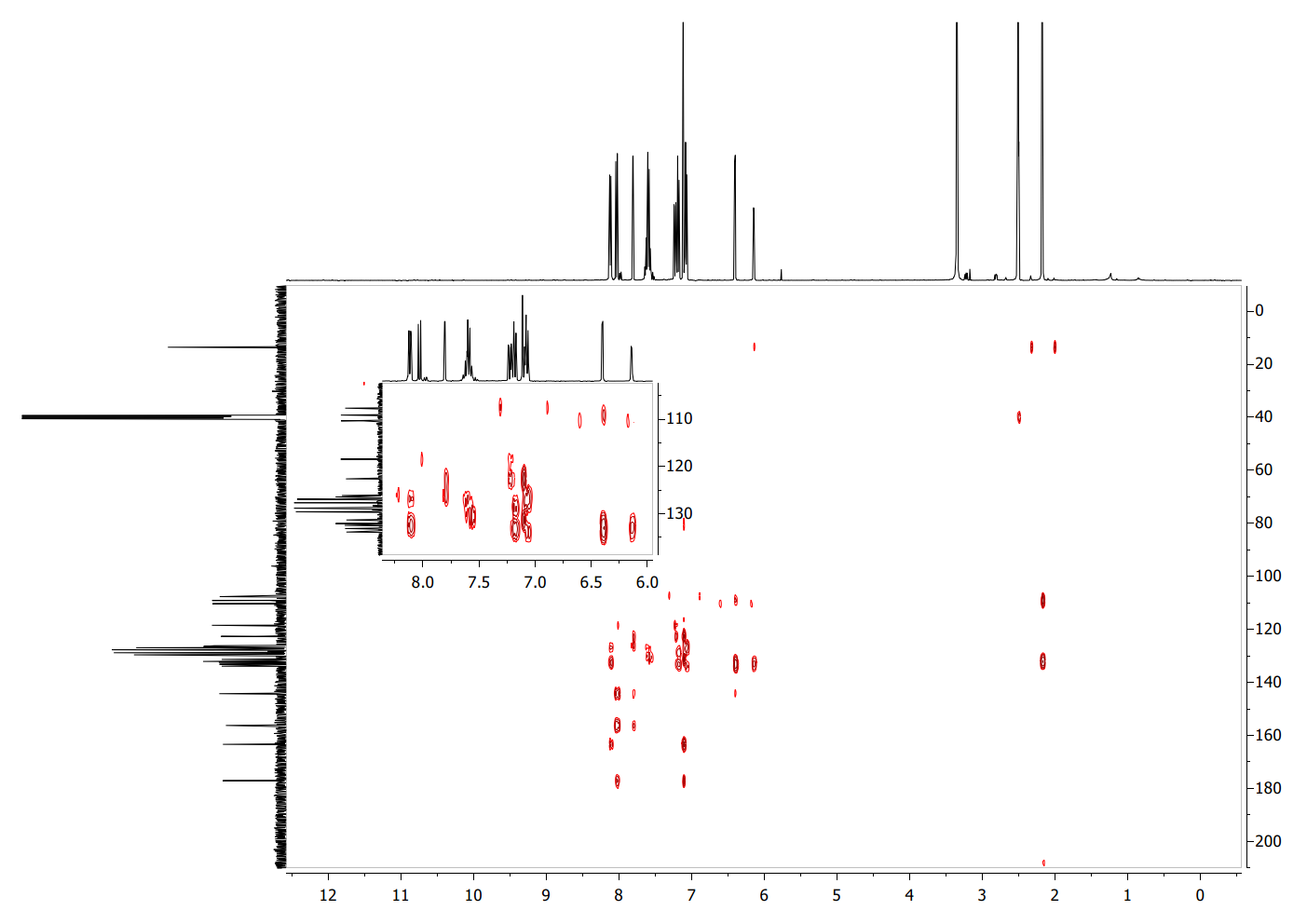

^1^H^13^C HMBC NMR spectrum of **5b** (DMSO-*d_6_*, 25°C).

**Diethyl 2,5-dimethyl-1-(4-oxo-2-phenyl-chromen-7-yl)pyrrole-3,4-dicarboxylate (5c)**


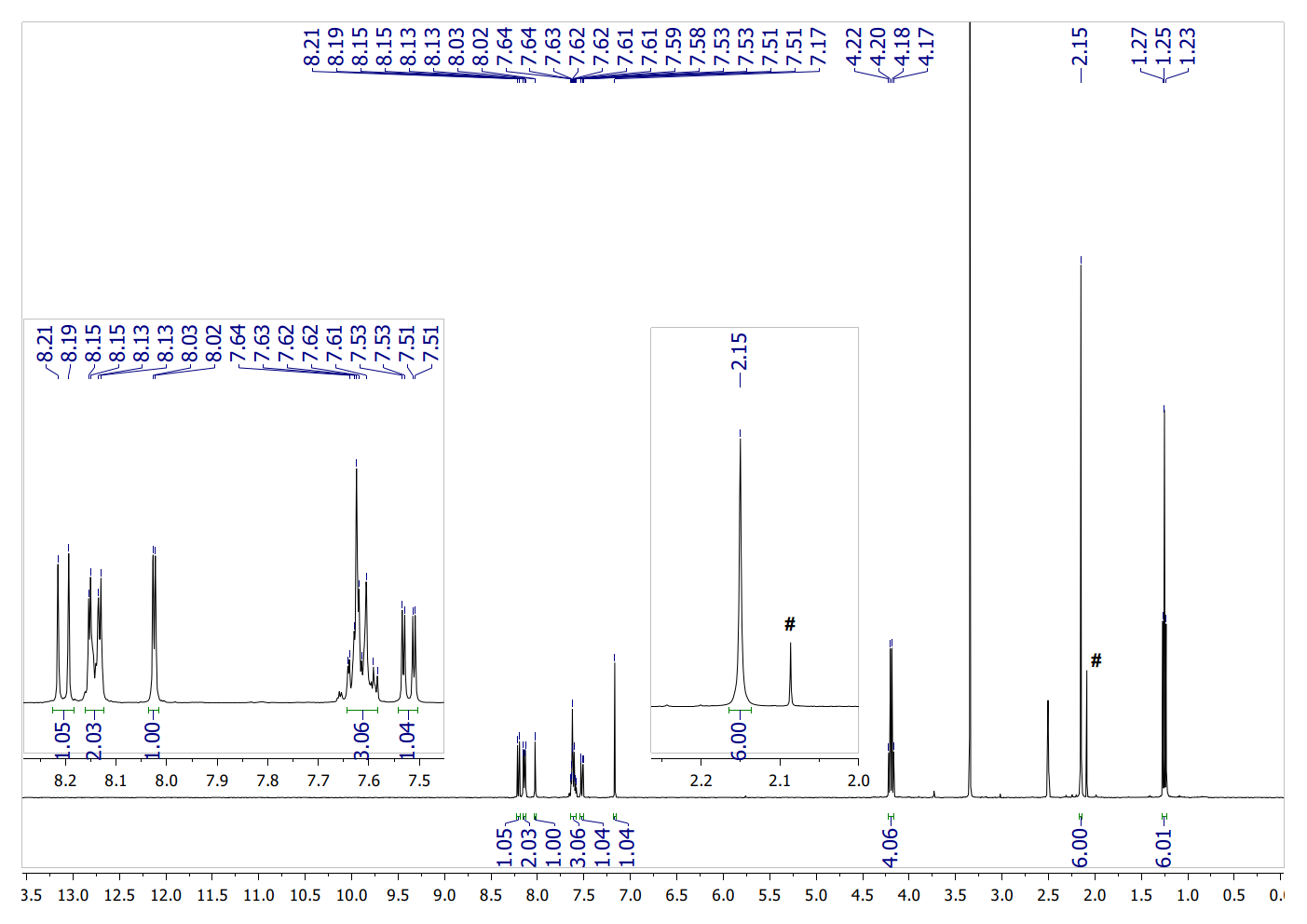

^1^H NMR spectrum of **5c** (DMSO-*d_6_*, 25°C) # - residual peak from acetone.


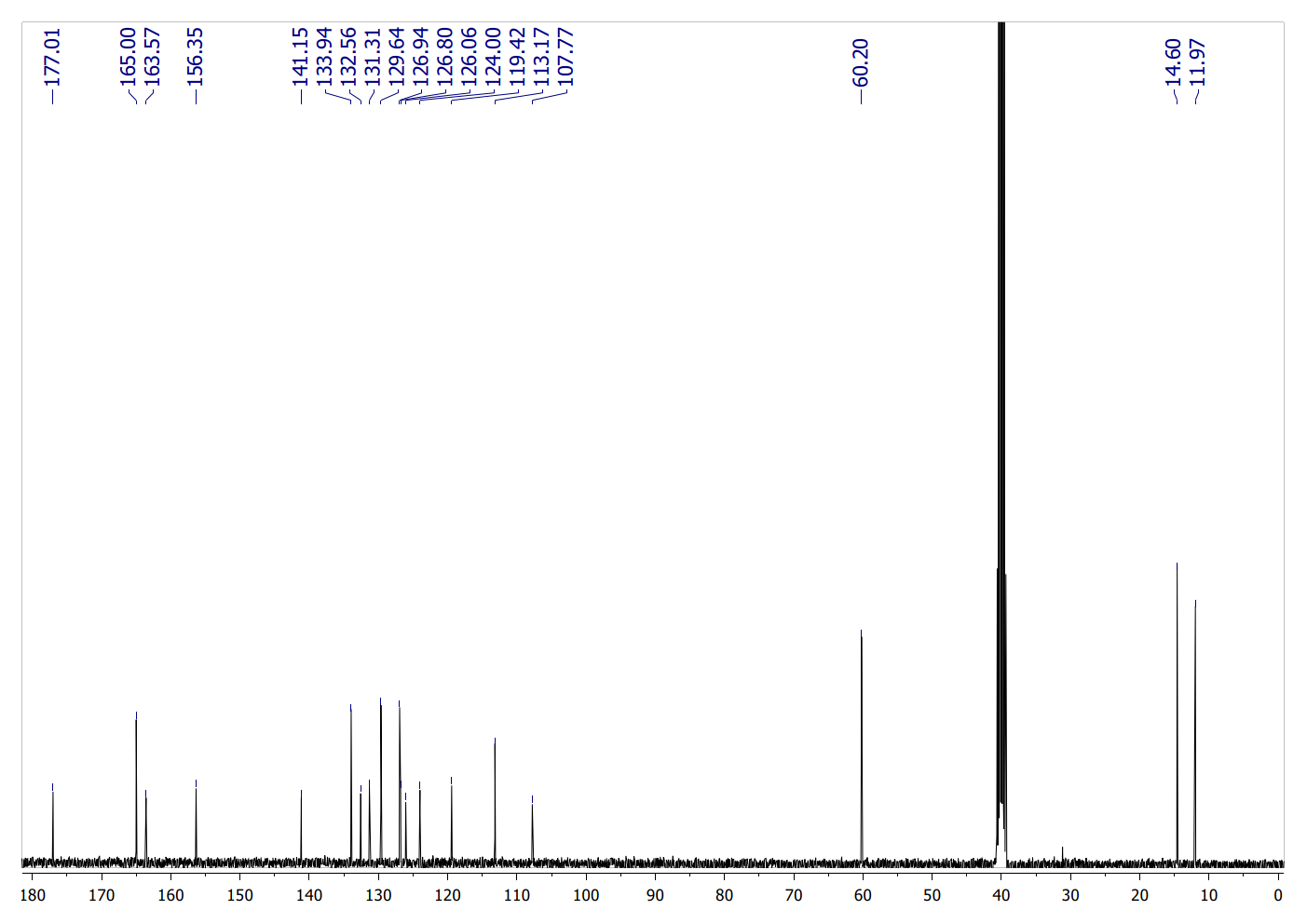

^13^C NMR spectrum of **5c** (DMSO-*d_6_*, 25°C).


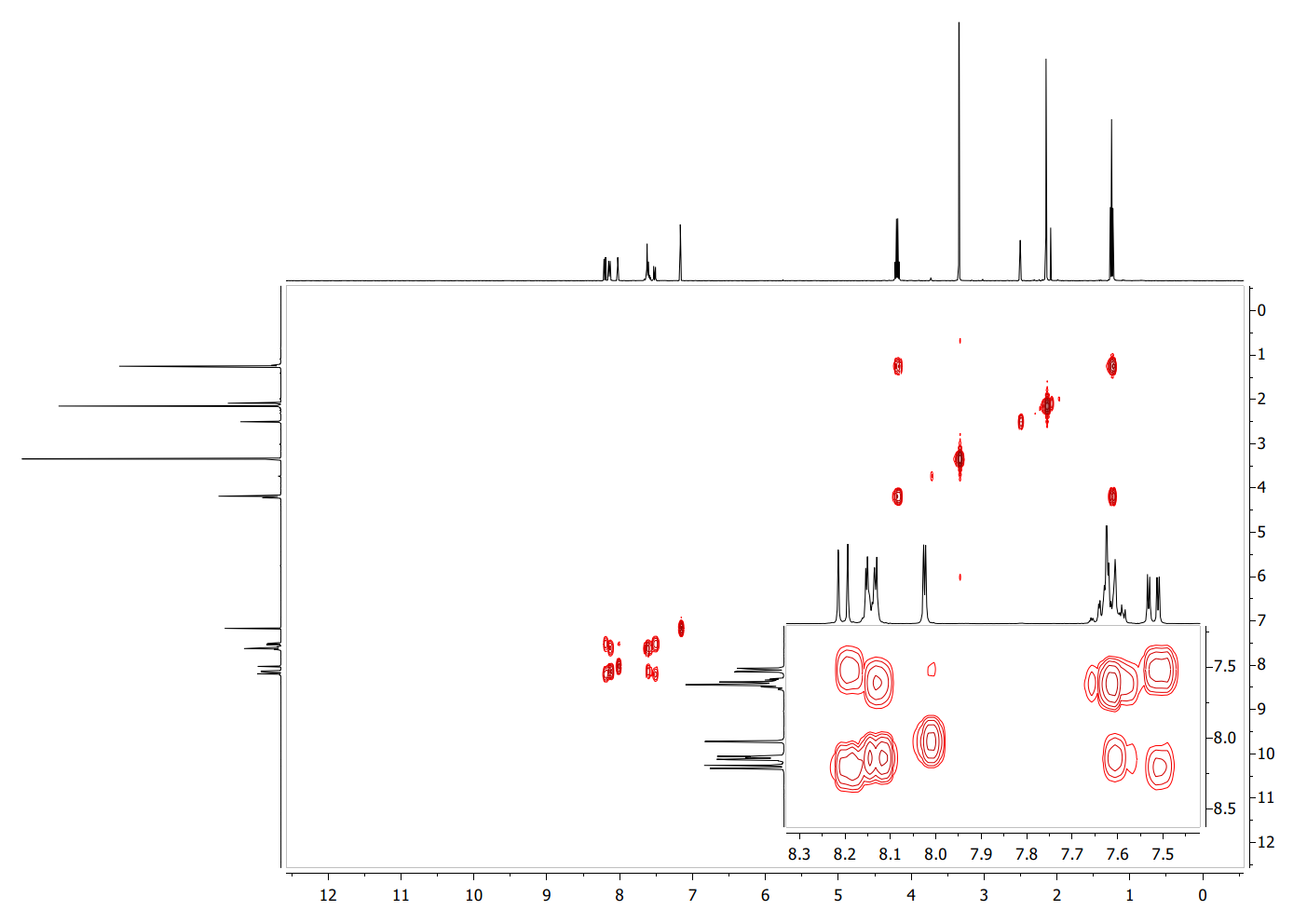

^1^H^1^H COSY NMR spectrum of **5c** (DMSO-*d_6_*, 25°C).


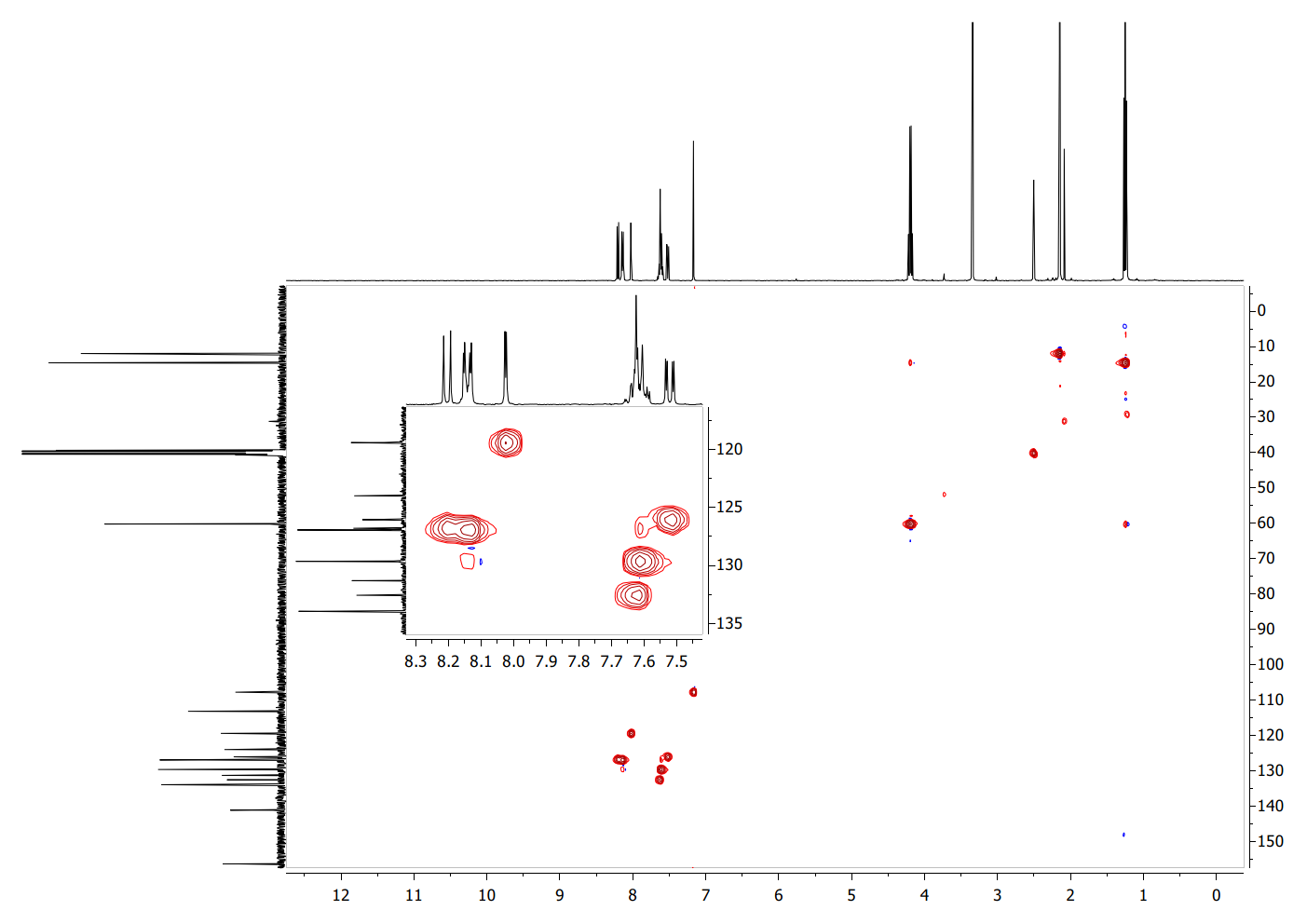

^1^H^13^C HSQC NMR spectrum of **5c** (DMSO-*d_6_*, 25°C).


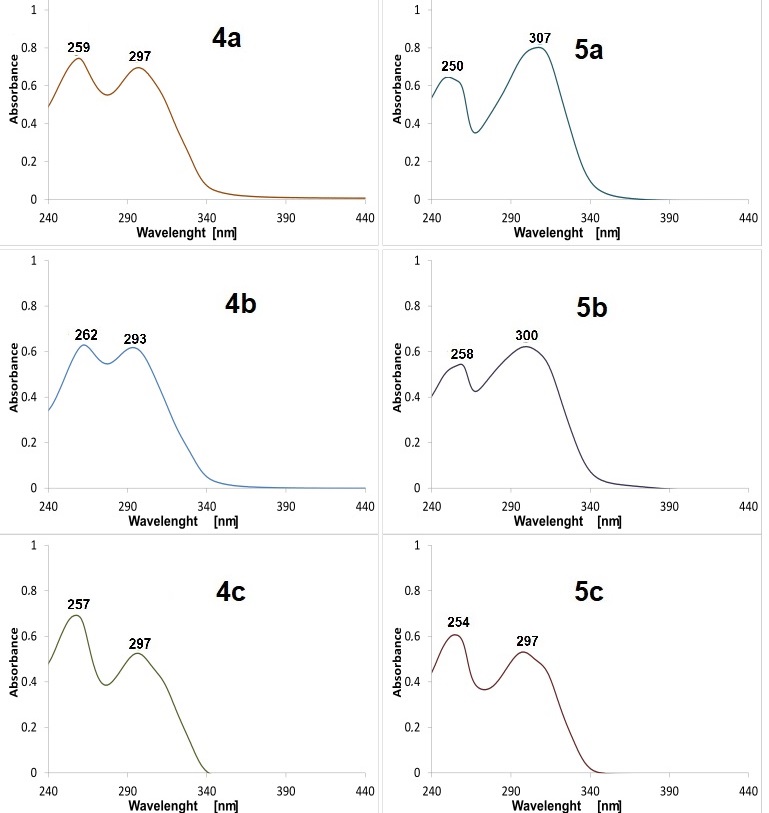


**Figure S5**. UV-VIS spectra recorded for **4a-c** and **5a-c** in methanol.
